# A revision of *Meladema* diving beetles (Coleoptera, Dytiscidae), with the description of a new species from the central Mediterranean based on molecules and morphology

**DOI:** 10.3897/zookeys.702.14787

**Published:** 2017-09-25

**Authors:** David T. Bilton, Ignacio Ribera

**Affiliations:** 1 Marine Biology and Ecology Research Centre, School of Biological and Marine Sciences, University of Plymouth, Plymouth PL4 8AA, UK; 2 Institut de Biologia Evolutiva (CSIC-Universitat Pompeu Fabra), Passeig Marítim de la Barceloneta 37-49, 08003 Barcelona, Spain

**Keywords:** Systematics, integrative taxonomy, biogeography, cryptic species, freshwater, biodiversity, entomology

## Abstract

*Meladema* Laporte, 1835 are relatively large, stream-dwelling diving beetles, distributed widely in the Western Palaearctic, from the Atlantic Islands to Turkey, and from southern France and the Balkans to the central Sahara. In addition to the three previously recognised taxa (*M. coriacea* Laporte, 1835, *M. imbricata* (Wollaston, 1871) and *M. lanio* (Fabricius, 1775)) we describe a new, cryptic, species from the central Mediterranean area, which can be distinguished from *M. coriacea* on both DNA sequence data and morphology, and provide a key to known species of the genus. Based on the study of genotyped material, both recent and archival, as well as the examination of a large number of museum specimens, we show that *M. lepidoptera* **sp. n.** occurs to the apparent exclusion of *M. coriacea* on Corsica, Sardinia and islands of the Tuscan Archipelago, but that both taxa are found in peninsular Italy, where they may occasionally hybridize. In the absence of the original type series, we designate a neotype for *M. coriacea*, and take the opportunity to designate a lectotype for *M. lanio*. Morphological variation in *Meladema* species is discussed, including that seen in known and presumed hybrids. Our study highlights the incomplete state of knowledge of Mediterranean biodiversity, even in relatively large, supposedly well-studied taxa.

## Introduction

*Meladema* Laporte, 1835 is a small genus of large diving beetles, found in streams in the Western Palaearctic, from the Canary Islands and Madeira, to western Turkey (Sharp 1882, Guignot 1932, Franciscolo 1979, Balke et al. 1989, Balke et al. 1990, Ribera et al. 2003, Darilmaz and Kiyak 2009, Touaylia et al. 2011, Sýkora et al. 2017). Species of the genus are particularly characteristic of deeper, (semi) permanent pools, and may often function as top predators in fishless streams on the Atlantic Islands, and in temporary Mediterranaean systems. At present the genus contains three species: the widespread *Meladema coriacea* Laporte, 1835, distributed from the Canary Islands to Turkey and ranging from southern France and the central Balkans south to the central Sahara, and two Atlantic Island endemics, *Meladema imbricata* (Wollaston, 1871) from the western Canary Islands and *Meladema lanio* (Fabricius, 1775) from the main island of Madeira.

Ribera et al. (2003) studied the phylogeny and phylogeography of *Meladema* using mitochondrial DNA sequences, and demonstrated that the genus contains four divergent mtDNA clades, two corresponding to the Atlantic Island species, the other two nesting within *M. coriacea*; specimens from the island of Corsica being highly divergent from all other material examined from the Canaries, Iberia, the Balearic Islands, southern France and North Africa. The two lineages within *M. coriacea* were thought to be morphologically identical, however, without apparent differences in characters habitually used in the species level-taxonomy of Dytiscidae, including male genitalia. Subsequently, Sýkora et al. (2017) surveyed both mitochondrial and nuclear DNA sequence variation across a wider range of localities, confirming the presence of these four genetic lineages, *M. coriacea* being divided (on both mitochondrial and nuclear markers) into a widespread clade, distributed from the Canary Islands to Turkey, and another clade, restricted in their analyses to Corsica, Sardinia and Montecristo (termed ‘*coriacea* CSM’). Molecular dating analyses suggested that *Meladema* originated in the Middle Miocene, approximately 14.4 million years ago (MYA; 95% CI 10-20 MYA), with all four extant lineages dating from the early Pleistocene ca. 1.2-1.5 MYA (Sýkora et al. 2017). Ribera et al. (2003) and Sýkora et al. (2017) refrained from describing ‘*coriacea* CSM’ as a distinct species, despite its genetic divergence, since it was apparently morphologically identical to other populations of *M. coriacea*.

We have reexamined morphological variation in *Meladema* in the light of these recent molecular results, and demonstrate that whilst ‘*coriacea* CSM’ cannot be distinguished from other *M. coriacea* using male genital anatomy, these two lineages can be separated reliably on the basis of differences in the elytral sculpture of both sexes. By studying a combination of newly genotyped specimens and extensive museum material, we show that ‘*coriacea* CSM’, here described as *M. lepidoptera* sp. n., occurs on the Tyrrhenian Islands (Corsica, Sardinia, Elba, Montecristo) and on the Italian mainland, where it comes into contact with *M. coriacea*. Since the type series of *M. coriacea* could not be located, and is likely destroyed (Evenhuis 2012), we designate a neotype for this species in the interests of stability, using a genotyped specimen from southern France, where Laporte’s material originated. We also take the opportunity to designate a lectotype for *M. lanio*, and discuss character variation in the genus, including that seen in known or presumed hybrid individuals from the Atlantic Islands and Italy. Our work highlights the incomplete state of knowledge of Mediterranean biodiversity, where even relatively large, well-known taxa can hide previously unrecognised species (see also Audisio et al. 2009, Zauli et al. 2016).

## Materials and methods

### Morphology

**Figure 1.**
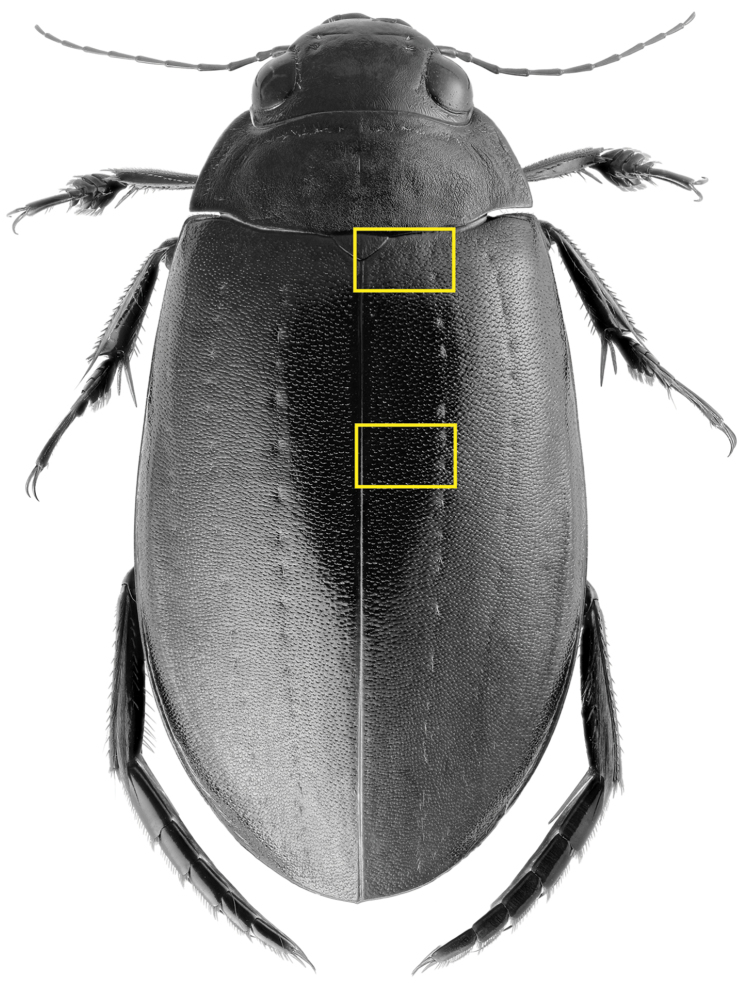
Dorsal view of *Meladema coriacea*, showing regions in which scanning electron and light micrographs of elytral sculpture were made.

Specimens were studied with Leica MZ8 and M205C stereomicroscopes at x8–100, illuminated with a Fluopac FP1 flourescent light, or a swan-neck illuminator diffused using a tracing paper collar close to the specimen (to enable study of microsculpture). A wide range of morphological characters were initially compared across genotyped material of *M. coriacea* and *M. lepidoptera* sp. n., in the search for diagnostic features. These included dorsal and ventral sculpture of both sexes and secondary sexual characters (male and female genitalia and last abdominal ventrites, male tarsal modifications). Digital photographs were taken with a Canon EOS 500D camera with a Sigma 50mm f/2.8 EX DG macro lens, illuminated with two Fluopac FP1 flourescent lights (habitus photos) or with a Leica Z6 Apo macroscope, fitted with a 2x objective lens illuminated using a Leica LED5000 HDI dome illuminator to avoid shadow (all other features). Male and female genitalia were studied wet, temporarily mounted in alcohol-based hand sanitizer gel to stabilize their position during image stacking. Image stacks were produced by hand, and combined using Zerene Stacker software (www.zerenesystems.com). For scanning electron microscopy material was degreased for two days in 100% acetone and air-dried overnight at 60°C, before being mounted onto metal stubs using double-sided carbon conducting tape. Specimens were gold sputter coated using an Emitech K550 Coating Unit, then examined and photographed in a JEOL JSM6610LV Scanning Electron Microscope (SEM). Elytral sculpture was typically imaged at the shoulder and in the centre, close to the suture (Figure 1), these positions being chosen following initial screening described above.

The terminology to denote the orientation of male genitalia follows Miller and Nilsson (2003). Female reproductive tracts were prepared as follows: the last three abdominal segments were removed from ethanol preserved material and rehydrated for 10 min in distilled water. Terga were opened with dissecting scissors, and the whole abdomen macerated for 20 min in 10% aqueous potassium hydroxide at 30°C. The reproductive tract and associated sclerites were then removed from the abdomen and redigested in 10% potassium hydroxide at 30°C for a further 10 mins. Following this they were placed in 10% acetic acid for 1 min at room temperature, before being transferred to 70% ethanol prior to examination.

Exact label data for specimens are cited in quotation marks; separate quotes for the same specimen indicate separate labels. A double slash (//) indicates separate label lines. All descriptions are based on genotyped material unless otherwise stated.

### Molecular data and analyses

We added newly sequenced specimens from mainland Italy, some Tyrrhenian islands and North Africa (Table 1) to the molecular dataset of Sýkora et al. (2017). DNA was extracted non-destructively with commercial kits (“DNeasy Tissue Kit”, Qiagen GmbH, Hilden, Germany) following the manufacturer’s instructions. DNA extractions are retained in the collections of the Institut de Biologia Evolutiva, Barcelona (IBE). Fresh specimens were extracted following the methods detailed in Sýkora et al. (2017). Dry preserved specimens were first soaked for 1 h in a 10% SDS solution at 35°C. Following this, the beetle was carefully removed from its mount or pin, placed in a new, sterile petri-dish containing ultrapure water, and the genitalia and associated tissues removed with watchmaker’s forceps. All specimen manipulation was conducted in a fume hood, in a laboratory which never handles DNA samples. Forceps were dipped in 100% ethanol and flamed between samples, and fresh vinyl gloves were worn to handle each beetle. Following tissue extraction, the specimen was air dried and remounted. DNA extractions and amplifications of tissues from dry or badly preserved specimens were conducted under a fume hood, with filter tips and fresh primer and chemical aliquotes for each specimen, to prevent contamination. Following DNA extraction, genital structures were mounted beside the specimen, or on a card on the same pin.

**Table 1.** Specimens of *Meladema* used in genetic analyses, with DNA voucher, locality, collector and accession numbers of available sequences (newly obtained sequences in bold). The COI-3’ sequence of specimen IBE-AN691 is of very low quality and was not submitted. Neotype of *M. coriacea* and holotype of *M. lepidoptera* sp. n. indicated with asterisks (all other specimens of *M. lepidoptera* sp. n. are paratypes). See text for full label data.

Taxon	Voucher	Country/Island	Locality	Collector	COI-5’ (barcode)	COI-3’	16S+tRNA-L +nad1	H3	WG
*coriacea*	IBE-AN691	Sicily	Bosco Ficuzzo	M.Toledo		x			
*coriacea*	IBE-AN739	Chad	Tibesti, Koudou	Bruneau de Miré				**LT898148**	
*coriacea*	IBE-DV291	Spain	Cáceres, PN Monfragüe	I.Ribera & P.Abellán	**LT898152**	LT602719	LT602827	LT602736	LT602782
*coriacea*	IBE-DV292	Spain	Huesca, Bco. de Bernués	I.Ribera & A.Cieslak	**LT898153**	LT602720	LT602828	LT602737	LT602783
*coriacea*	IBE-DV293	Spain	Girona, Port Bou	I.Ribera & A.Cieslak	**LT898154**	LT602721	LT602829	LT602738	LT602784
*coriacea*	IBE-DV294	Turkey	Izmir, Phoca	I.Ribera & A.Cieslak	**LT898155**	LT602722	LT602830	LT602739	LT602785
*coriacea*	IBE-RA1064	Malta	between Mosta and L-Imtarfa	A.Rudoy		LT602725	LT602833	LT602740	LT602786
*coriacea*	MNCN-AI104	Spain	Corbera d’Ebre, riu Gaia	I.Ribera		LT602727	LT602835	LT602743	LT602789
*coriacea*	MNCN-AI1095	Tenerife	Chamorga, Bco. Roque Bermejo	A.Castro		LT602730	LT602838	LT602744	LT602790
*coriacea*	MNCN-AI84	Tunisia	Cite el Morjne	M.G.París	**LT906387**	LT602726	LT602834	LT602745	LT602791
*coriacea*	MNCN-AI860	Spain	Castellón, Ballestar	I.Ribera		LT602728	LT602836	LT602746	LT602792
*coriacea*	MNCN-AI861	Spain	Castellón, Ballestar	I.Ribera		LT602729	LT602837		
*coriacea*	MNCN-HI4	Algeria	Oued Bagrat	S.Bouzid		LT602731	LT602839	LT602747	LT602793
*coriacea*	MNCN-HI6	Algeria	Aïn Damous	S.Bouzid		LT602732	LT602840	LT602748	LT602794
*coriacea*	NHM-IR47	Morocco	Taza, Tazzeka N.P.	I.Ribera				EF670124	
*coriacea*	NHM-IRM10a	Spain	Cádiz, Fancinas	I.Ribera		AF428215	AF428189		
*coriacea*	NHM-IRM11a	France	Var, La Londe-les-Maures	P.Ponel		AF428208	AF428189		
*coriacea*	NHM-IRM11b	France	Var, La Londe-les-Maures	P.Ponel		AF428209	AF428189	LT602749	LT602795
coriacea*	NHM-IRM11c	France	Var, La Londe-les-Maures	P.Ponel		AF428207	AF428189		
*coriacea*	NHM-IRM13a	Spain	Murcia, Fte. Caputa	A.Millán		AF428218	AF428189	LT602753	LT602798
*coriacea*	NHM-IRM14a	Spain	Córdoba, Baena, Arroyo de las Beatas	M.Baena		AF428216	AF428190	LT602754	LT602799
*coriacea*	NHM-IRM14b	Spain	Córdoba, Baena, Arroyo de las Beatas	M.Baena		AF428216	AF428190		
*coriacea*	NHM-IRM14c	Spain	Córdoba, Baena, Arroyo de las Beatas	M.Baena		AF428217	AF428189		
*coriacea*	NHM-IRM18a	Tenerife	Bco. Del Infierno	D.T.Bilton		AF428222	AF428189	LT602757	LT602802
*coriacea*	NHM-IRM19a	Tenerife	Bco. De Masca	D.T.Bilton		AF428222	AF428189	LT602758	LT602803
*coriacea*	NHM-IRM19b	Tenerife	Bco. De Masca	D.T.Bilton		AF428222	AF428189	LT602759	LT602804
*coriacea*	NHM-IRM1a	Morocco	Taza, Tazzeka N.P.	I.Ribera		AF428212	AF428189	LT602760	LT602805
*coriacea*	NHM-IRM1c	Morocco	Taza, Tazzeka N.P.	I.Ribera		AF428213	AF428189		
*coriacea*	NHM-IRM20a	Gran Canaria	S. Nicolas de Tolentino, bco. Guy Guy grande	I.Ribera & A.Cieslak		AF428221	AF428189	LT602761	LT602806
*coriacea*	NHM-IRM21a	Morocco	Immouzèr-des-Ida-Outanane, Assif Tanit	I.Ribera & A.Cieslak		AF428214	AF428189	LT602762	LT602807
*coriacea*	NHM-IRM21b	Morocco	Immouzèr-des-Ida-Outanane, Assif Tanit	I.Ribera & A.Cieslak		AF428215	AF428189		
*coriacea*	NHM-IRM22a	Morocco	Tachokchte, Assif Siroua	I.Ribera & A.Cieslak		AF428216	AF428189	LT602763	LT602808
*coriacea*	NHM-IRM22b	Morocco	Tachokchte, Assif Siroua	I.Ribera & A.Cieslak		AF428216	AF428189		
*coriacea*	NHM-IRM23a	Mallorca	Mortixet, Te. Son March	I.Ribera & A.Cieslak		AF428219	AF428191	LT602764	LT602809
*coriacea*	NHM-IRM23b	Mallorca	Mortixet, Te. Son March	I.Ribera & A.Cieslak		AF428220	AF428189		
*coriacea*	NHM-IRM24a	Mallorca	Els Casals, Te. Son March	I.Ribera & A.Cieslak		AF428215	AF428189		
*coriacea*	NHM-IRM2a	Morocco	Anti Atlas, Oued Massa	I.Ribera		AF428210	AF428189		
*coriacea*	NHM-IRM2b	Morocco	Anti Atlas, Oued Massa	I.Ribera		AF428211		LT602765	LT602810
*imbricata*	NHM-IRM15a	Gomera	El Cedro	D.T.Bilton		AF428224	AF428192	LT602766	LT602811
*imbricata*	NHM-IRM15b	Gomera	El Cedro	D.T.Bilton		AF428225	AF428192	LT602767	LT602812
*imbricata*	NHM-IRM17a	Tenerife	Bco. del Río	D.T.Bilton	**KJ637881**	AF428228	KJ637898	KJ638011	
*imbricata*	NHM-IRM17b	Tenerife	Bco. del Río	D.T.Bilton		AF428230	AF428192	LT602768	LT602813
*imbricata*	NHM-IRM3a	Gomera	El Cedro	D.T.Bilton		AF428224	AF428192	LT602770	LT602815
*imbricata*	NHM-IRM4a	Gomera	El Cedro	D.T.Bilton		AF428224	AF428192	LT602771	LT602816
*imbricata*	NHM-IRM4b	Gomera	El Cedro	D.T.Bilton		AF428224	AF428192	LT602772	LT602817
*imbricata*	NHM-IRM5a	Tenerife	Bco. del Río	D.T.Bilton		AF428230	AF428192	LT602773	LT602818
*imbricata*	NHM-IRM5d	Tenerife	Bco. del Río	D.T.Bilton		AF428229			
*imbricata*	NHM-IRM6a	La Palma	Bco. del Hoyo Verde	D.T.Bilton		AF428227			
*imbricata*	NHM-IRM6b	La Palma	Bco. del Hoyo Verde	D.T.Bilton		AF428227	AF428192	LT602775	LT602820
*imbricata*	NHM-IRM6c	La Palma	Bco. del Hoyo Verde	D.T.Bilton		AF428227	AF428192	LT602776	LT602821
*imbricata*	NHM-IRM7a	La Palma	Bco. del Rio above Santa Cruz	D.T.Bilton		AF428226	AF428192		
*lanio*	IBE-DV298	Madeira	Canhas, Paul da Serra	A.Rudoy	**LT898156**	LT602733	LT602841	LT602777	LT602822
*lanio*	NHM-IRM8a	Madeira	Ribera dos Cedros	L.C.Kelly		AF428233	AF428194	LT602778	LT602823
*lanio*	NHM-IRM9a	Madeira	Levada das Faias	L.C.Kelly		AF428231	AF428194		
*lanio*	NHM-IRM9b	Madeira	Levada das Faias	L.C.Kelly		AF428232	AF428194	LT602779	LT602824
*lepidoptera* sp. n.	IBE-AN692	Elba	Pomonte, Fosco Barione	M.Toledo		**LT898157**			
*lepidoptera* sp. n.	IBE-AN693	Italy	Toscana, S. Luce	M.Toledo		**LT898158**		**LT898147**	
*lepidoptera* sp. n.	IBE-AN760	Italy	Monti della Tolfa	V.Buono	**LT898149**	**LT898159**			
*lepidoptera* sp. n.	IBE-DV289	Montecristo		R.Vila	**LT898150**	LT602717	LT602825	LT602734	LT602780
*lepidoptera* sp. n.	IBE-DV290	Montecristo		R.Vila	**LT898151**	LT602718	LT602826	LT602735	LT602781
*lepidoptera* sp. n.	IBE-RA18	Sardinia	Ogliastra, ca. 5 km WNW Tortoli	H.Fery & M.Toledo		LT602724	LT602832	LT602741	LT602787
*lepidoptera* sp. n.	IBE-RA5	Sardinia	Nuoro prov., Villagrande Strìsaili	H.Fery & M.Toledo		LT602723	LT602831	LT602742	LT602788
*lepidoptera* sp. n.	NHM-IRM12a	Corsica	Porto-Vecchio, l’Ospedale	I.Ribera & A.Cieslak	**LT906388**	AF428203			
*lepidoptera* sp. n.	NHM-IRM12b	Corsica	Ghisoni, road to Campannella	I.Ribera & A.Cieslak		AF428204	AF428187	LT602750	
*lepidoptera* sp. n.	NHM-IRM12c	Corsica	Cap Corse, Bettolacce	I.Ribera & A.Cieslak	**LT906389**	AF428205	AF428188	LT602751	LT602796
*lepidoptera* sp. n.	NHM-IRM12d	Corsica	Porto-Vecchio, l’Ospedale	I.Ribera & A.Cieslak	**LT906390**	AF428203	AF428187	LT602752	LT602797
*lepidoptera* sp. n.*	NHM-IRM12e	Corsica	Cap Corse, Bettolacce	I.Ribera & A.Cieslak		AF428206	AF428188		
*lepidoptera* sp. n.	NHM-IRM12f	Corsica	Cap Corse, Bettolacce	I.Ribera & A.Cieslak	**LT906391**	AF428207	AF428187		
*lepidoptera* sp. n.	NHM-IRM12g	Corsica	Porto-Vecchio, l’Ospedale	I.Ribera & A.Cieslak		AF428203	AF428187		
*coriacea*x*imbricata*	NHM-IRM16a	Tenerife	Bco. del Río	D.T.Bilton		AF428223	AF428192	LT602755	LT602800
*coriacea*x*imbricata*	NHM-IRM16b	Tenerife	Bco. del Río	D.T.Bilton		AF428223	AF428192	LT602756	LT602801
*imbricata*x*coriacea*	NHM-IRM5b	Tenerife	Bco. del Río	D.T.Bilton		AF428222	AF428189		
*imbricata*x*coriacea*	NHM-IRM5c	Tenerife	Bco. del Río	D.T.Bilton		AF428222	AF428189	LT602774	LT602819
*imbricata*x*coriacea*	NHM-IRM17c	Tenerife	Bco. del Río	D.T.Bilton		AF428222	AF428189	LT602769	LT602814

We amplified fragments of the Cytochrome Oxidase Subunit 1 mitochondrial gene (5’ end, COI-5’, and 3’ end, COI-3’) and an internal fragment of the nuclear gene Histone 3 (H3) (see Table 2 and Sýkora et al. (2017) for details on primers used and PCR cycling conditions). Attempts to amplifly additional gene fragments used in Sýkora et al. (2017) from dry material were not succesful (see Results below). New sequences have been deposited in Gen Bank with accession numbers LT898147–LT898159 and and LT906387–LT906391 (Table 1).

To place newly sequenced specimens in a phylogenetic context we included them in a matrix with the COI data from Sýkora et al. (2017), and analysed it with a fast Maximum Likelihood heuristic algorithm in RAxML-HPC2 in the CIPRES Science Gateway (Miller et al. 2010), using a single partition with a GTR+G evolutionary model and assessing node support with 100 pseudoreplicates of a rapid bootstrapping algorithm (Stamatakis et al. 2008).

**Table 2.** Primers used for amplification and sequencing. In brackets, length of the amplified fragment.

**Gene**	**Primer**	**Sequence**	**Reference**
COI-3’	Jerry (5’)	CAACATTTATTTTGATTTTTTGG	Simon et al. (1994)
(826)	Pat (3’)	TCCAATGCACTAATCTGCCATATTA	Simon et al. (1994)
	Chy (5’)	T(A/T)GTAGCCCA(T/C)TTTCATTA(T/C)GT	Ribera et al. (2010)
	Tom (3’)	AC(A/G)TAATGAAA(A/G)TGGGCTAC(T/A)A	Ribera et al. (2010)
COI-5’	LepF1b	ATTCAACCAATCATAAAGATATTGGAAC	Dincă et al. (2017)
(658)	LepR1	TAAACTTCTGGATGTCCAAAAAATCA	Hebert et al. (2003)
H3	H3aF (5’)	ATGGCTCGTACCAAGCAGACRCG	Colgan et al. (1998)
(327)	H3aR (3’)	ATATCCTTRGGCATRATRGTGAC	Colgan et al. (1998)

### Abbreviations

**BMNH** Natural History Museum, London, UK

**CBF** Collection H. Bussler, Freising, Germany

**CBP** Collection D.T. Bilton, Plymouth, UK

**CFA** Collection G.N. Foster, Ayr, UK (to be deposited in Hunterian Museum, Glasgow University, Glasgow, UK)

**CTP** Collection M. Toledo, Parma, Italy

**CVR** Collection V. Volpe, Roma, Italy

**IBE** Institut de Biologia Evolutiva, Barcelona, Spain

**ISNB** Institut royal des Sciences naturelles de Belgique, Brussels, Belgium

**MNCN** Museo Nacional de Ciencias Naturales, Madrid, Spain

**MNHN** Muséum national d’Histoire naturelle, Paris, France

**NMW** Naturhistorisches Museum, Wien, Austria

**ZSM** Zoologische Staatssammlung, München, Germany

**TL** Total length, front of head to elytral apices

**EL** Elytral length

**MW** Maximum width, elytra

**HW** Handwriting

## Results

We obtained enough sequence data from the COI gene to allow an unambiguous phylogenetic placement of two specimens from mainland Italy (Toscana and Lazio, DNA vouchers IBE-AN693 and IBE-AN760 respectively; Table 1) and one from Elba (IBE-AN692). We also obtained partial COI data from one specimen from Sicily (IBE-AN691) allowing its identity to be established and nuclear (H3) data from one specimen from the Tibesti, in Chad (IBE-739), which included diagnostic positions allowing some discrimination, but not an unambiguous species identification. We could not obtain any sequences from two of the dry specimens extracted, IBE-AN694 (mainland Italy, Campania) and IBE-AN740 (Chad, Tibesti).

**Figure 2.**
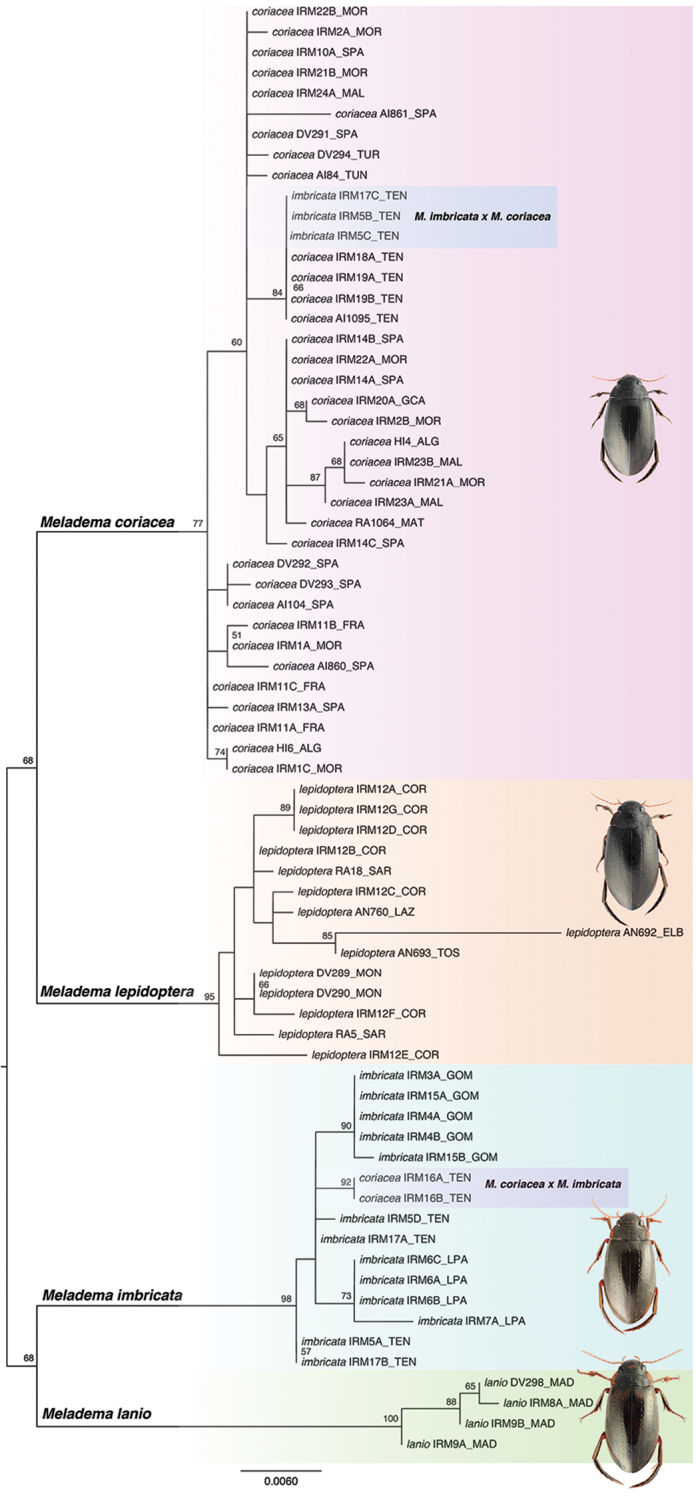
Phylogram obtained from analysis of the COI-3’ fragment in RAxML. Numbers on nodes, bootstrap support values. See Table 1 for specimen and locality codes. Habituses correspond to those in Figure 3.

In the RAxML analysis with the COI-3’ marker the two sequenced specimens from mainland Italy and the one from Elba were clearly clustered with other specimens from Corsica, Sardinia and Montecristo, with strong bootstrap support (Figure 2). Although only a limited number of specimens were sequenced for the COI-5’ fragment (Table 1), there was also a clear segregation of *M. coriacea*/*lepidoptera* sp. n. haplotypes into two groups, fully congruent with that seen with the COI-3’ fragment.

For the Sicilian specimen (IBE-AN691) it was only possible to obtain a low quality partial sequence for the reverse primer of the 3’ end of the COI gene (ca. 664 nucleotides, primer Jerry, see Table 2). Of the six diagnostic positions separating *M. coriacea* from *M. lepidoptera* sp. n. in this gene fragment, four were apparently *M. coriacea* and two *M. lepidoptera* sp. n. This specimen had been previously unambiguously assigned to *M. coriacea* on external morphology.

We obtained the H3 sequence from one of the specimens from the Tibesti (AN739, Table 1), although it was not possible to sequence any other markers from this specimen. The H3 sequence was identical to the sequence of all *M. coriacea* + *lepidoptera* sp. n. and different from *M. imbricata* and *M. lanio* in the single diagnostic position in this gene fragment (see Sýkora et al. 2017).

## Taxonomy

### Meladema

Taxon classificationAnimaliaColeopteraDytiscidae

Laporte, 1835

Meladema Laporte, 1835:98, gender feminine; type species: Meladema coriacea Laporte, 1835:98, by monotypy; conserved in ICZN Opinion 1725 (ICZN 1993). Scutopterus Dejean, 1833:54, unavailable name, rejected in Opinion 1725 (ICZN 1993).

#### Diagnosis.

Adults can be recognised within the Colymbetinae on the following combination of characters: pronotal beading absent; protibiae only weakly emarginate basoventrally; prosternal process medially rounded; anterior margin of metaventrite deeply incised for reception of prosternal process; metatarsomeres I-IV distinctly sinuate apically, with apicolateral lobes and metatarsal claws subequal in length, outer approximately two-thirds length of inner (Miller and Bergsten 2016). The sculpture of the elytra referred to by Miller and Bergsten (2016) is not a constant generic character of *Meladema* (see below). Larvae of two species (*M. coriacea* and *M. lanio*) have been described to date (Bertrand 1928, 1932a, 1932b, Falkenström 1938, Nilsson and Hilsenhoff 1991, Alarie and Hughes 2006). First instar larvae of *Meladema* can be distinguished from most other genera of Colymbetinae on: Additional setae present on dorsal margin of femur; short, spine-like mesofemoral seta FE5; additional setae on both ventral and dorsal margins of tibiae and relatively large size (Alarie and Hughes 2006). They can be separated from *Bunites* Spangler, 1972 on details of the setation of the coxae and abdominal segment 8 (Michat 2005). Third instar larvae are characterized by: relatively large size (head length > 4.40 mm cf. < 3.50 mm in other described Colymbetinae); urogomphus more elongate (> 1.7× dorsal length of last abdominal segment cf. < 1.5×); large number of secondary setae on legs and the presence of predominantly short and spine-like setae on outer and elongate setae with hair-like secondary setae along inner margin of urogomphus (see Alarie and Hughes 2006).

#### Description.

**Figure 3.**
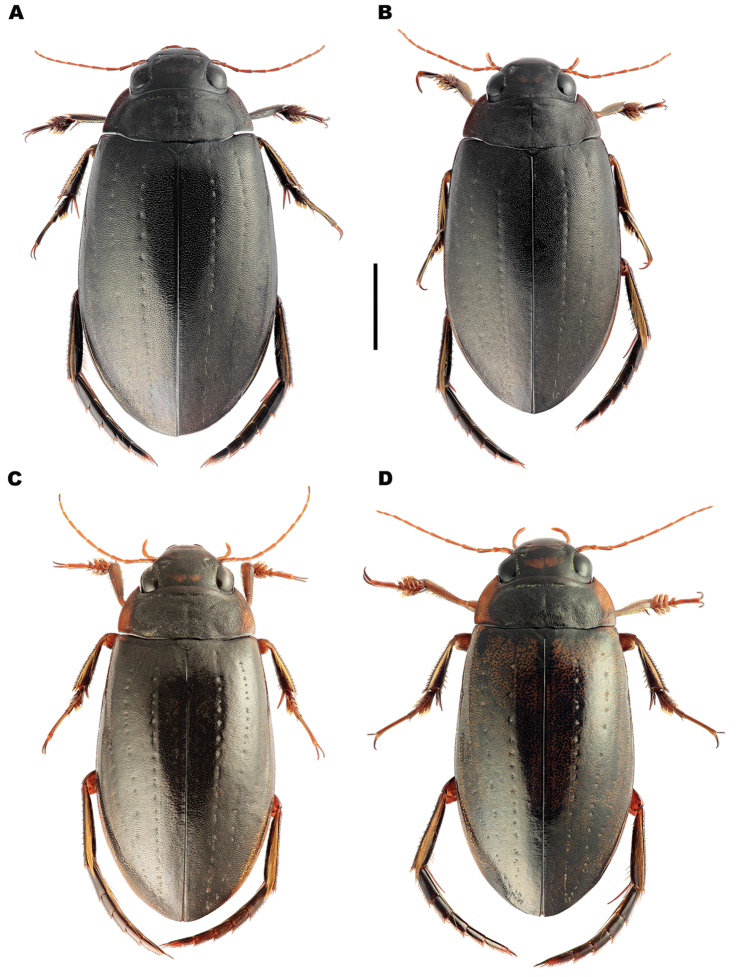
Dorsal habitus of *Meladema* species males. **A** *M. coriacea*, Spain, Cáceres, nr. Plasencia **B** *M. lepidoptera* sp. n., Corsica, Francardo **C** *M. imbricata*, La Gomera, El Cedro **D** *M. lanio*, Madeira, Rabacal. Scale bar = 5 mm.

**Figure 4.**
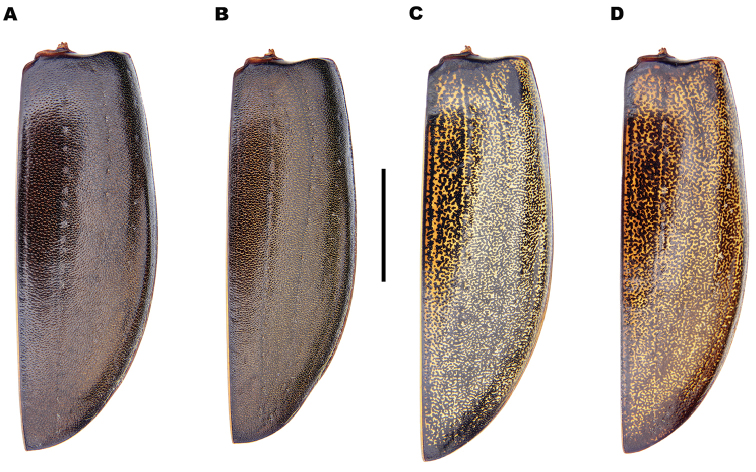
*Meladema* species males, colour pattern of isolated elytra (DNA voucher codes, where applicable). **A** *M. coriacea*, Spain, Murcia, Fte. Caputa **B** *M. lepidoptera* sp. n., Corsica, Cap Corse (NHM-IRM12F) **C** *M. imbricata*, La Gomera, El Cedro (NHM-IRM3A) **D** *M. lanio*, Madeira, Ribeira dos Cedros (NHM-IRM8A). Scale bar = 5 mm.

**Figure 5.**
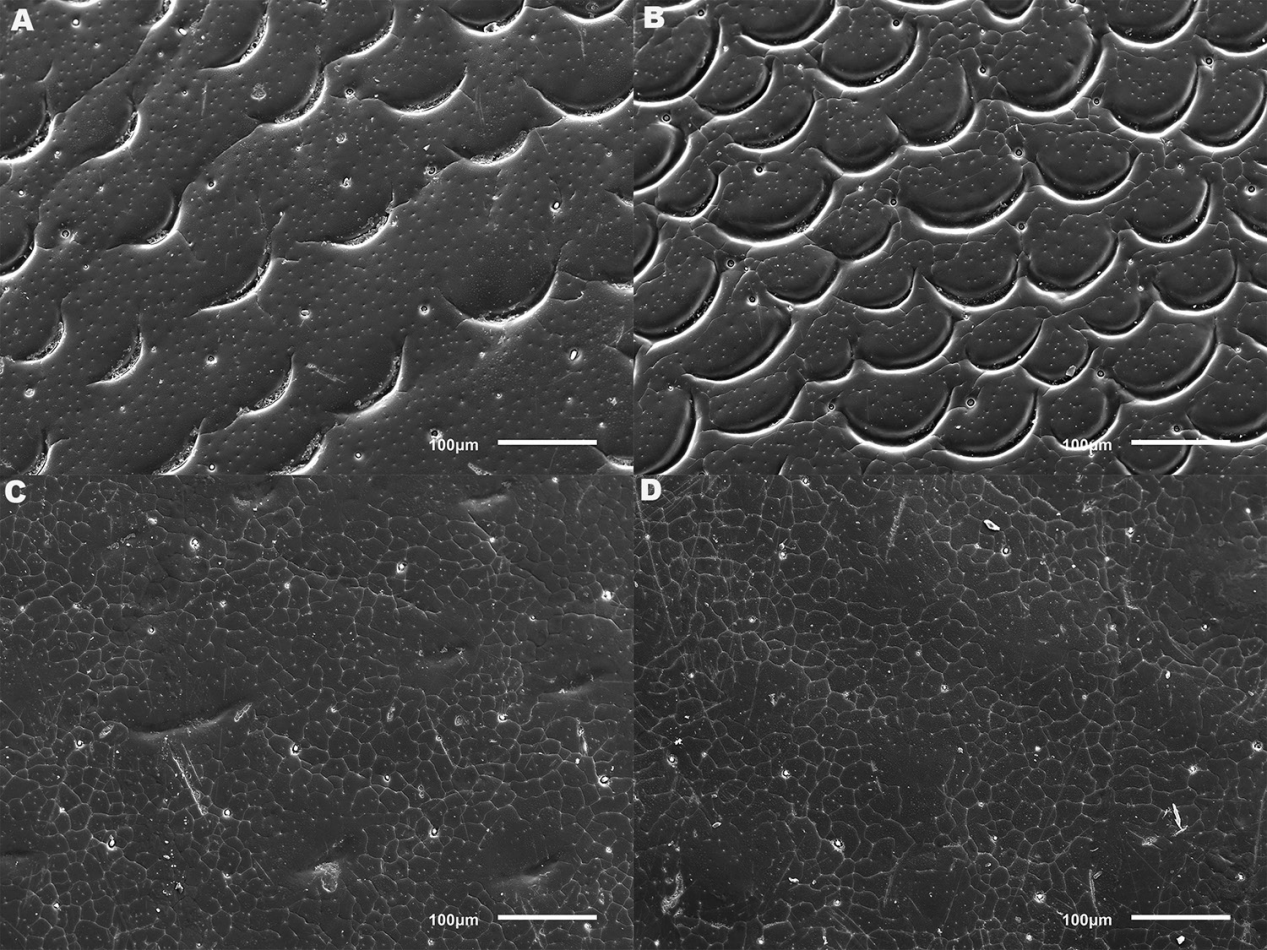
*Meladema* species males, elytral shoulder sculpture SEMs (DNA voucher codes, where applicable). **A** *M. coricaea*, Spain, Murcia, Fte. Caputa **B** *M. lepidoptera* sp. n., Corsica, Cap Corse (NHM-IRM12F) **C** *M. imbricata*, La Gomera, El Cedro (NHM-IRM3A) **D** *M. lanio*, Madeira, Ribeira dos Cedros (NHM-IRM8A).

**Figure 6.**
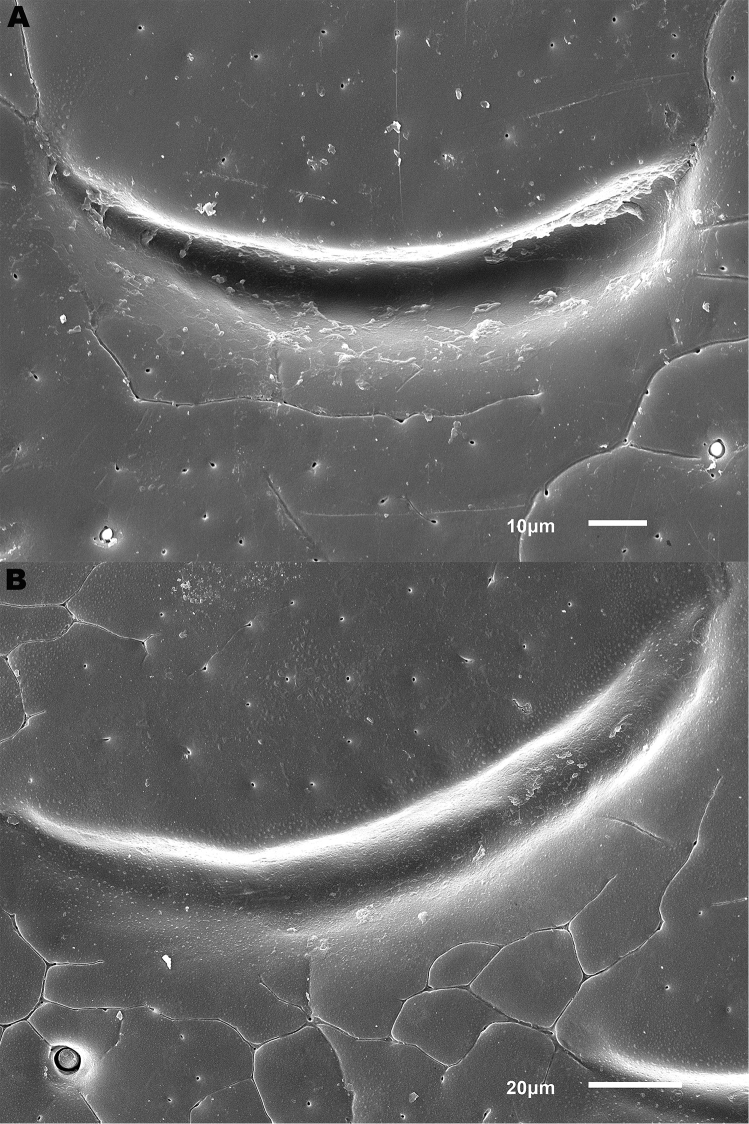
Details of elytral shoulder sculpture SEMs (DNA voucher codes where applicable). **A** *M. coriacea*, Spain, Murcia, Fte. Caputa **B** *M. lepidoptera* sp. n., Corsica, Cap Corse (NHM-IRM12F).

Compound eyes large, rounded, laterally somewhat protruding (Figure 3); anterior margin distinctly emarginate. Antennae long, slender, all segments elongate; segment 2 least so, 2.5× longer than broad; all other segments > 3× longer than broad; all segments broadening distally. Antennal insertions invisible dorsally, adjacent to anterior margins of compound eyes, below lateral margins of frons. Anterior margin of clypeus arcuate; anterior angles obtuse, weakly rounded. Clypeus with strongly transverse anterolateral foveae, each occupying approximately 0.25× length of anterior margin. Foveae shallow posteriorly, deep and abrupt anteriorly; furnished with stiff whitish or golden-yellow setae. Anterior margin of labrum with broad, semicircular emargination; central 0.4 furnished with stiff, close-set setae. Labium transverse, broadest anteriorly; anterior margin with brush of fine, close, elongate setae; lateral setae approximately 2× length of those at centre. Labial palpomeres elongate, particularly palpomere 2. Palpomere 3 with raised medial and apical tubercles ventrally, furnished with stout setae. Palpomere 3 expanded to apex, curved, convex ventrally and concave dorsally. Maxillary palpomere 1 slightly elongate; palpomeres 2–4 increasingly so. Palpomere 4 swollen, inner face almost straight, outer strongly curved; with setae on internal and ventral faces towards apex. Mentum setose anterolaterally; excavated in centre, with longitudinal ridges laterally. Pronotum strongly transverse, somewhat thickened laterally; without distinct lateral bead (Figure 3). Anterior angles acute, furnished with bunch of stiff, golden setae; posterior angles obtusely rounded. Anterior margin broadly arcuate around centre; posterior margin sinuate laterally or almost straight (Figure 3). Elytra elongate, broadest close to or behind middle (Figures 3, 4), with strong lateral bead; apices conjointly rounded. Each elytron with three rows of serial punctures (Figure 3). Each puncture of rows bearing 1–5 stout, recumbent to erect setae (e.g. Figures 3, 9, 10). Elytra of at least some individuals of all species with transverse, crescentic striolae (e.g. Figures 5, 6, 25, 26). Prosternum arched to tectiform; prosternal process lanceolate, bordered laterally, apex acuminately rounded. Metaventrite projecting anteriorly between mesocoxae; projection with elongate median groove to receive prosternal process; groove with marked central ridge, widening anteriorly. Discrimen and metacoxal suture strong, deep. Metacoxal lines strongly marked; metacoxal processes broadly rounded. Abdominal pleurite 2 without transverse ridges. Abdominal ventrites 2–6 with semicircular foveae laterally. Abdominal ventrite 6 with curved lateral wrinkles (Figures 11, 15).

*Male*. Foretarsi (Figure 12) with segments 1–3 strongly expanded and transverse, bearing four distinct rows of large articulo-setae ventrally; two on segment 1, one each on segments 2–3. Articulo-setal field bordered by dense, stiff, elongate, curved, hooked, golden setae. Segment 4 transverse; approximately 0.6–0.8 width of segment 3; with fields of stout, elongate setae of varying size laterally, inner setae curved anteriorly. Segment 5 elongate, with stout, elongate spines of varying size ventrally, close to lateral margins; basal spines curved interiorly. Foretarsal claws (Figures 12, 13) elongate, subequal, curved. Mesotarsi (Figure 12) with segments 1–3 expanded, transverse, bearing four distinct rows of large articulo-setae ventrally; two on segment 1, one each on segments 2–3. Articulo-setal field bordered by dense, stiff, elongate, curved, hooked, golden setae. Segment 4 elongate, with two rows of short, stout, stiff curved spines. Segment 5 strongly elongate, with two rows of stout spines ventrally, close to lateral margins. Mesotarsal claws elongate, subequal, curved. Abdominal ventrite 6 with weakly emarginated apex (Figure 11). Median lobe of aedeagus (Figure 14) elongate, strongly curved dorsally, with narrowly acuminate apex; lateral margins with transverse wrinkles in basal half; sinuate laterally towards apex in ventral view. Parameres (Figure 14) elongate, with dense setal fringe along internal margin, continued around apex, with scattered setae along external margin.

*Female*. Fore and mesotarsi simple, with ambulatory spines and setae only. Abdominal ventrite 6 (Figure 15) with bluntly pointed apex. Reproductive tract (Figure 16) with large, sclerotised bursa, with transverse corrugations and an elongate bursal gland; gland reddish in fresh material. Spermatheca elongate, fertilization and spermathecal ducts closely aligned, both relatively short. Gonocoxae (Figure 16) stout, elongate, with dense setation over entire surface and distinct apical penicil of setae. Laterotergites (Figure 16) elongate, with medial articulation. Gonocoxosternites (Figure 16) with bluntly pointed, setose apices. Female tract and genital structures do not appear to differ significantly between species and are not discussed further.

#### Remarks.

Both molecular and morphological data suggest a close relationship between *Meladema* and the Nearctic *Hoperius* Fall, 1927 and *Neoscutopterus* J. Balfour-Browne, 1943 (Morinière et al. 2015, 2016, Miller and Bergsten 2014). Female genital tract anatomy, here described for the first time, is similar to that described and figured for *Neoscutopterus* (Miller 2001).

### Meladema coriacea

Taxon classificationAnimaliaColeopteraDytiscidae

Laporte, 1835

Figures 3A , 4A , 5A , 6A , 7A, C , 8A, C , 9 , 10 , 11A , 12A, E 13A, 14A, 15A, 16, 17, 18D, 19, 20A–N, 23, 25C

Scutopterus coriaceus Dejean, 1833: 54, nomen nudum.
Meladema coriacea Laporte, 1835: 98 (partim); Sharp 1882: 631 (partim); Guignot 1932: 655 (partim); Gschwendtner 1936: 41 (partim); Guignot 1961: 769 (partim); Guéorguiev 1987: 127 (partim); Machado 1987: 60 (partim); Franciscolo 1979: 617 (partim);
Colymbetes coriaceus (Laporte, 1835): Aubé 1836: 94; Aubé 1838: 220 (partim); Wollaston 1865: 67 (partim).
Scutopterus coriaceus (Laporte, 1835): Wollaston 1871: 221 (partim).
Meladema coriaceum Laporte, 1835: Branden 1885: 95 (partim).

#### Notes.

Of these earlier works, only Aubé (1836) provides sufficient detail to demonstrate that he is referring to *M. coriacea* as redefined below. His description states: “entièrement couvertes de petites impressions demi-circulaires, plus profondes à la partie convexe; en avant elles sont assez bien isolées; en arrière elles sont un peu confondues”, details which allow the separation of *M. coriacea* from *M. lepidoptera* sp. n. (see below).

Laporte’s original description (1835) could refer to either *M. coricaea* as redefined here, or *M. lepidoptera* sp. n., the only reference to the unique elytral sculpture of these beetles being “corps couvert de points très serrés, presque chagriné”. As discussed by Evenhuis (2012), attempts to locate types of material described by Laporte between 1828 and 1840 have almost always proved fruitless, and it is generally assumed that this material is lost or destroyed. It seems Laporte donated his early collection (pre-1840 material) to the forerunner of the Smithsonian Institution in January 1842, and that these collections have been lost in a subsequent fire (Evenhuis 2012). As with other Laporte taxa, our attempts to locate the original type series of *M coriacea* have failed, no specimens being present in MNHN, despite Guignot (1961) suggesting that they may be located in this institution, nor in the BMNH or Melbourne Museum, Australia (S. Hinckley *pers. comm.*), the other two locations known to house some Laporte types (Sharp 1901, Horn et al. 1990). As a consequence, and in accordance with article 75.3 of the ICZN (1999), we here designate a neotype for *M. coriacea*, in order to establish its taxonomic identity and precise usage of the name. Laporte’s original description cites “Midi de la France”, a term used to describe a large part of the south of the country (not the centre, as suggested by Nilsson and Hájek, 2017a). All material we have genotyped, or examined, from southern France to date belongs to this same taxon.

#### Type locality.

“Midi de la France”.

#### Type material.

Neotype ♀ (herein designated): “24/viii/2006 FRANCE Var// La Londe-les-Maures,// Vallon de Valcros, Les// Gaouby (ruines), pools in// Maravenne Torrent, 45m// P. Ponel leg.” “43°09'45.74"N 9°15'38.82"E” “DNA Voucher// NHM-IRM11C” “*Meladema coriacea*// Laporte, 1835// NEOTYPE// D T Bilton & I Ribera des. 2017” (NMW). Dry card mounted, tissue sample in ethanol and DNA aliquote, both with same data, retained in IBE. Sequence data from the neotype has been deposited in GenBank with accession numbers AF428207 (COI-3’) and AF428189 (16S ribosomal RNA).

#### Additional material examined (genotyped specimens).

**Algeria**: 1 ♂ “24/viii/2006 ALGERIA// Aïn Damous 36 25.350N// 07 51.367E 523m V67// S. Bouzid leg.” “Meladema// coriacea Laporte// Fery det. 2007” “DNA voucher// MNCN-HI6” (IBE); 1 ♀ “24/iii/2006 ALGERIA// Oued Bagrat V28// S. Bouzid leg.” “Meladema// coriacea Laporte// Fery det. 2007” “DNA voucher// MNCN-HI4” (IBE). **Chad**: 1 ♀ “KOUDOU// VERS 2000 m.// 17 XI 49” “TIBESTI// MASSIF KOUSSI// PH. DE MIRÉ” “MUSÉUM PARIS” [blue label] “DNA voucher// IBE-AN739” (MNHN). **France, mainland**: 1 ♀ “24/viii/2006 FRANCE Var// La Londe-les-Maures,// Vallon de Valcros, Les// Gaouby (ruines), pools in// Maravenne Torrent, 45m// P. Ponel leg.” “Meladema coriaceum// FRANCE, Dept. du Var.// La Londe-les-Maures,// Vallon de Valcros,// Les Gaouby (ruines),// pools in Maravenne// torrent, alto 45 m// 5 ix 99 P. Ponel” [HW] “DNA Voucher// NHM-IRM11A” (IBE); 1 ♀ “24/viii/2006 FRANCE Var// La Londe-les-Maures,// Vallon de Valcros, Les// Gaouby (ruines), pools in// Maravenne Torrent, 45m// P. Ponel leg.” “DNA Voucher// NHM-IRM11B” (IBE). **Italy, Sicily**: 1 ♂ “SICILIA – PA// Bosco Ficuzzo// im beraio// 29.VII.88 le. M. ROMANO” [im beraio & date HW] “DNA voucher// IBE-AN691” (CTP). **Malta**: 1 ♀ “23/iv/2013 MALTA// between Mosta and L-Imtarfa// stony stream 35 53 60N// 14 24 14E A. Rudoy leg.” “DNA voucher IBE-RA1064” (IBE). **Morocco**: 1 ♂ “17/vii/1997 MOROCCO// Tazzeka Nat. Park/ 4, ca.// 4 km tras desvío Cedral// I. Ribera leg.” “DNA voucher// NHM-IRM1A” (IBE); 1 ♂ “17/vii/1997 MOROCCO// Tazzeka Nat. Park/ 4, ca.// 4 km tras desvío Cedral// I. Ribera leg.” “DNA voucher// NHM-IRM1C” (IBE); 1 ♂ “2i/vii/1997 MOROCCO// Anti-Atlas, Oued Massa// I. Ribera leg.” “DNA voucher// NHM-IRM2A” (IBE); 1 ♂ “2i/vii/1997 MOROCCO// Anti-Atlas, Oued Massa// I. Ribera leg.” “DNA voucher// NHM-IRM2B” (IBE); 1 ex. “4 MOROCCO Taza 22.3.2008// mountain stream in Tazzeka N.P.// 1448m N34°03'09.2" W4°10'27.0"// Ribera, Hernando & Aguilera leg.” “DNA Voucher// NHM-IR47” (IBE); 1 ex. “58 MOROCCO 19.4.2001// 30°47'507"N 7°31'351"W// Tachokchte: Assif Siroua// c.1500 m I.Ribera & A.Cieslak leg.” “DNA Voucher// NHM-IRM22a” (IBE); 1 ex. “58 MOROCCO 19.4.2001// 30°47'507"N 7°31'351"W// Tachokchte: Assif Siroua// c.1500 m I.Ribera & A.Cieslak leg.” “DNA Voucher// NHM-IRM22b” (IBE); 1 ex. “60 MOROCCO 21.4.2001// 30°39'456"N 9°21'134"W// Immouzèr-des-Ida-Outanane// Assif Tanit, 550 m I. Ribera & A. Cieslak” “DNA Voucher// NHM-IRM21a” (IBE); 1 ex. “60 MOROCCO 21.4.2001// 30°39'456"N 9°21'134"W// Immouzèr-des-Ida-Outanane// Assif Tanit, 550 m I. Ribera & A. Cieslak” “DNA Voucher// NHM-IRM21b” (IBE). **Spain, mainland**: 1 ♂, “1 ES Girona, Port Bou// Cami de la Riera, 28.6.2013// 42°25'34.5"N 3°8'9.3"E// 65m I.Ribera & A.Cieslak leg.” “DNA Voucher// IBE-DV293” (IBE); 1 ex. “Tarragona// Corbera d’Ebre// r. Gaia 10.1.2004 I.Ribera” “DNA Voucher// MNCN-AI104”(IBE); 1 ♂, “3 ES Huesca 6km S Bernués// Bco. de Bernués 6.9.2013// 42°26'38"N 0°36'51"W// 690m I.Ribera & A.Cieslak leg” “DNA Voucher// IBE-DV292” (IBE); 1 ex. “1 ES Castellón, Ballestar// r. Sénia, pools upstr. reservoir// 500m N40°41'41" E0°13'25.5"// I. Ribera leg. 2.6.2006” “DNA Voucher// MNCN-AI860” (IBE); 1 ex. “1 ES Castellón, Ballestar// r. Sénia, pools upstr. reservoir// 500m N40°41'41" E0°13'25.5"// I. Ribera leg. 2.6.2006” “DNA Voucher// MNCN-AI861” (IBE); 1 ♂ “8 ES Cáceres, PN Monfragüe// Arroyo de Trasierra, G. del Fraile// N39°50'07.2" W6°06'02.6" 317m// I.Ribera & P.Abellán leg. 13.6.2008” “DNA Voucher// IBE-DV291” (IBE); 1 ♂ “19/ix/1999 SPAIN Murcia// Fte. Caputa, Rio Mula// A. Millán leg.” “DNA voucher NHM-IRM13a” (IBE); 1 ♂ “29/ix/1999 SPAIN Cordoba// Baena, Arroyo de las Beatas// 36 37 00N 04 20 00W// M. Baena leg.” “DNA voucher// NHM-IRM14A” (IBE); 1 ♀ “29/ix/1999 SPAIN Cordoba// Baena, Arroyo de las Beatas// 36 37 00N 04 20 00W// M. Baena leg.” “CColeopteraBA 9.99// M. BAENA” “DNA voucher// NHM-IRM14B” (IBE); 1 ♂ “29/ix/1999 SPAIN Cordoba// Baena, Arroyo de las Beatas// 36 37 00N 04 20 00W// M. Baena leg.” “DNA voucher// NHM-IRM14C” (IBE); 1 ♀ “26/vii/1998 SPAIN Cadiz// Facinas// I. Ribera leg.” “CADIZ 98// 10a” [HW] “DNA voucher// NHM-IRM10A” (IBE). **Spain, Mallorca**: 1 ex. “4 MALLORCA Mortixet// Rd. C710 tributary Te. Son March// I.Ribera & A.Cieslak 12.11.2000” DNA Voucher// NHM-IRM23a” (IBE); 1 ex. “4 MALLORCA Mortixet// Rd. C710 tributary Te. Son March// I.Ribera & A.Cieslak 12.11.2000” DNA Voucher// NHM-IRM23b” (IBE); 1 ex. “5 MALLORCA Els Casals// Rd. C710 Te. Son March// I.Ribera & A.Cieslak 12.11.2000” “DNA Voucher// NHM-IRM24a” (IBE). **Spain, Canary Islands**: 1 ex. “2 Gran Canaria 14.4.2001// S. Nicolas de Tolentino// Risco: bco. Guy Guy grande// I.Ribera & A.Cieslak leg.” “DNA Voucher// NHM-IRM20a” (IBE); 1 ♀ “12/i/2000 Tenerife// Bco. Infierno 900m// coriacea” “DNA voucher// NHM-IRM18a” (CBP); 1 ♂ “14/i/2000 SPAIN Tenerife// Bco. de Masca// D. T. Bilton leg.” “DNA voucher// NHM-IRM19A” (CBP); 1 ♂ “14/i/2000 SPAIN Tenerife// Bco. de Masca// D. T. Bilton leg.” “DNA voucher// NHM-IRM19B” (CBP); 1 ex. “Tenerife, Chamorga// Bco. Roque Bermejo// 20.7.2006 A. Castro” “DNA Voucher// MNCN-AI1095” (IBE). **Tunisia**: 1 ♀ “13/iii/2005 TUNISIA// Cite el Morjne// 36 56 17.1N 8 47 29.8E// A. Castro leg.” “Cite-el-Morjene// N36°56'17.1// E8°47'29.8// 13-03-2005” reverse “Meladema” [HW] “MNCN AI-84// DNA spare ex.” [HW] “DNA voucher// MNCN-AI84” (IBE). **Turkey**: 1 ♀ “26/vii/2014 TURKEY Izmir// 6 km E of Phoca, head of// reservoir 38 39 37.3N// 26 49 23.9E 40m// I. Ribera & A Cieslak leg.” “TR 15” [HW] “DNA voucher// IBE-DV294” (IBE). All with “*Meladema*// *coriacea* Laporte, 1835// D T Bilton [or I Ribera] det. 2017”.

**Additional material examined (non-genotyped specimens). Algeria**: 8 ♂♂, 15 ♀♀ “Algerie// Yakouren// J Dayren// VI.VII. 1909” “MUSÉUM PARIS// 1952// COLL R OBERTHUR” [blue label] (MNHN); 1 ♂, 1 ♀ “Algerie// Yakouren// J Dayren// VI.VII. 1909” “MUSÉUM PARIS// 1952// COLL R OBERTHUR” [blue label] “Meladema// coriaceum// Cast.// de. M. Brancucci ‘82” [Latin name, describer & ’82 HW] (MNHN); 1 ♀ “Algerie// Yakouren// J Dayren// VI.VII. 1909” “MUSÉUM PARIS// 1952// COLL R OBERTHUR” [blue label] “Meladema// coriacea// Cast.” [HW] (MNHN); 1 ♀ “Meladema// coriaceum// O. Kaïrous, Mouz.// 8 avríl 06” [HW] “MUSÉUM PARIS// Coll. P. de Peyerimhoff// 1950” (MNHN); 1 ♀ “O. Kaïrous// mouzaïa// 8 avríl 06” [HW] “MUSÉUM PARIS// Coll. P. de Peyerimhoff// 1950” (MNHN); 1 ♂ “Meladema// coriaceum// Djurdj. 9” [HW] “MUSÉUM PARIS// Coll. P. de Peyerimhoff// 1950” (MNHN); 1 ♂ “Djurdjura// 14.VIII.1947” [HW] “MUSÉUM PARIS// Coll. P. de Peyerimhoff// 1950” (MNHN); 1 ♀ “Aïn Takrarat// Mouzaïa// 28.VII.1934” [HW] “MUSÉUM PARIS// Coll. P. de Peyerimhoff// 1950” (MNHN); 1 ♂ “Oued In Dalay// Kouddu// Hoggar// 19.3.1925” [HW] “MUSÉUM PARIS// Coll. P. de Peyerimhoff// 1950” (MNHN); 1 ♂, 1 ♀ “Aguelmane// Sekkarasen// Hoggar 206// 29.3.1928” [HW] “MISSION DU// HOGGAR// FÉVRIER-MAI// 1928” “MUSÉUM PARIS// Coll. P. de Peyerimhoff// 1950” (MNHN); 1 ♂ “Oued Tinikert// Mt. Oudan, 1160// 16 avril 1928” [HW] “MISSION DU// HOGGAR// FÉVRIER-MAI// 1928” “MUSÉUM PARIS// Coll. P. de Peyerimhoff// 1950” (MNHN); 1 ♀ “Tassili des Ajjers// Gta. de Tikkal// 24 mars 1961” [HW] “MUSÉUM PARIS// Coll. P. de Peyerimhoff// 1950” (MNHN); 1 ♂ [green circle, illegible HW] “Meladema// coriaceum// Bône” [HW] (ISNB); 1 ♂ “C. coriaceus// Alger” [HW] “G. C. Champion Coll.// B. M. 1927-409” (BMNH); 1 ♂ “27// 95” “Setif.// Algerien” [HW] “Meladema// coriacea Lap.// det. H. Shaverdo 2015” [Latin name, describer & 15 HW] (NMW). **Chad**: 1 ♂ “KOUDOU// VERS 2000 m.// 17 XI 49” “TIBESTI// MASSIF KOUSSI// PH. DE MIRÉ” “Meladema// coriaceum Cast.// C. Legros det.” [Latin name & describer HW] (MNHN); 2 ♀♀ “KOUDOU// VERS 2000 m.// 17 XI 49” “TIBESTI// MASSIF KOUSSI// PH. DE MIRÉ” (MNHN); 1 ♀ “Bassin de Gorrom// 2500m.// 24 déc. 1958” “TIBESTI// Emi Koussi// Bruneau de Miré” “DNA voucher// IBE-AN740” [not possible to amplify any DNA sequences] (MNHN); 1 ♀ “Tibesti// S. O du Kohoz// 2000m// 6.XI.49 Miré” [HW] “MUSÉUM PARIS// Coll. P. de Peyerimhoff// 1950” (MNHN). **France, mainland**: 1 ♂, 1 ♀ “S. France,// Cerbere.// C. Thomas &// R. L. Pocock.// 1900-215.// 18.IV.00” [date HW] (BMNH); 1 ♂ “S. France,// Cerbere.// C. Thomas &// R. L. Pocock.// 1900-215.// 18.IV.00” [date HW] “Scutopterus// coriaceus Aub,” [HW] (BMNH); 1 ♀ “S. France,// Cerbere.// C. Thomas &// R. L. Pocock.// 1900-215.// 18.IV.00” [date HW] “Meladema// coriacea Cast.// C. R. Smith det. 1982” [Latin name, describer & 2 HW] (BMNH); 1 ♂, 3 ♀♀ “ BANYULS// PYR. OR.// 12.8.1950” [12.8. HW] “BACHUFER” (NMW); 2 ♂♂, 1 ♀ “Banyuls s. Mer// 17.25 – V – 1951// F. G. Overlaet” “R, Mouchamps det.// Meladema coriacea Cast.” [Latin name & describer HW] (ISNB); 1 ♂, 2 ♀♀ “Banyuls s. Mer// 17.25 – V – 1951// F. G. Overlaet” (ISNB); 1 ♂, 1 ♀ “21.8.84 France// Pyr. Or., ung. Banyuls// Bach Fery leg.” [HW] “Mel.// coriacea// Cast. Fery det.” [HW] (NMW); 1 ♂ “meladema// coriaceum” [HW] “Foix” [HW] “Coll. E. Dongé// Le Moult vendit” (ISNB); 1 ♂ “AVEYRON FR.// ST. ROME CERN.// VII-VIII 1940” “Coll. R. Van Dorsselaer” (ISNB); 1 ♀ “Provence” “Coll. P. Boppe// Le Moult vendit” (ISNB); 3 ♀♀ “France. 10.10.55.// Provence.// leg. Weygoldt.’’ reverse “Coriaceum// Budberg” [HW] (NMW); 1 ♂ “Carcassonne// L. GAVOY” “♂” “MELADEMA// CORIACEUM// LAP.” (ISNB); 1 ♀ “Carcassonne// Aude” [HW] “Ex. Coll. Bettinger” (ISNB); 1 ♀ “Toulon.” [HW] “Gallia// mer.” “Collectio// Kaufmann” (NMW); 1 ♂, 2 ♀♀ “Toulon// S. Frankreich” reverse “A// 7598” [HW] (NMW); 1 ♂, 1 ♀ “Gard” [HW] “ex coll R P David// ex coll Peres Jesuites// (le moult vendit)” (ISNB); 1 ♂ “La Sauzette” “Meladema// coriaceum” [HW] (ISNB); 1 ♂ “St. Etienne Valléefranc. de// s// Rogerie” [HW – Villefranche de Rougerie?] “Coll. A. Fauvel” “Coriacea// Lap.” [HW] (ISNB); 1 ♂ “CAUX// HERAULT// IX 1928” [IX & 8 HW] “Collection// Dr. Guignot” “♂” “Guignot det// MELADEMA// coriacea Cast.” [Latin name & describer HW] (ISNB); 1 ♂ “CAUX// HERAULT// IX 1928” [8 HW] “♂” “MUSÉUM PARIS// 1960// Coll F. Guignot” “coriacea Cast.” [HW] (MNHN); 2 ♀♀ “CAUX// HERAULT// IX 1928” [8 HW] “♀” “MUSÉUM PARIS// 1960// Coll F. Guignot” (MNHN); 2 ♂♂ “neffies// HERAULT// H. LAVAGNE” [neffies HW] “Coriaceum” [HW] “Ex Coll. Bettinger” (ISNB); 1 ♀ “St GUILHEM// HERAULT// H. LAVAGNE” “Coriaceum” [HW] “Ex. Coll. Bettinger” (ISNB); 1 ♀ “Meladema// coriaceum” [HW] “Montpellier// (Hérault)” [HW] “Coll. E. Dongé// Le Moult vendit” (ISNB); 1 ♀ “Béziers// (He^lt^.)” [HW] “Coll. E. Dongé// Le Moult vendit” (ISNB); 2 ♂♂ “Lozère” [HW] “Meladema// coriaceum” [HW] “Coll. E. Dongé// Le Moult vendit” (ISNB); 2 ♂♂ “Lozère” [small, circular brown label, no text] “Coll. Odier.// B. M. 1921-288” (BMNH); 1 ♀ “♀// 5188” [HW] “Coll. R. I. Sc. N. B.// France Marseille// Coll. Fairmaire// Coll. L. Pandellé” [Marseille & Coll. Fairmaire HW] “Coll. A. Fauvel// (ex. coll. Pandellé)// Meladema// coriacea Lap.// R.I.Sc.N.B. 17.819” [Latin name & describer HW] (ISNB); 1 ♀ “♀” [HW] “Coll. R. I. Sc. N. B.// France Marseille// Coll. Fairmaire// Coll. L. Pandellé” [Marseille & Coll. Fairmaire HW] “Coll. A. Fauvel// (ex. coll. Pandellé)// Meladema// coriacea Lap.// R.I.Sc.N.B. 17.819” [Latin name & describer HW] (ISNB); 1 ♂ “♂// 5188” [HW] “Coll. R. I. Sc. N. B.// France Marseille// Coll. Fairmaire// Coll. L. Pandellé” [Marseille & Coll. Fairmaire HW] “Coll. A. Fauvel// (ex. coll. Pandellé)// Meladema// coriacea Lap.// R.I.Sc.N.B. 17.819” [Latin name & describer HW] (ISNB); 1 ♂ “♂// 5188” [HW] “Coll. R. I. Sc. N. B.// France Marseille// Coll. Fairmaire// Coll. L. Pandellé” [Marseille & Coll. Fairmaire HW] “Coll. A. Fauvel// (ex. coll. Pandellé)// Meladema// coriacea Lap.// R.I.Sc.N.B. 17.819” [Latin name & describer HW] “Colymbetes Clairville// coriaceus, Hoffm.// Marseilles” [HW] (ISNB); 6 ♂♂, 2 ♀♀ “St Cyr// Ravindu// Degoutant” [HW] (MNHN); 1 ♀ “Le Beausset.// Octobre” “Meladema// coriaceum Lap.” [HW] (NMW); 1 ♀ “Le Beausset.// Mol. de Boissy” (NMW); 1 ♀ “M. coriaceum” [HW] “M. de Boissy// Le Beausset Var” [HW] (NMW); 1 ♀ “Rhonetal// mont. ol. Maures// östl. Aixen Provence” [HW] “M. J. MAURES// Det. VI/81//511” [HW] (NMW); 1 ♂, 1 ♀ “Nice” [HW] “coll. P. J. Roeflofs” (ISNB); 1 ♀ “Gallia merid// Agay (Var)// 15 Juli 14// W. Liebmann// Arnstadt” [15 HW] (NMW); 2 ♂♂ “Gallia merid// Agay (Var)// 15 Juli 14// W. Liebmann// Arnstadt” [15 HW] “Collectio// Paganetti” (NMW); 1 ♂, 1 ♀ “St. Maxime// Var// G. Audran” [HW] “R. Mus. Hist. Nat.// Belg. I.G. 14.406” (ISNB); 1 ♀ “Hyères” [HW] “coll. A. Fauvel” (ISNB); 1 ♂ “Nyons” [HW] “Colymbetes// coriaceus// 22” [HW] “coll. Delgrange” (ISNB); 1 ♀ “Colymbetes// coriaceus// Capt. P. Bauret” [HW] “Trinité-Victor// Juillet 1942” [HW] (ISNB); 1 ♂ “Colymbetes// coriaceus// Lap.” [HW] “Oct. 46// Le Laghet// Trinite Victor” [HW] (ISNB); 1 ♀ “Colymbetes// coriaceus// Lap.” [HW] “October 1945// Le Laghet// Trinité Victor” [HW] (ISNB); 1 ♂ “Cavalaire” [HW] “f. 532 Coll.//Dr. Mouchamps” [32 HW] “Pavel Riha leg.// MELADEMA// coriacea Cast.” [Latin name & describer HW] (ISNB); 3 ♀♀ “261// 131” [blue, circular label, HW] reverse “Savoy” [HW] (BMNH); 1 ♂ “[illegible]// Coll. Chevrolat// Det. Sharp 82.” [HW] “3306” “Colymbetes// coriaceus// Ex Aubé 220,// Gall. mer.” [HW] (ISNB); 1 ♂ “Gallia m.” (NMW); 1 ♂ “8367” [blue-green label, HW] “Gallia” [HW] “Fry Coll.// 1905-100” (BMNH); 1 ♀ “Gallia” [HW] “Fry Coll.// 1905-100” (BMNH); 1 ♀ “Colymbetes// coriaceus. Hoffm.// Gehin France” [HW] (BMNH); 1 ♀ “Coriaceus// Gallia” [blue label, HW] “Sharp Coll.// 1905-31.” (BMNH); 1 ♀ “Gallia 10” “Coll. Liepolt” (ISNB); 1 ♂ “coriaceus Lap.// Gallia// Bien Bacher” [HW] “c. Epplsh.// Steind. d.” (NMW). **Greece, mainland**: 1 ♂ “Attica// Dr. Kinper” [HW] “Collect.// Hausser” (NMW); 1 ♀ “Attica// Dr. Kinper” [HW] “punctulatus” [HW] “Collect.// Hausser” (NMW). **Greece, Aegina**: 1 ♀ “Griechenland// Aegina// F. Werner, 13 V. 37” [Aegina & 13 HW] (NMW). **Greece, Amorgos**: 1 ♀ “Graecia, KYKL.// Amorgos// P. Velensky lgt.// 13.-16.5.1984” “Coll.// HENDRICH// Berlin” (ZSM); 1 ♀ “Graecia, KYKL.// Amorgos// 13.-16.5.1984// P. Velensky lgt.” “Coll.// HENDRICH// Berlin” (ZSM). **Greece, Chios**: 1 ♀ “GR: Chios// Mai 1980// lg. Bilak, Kritscher” (NMW). **Greece, Corfu**: 1 ♂, 1 ♀ “GREECE: NE Corfu (GR3)// ca. 1.5 km W Zigos// 200 m a.s.l., 19.VII.08// leg. M. A. Jäch” “small shallow residual// pool in river bed// 39°43'44.4"N// 19°46'38.3"E” “Meladema// coriacea Laporte// Fery det. 2010” (NMW); 1 ♂, 2 ♀♀ “28/v/2017 Greece Corfu// nr. Zigos 233 m// 39 43 40.6N 19 46 41.0E// C R Turner leg.” (CBP). **Greece, Ios**: 1 ♂ “Kykladen// Ios, Buch von// Milopotamos” “leg. Schönmann// 9. April 1981” [first 9 HW] (NMW); 1 ♀ “Kykladen// Ios, Buch von// Milopotamos” “leg. Schönmann// 8. April 1981” [first 8 HW] (NMW). **Greece, Kithira**: 1 ♀ “GR-Kithira (4)// Arlemonas 8.V.76// leg. Malicky” “Melaema// coriacea Cast.// det. Wewalka 76” [HW] (NMW). **Greece, Milos**: 3 ♂♂, 1 ♀ “GR-MILOS 19.9.90// 1km E. Sider ianos.// leg. M. Jäch (5)” (NMW). **Greece, Naxos**: 1 ♀ “GR-Naxos V.1988// leg. Bilak// et Kritscher” (NMW); 2 ♀♀ “GR-Naxos (13)// Potamia 26.V.76// leg. Malicky” (NMW). **Greece, Poros**: 1 ♂ “GR-Poros (2)// Kampos 17.V.74// leg. Malicky” (NMW). **Greece, other**: 1 ♀ “Griechenland// Kylhima// F. Werner, 26.V.37” [Kylhima & 26 HW] (NMW); 1 ♂ “Greece// Merlin Coll.// 96-275” (BMNH); 1 ♂ “Greece// Merlin Coll.// 96-275” “Spec. nov.// Hymettus” [pink label, HW] “Received with// this name// from Merlin// C. O. W.” [Merlin HW] (BMNH); 1 ♂ “Graecia” “Meladema// coriaceus” [HW] “Coll. Plason” (NMW). **Italy, mainland**: 1 ♂ “Grotta di// Pastena// Italie merid.” [HW] “Meladema// coriacea Cast.” [HW] “Brit. Mus.// 1963-344.” (BMNH); 1 ♂, 2 ♀♀ “Meladema// coriaceum” [HW] “Italie” [HW] “Coll. E. Dongé// Le Moult vendit” (ISNB); 1 ♂ “Coriaceus// Italia F” [HW] “R. Mouchamps det.// Meladema// coricaea Cast.” [Latin name & describer HW] (NMW); 1 ♀ “Meladema// coriaceum” [HW] “Calabre” [HW] “Coll. E. Dongé// Le Moult vendit” (ISNB); 1 ♀ “Gerace, Calab.// Paganetti” “Collectio// Paganetti” (NMW); 1 ♀ “Gerace, Calab.// Paganetti” “Collectio// Paganetti” “Meladema// coriaceum” [HW](NMW). **Italy, Pontine Islands**: 2 ♂♂, 3 ♀♀ “Ins. Pont. - @ nos// auf Tamanis// 12-4-57// Dr. Eckerlein leg. [HW] (NMW). **Italy, Sicily**: 1 ♂ “Madricia// Ragusa” [HW] “3306” (ISNB); 1 ♀ “Monts Madonie 74” “E. Ragusa” (BMNH); 1 ♀ “Castelbueno// Ragusa” [HW] “3307” (ISNB); 2 ♂♂, 1 ♀ “Sicil.” “Collectio// Kaufmann” (NMW); 1 ♀ [circular label, black margin, no text] “Sicilia” [HW] “alte// Sammlung” (ZSM); 1 ♀ “Sic.” “Sammlung// C. Müller” (ZSM); 1 ♀ “Italia: Sicilia – PA// MTE. Maganoce// 900 M. Ü. N.” [HW] (ZSM). **Malta**: 2 ♀♀ “Prof. Barthet// Malta” “29.8.25// Malta” [HW] “R.L.S.N.B.//I.G. 17.619” (ISNB); 1 ♀ “Malta” [HW] “Ex. Coll. Bettinger” (ISNB); 1 ♂ “Malta” “G.C. Champion Coll.// B. M. 1929-409” “Meladema// coriaceum Lap.// Malta” [HW] (BMNH); 1 ♂ “Buskett Gdns.// Malta// 12.XII.55.// G. V. P. Sewell” [HW] “Meladema// coriacea Cast// C. R. Smith det. 182” [Latin name, describer & 2 HW] (BMNH); 1 ♂ “MALTA// Chadwick Lakes// Mars ‘85// D. Johnson” [HW] (NMW). **Morocco**: 1 ♂, 1 ♀ “Tachdirt// Marocco// Schwingenschuss” [HW] “Coll. Liepolt” (ISNB); 7 ♂♂, 5 ♀♀ “Maroc: Azrou// 6-12-vii-1934 (1350m)// A. Ball 56 M 22” “R. Mus. Hist. Nat.// Belg. I.G. 10.417” “A. Ball det. 1935// Meladema// coriacea Cast.” [1935, Latin name & describer HW] (ISNB); 1 ♀ “Middle Atlas Mts// Oviduane// 2,000ft.” “MOROCCO:// K. Chapman &// J.W.S. Pringle.// B.M. 1934-554” ‘MELADEMA// coriacea// Laporte” [HW] “det. J. Hajek// xi. 2008” (BMNH); 4 ♂♂, 4 ♀♀ “Middle Atlas Mts// Oviduane// 2,000ft.” “MOROCCO:// K. Chapman &// J.W.S. Pringle.// B.M. 1934-554” (BMNH); 1 ♂ “Middle Atlas Mts// Oviduane// 2,000ft.” “MOROCCO:// K. Chapman &// J.W.S. Pringle.// B.M. 1934-554” “Oum en Rhio Aug 20^th^// large beetle 400” [folded, HW, 400 in a box] (BMNH); 1 ♀ “MOROCCO// Dayet Ifrah (lake)// 16 km. E.N.E. Ifrane// 28.V.1961.” [all except Morocco HW] “1334// P. N. Lawrence// B. M. 1961-328” [HW] (BMNH); 1 ♀ “MOROCCO// Great Atlas Mts.// Ijoukak. 3,900ft.// 3.vi.1936” [3 HW] “K. H. Chapman// & G. A. Bisset.// B. M. 1936-527.” (BMNH); 1 ♀ “S. MORROCCO// TIZI-N-BACHKOUM env.// 25.4.1995// P. Prüdek leg.” “Coll.// HENDRICH// Berlin” “Meladema// coriacea// Cast.// HENDRICH det. 1996” [name & describer HW] (ZSM); 1 ♂ “Imi n’ Ouaka// 1500m.” “Maroc// 1-15 Sept.” “♂” “♂” “MUSÉUM PARIS// 1960// Coll F. Guignot” (MNHN); 2 exx. “71 MOROCCO 8.4.2007// Tizi-n’Rechou, Kerrouchèn// stream on rock N pass, rd. 3437// 1570m N32°48'22.1" W5°16'13.9"// Aguilera Hernando & Ribera leg.” (IBE); 1 ♀ “99 MOROCCO Chefchauen// 13.2.2015 afl. Oued Laou 150m// 35°17'57.0"N 5°12'55.5"W// Ribera, Millán & Velasco leg.” (IBE); 1♂, 1♀ “Morocco// Âit-Iftene// 29, Oued Ait-Baha// 220797 Ribera, Aguilera, Hernando, Millán” (IBE); 1♂, 1♀ “Morocco// Sidi-Ibrahim// 17, 77.5 km S Guercif// 150797 Ribera, Aguilera, Hernando, Millán” (IBE); 1 ex. “MOROCCO Haut Atlas// NE Tizi-n-Test, 1710m No.18// 30°54'12N 08°18'39W// 30.12.2002 V.Assing & P.Wunderle” (IBE). **Portugal, mainland**: 1 ♂ “Portugal// Oliveira” [HW] “3306” (ISNB); 3 ♀♀ “Portugal// Oliveira” [HW] “3307” (ISNB); 1 ♀ “Portugal// Oliveira” [HW] “♀” (ISNB); 1 ♂ “Portugal// Oliveira” [HW] (ISNB); 8 ♂♂, 10 ♀♀ “PORTUGAL:// Serra do Malhao.// 8.v.1966// J. Abraham &// M. E. Bacchus.// B. M. 1966-296” “M. E. Bacchus// B. M. 1966-296” (BMNH); 1 ♀ “PORTUGAL:// Algarve. Rio Seco.// 250m ca. 10km.// W. Barranco de// Velho. 10.v.1966.” (BMNH); 1 ♀ “PORTUGAL:// Algarve. Rio Seco.// 250m ca. 10km.// W. Barranco de// Velho. 10.v.1966.” “Meladema// coriacea cast.// J. Balfour-Browne det.// xii. 1972” (Latin name, describer & 72 HW) (BMNH); 1 ♀ “5/v/93 Portugal// Viana do Castelo// Serra do Minho// stream @ ca. 700m// D. T. Bilton leg.” [HW] (CBP); 1 ♀ “PORTUGAL: Traz os Montes// Carrazeda de Anciais// 745m., 7.viii.1966” “slow weedy// stream” “Brit. Mus.// 1973-562” “Meladema// coriacea Cast.// J. Balfour-Browne det.// IX 1969” [Latin name, describer, IX & last 9 HW] (BMNH). **Spain, mainland**: 1 ♂, 2 ♀♀ “Cataluna// Vall. x-42// Español” [HW] “R. Mouchamps det 1948// MELADEMA// coriacea Cast.” [8, Latin name & describer HW] (ISNB); 2 ♂♂ “Vallvidrera// Barcelona// VIII.1948// Español” [HW] “R. Mouchamps det.// Meladema// coriacea Cast.” [Latin name & describer HW] (ISNB); 2 exx. “Barcelona// Montseny d’Amunt// Riachuelo: remanso// 290988 I. Ribera” (IBE); 1 ♂ “Girona// Capmany// Estanys: alberca// 041189 I.Ribera” (IBE); 3 ♂♂, 1 ♀ “Girona// Capmany// Estanys: alberca// 110389 I.Ribera” (IBE); 4 ♂♂, 3 ♀♀ “Girona// Capmany// Estanys: alberca// 281090 I. Ribera” (IBE); 6 ♂♂, 3 ♀♀ “Girona// Capmany// Estanys: alberca// 251190 I. Ribera” (IBE); 4 ♂♂, 3 ♀♀ “Girona// Capmany// Estanys: alberca// 120191 I.Ribera” (IBE); 2 ♀ “Girona// Capmany// Estanys: alberca// 171294 I. Ribera & P. Aguilera” (IBE);1 ♂ “Girona// Capmany// Estanys: alberca// 100994 I.Ribera & P.Aguilera” (IBE); 2 exx. “1 GIR La Junquera 4.x.2014// estanys de Capmany: alberca// 42°24'20.5"N 2°54'8.0"E// 188m I. & B.Ribera leg.” (IBE); 1 ♂ “Girona// Capmany// Querafumat: abrevadero 1// 251190 I. Ribera” (IBE); 2 ♂♂, 1 ♀ “2 ES Girona, Port Bou// Cami de la Riera, 10.5.2012// N42°25'38.2" E3°08'13.4"// 81m I.Ribera & A.Cieslak leg.” (IBE); 2 ♂♂, 2 ♀♀ “Girona// Sta. Pau// Rio Ser: remanso 1b// 260890 I. Ribera” (IBE); 1 ♀ “Girona// Sta. Pau// Rio Ser: remanso 2// 260890 I. Ribera” (IBE); 2 ♂♂, 1 ♀ “1 ES Girona, Port Bou// Cami de la Riera, 28.6.2013// 42°25'34.5"N 3°8'9.3"E// 65m I.Ribera & A.Cieslak leg.” (IBE); 1 ex. “Tarragona// Vila-rodona// Jardín: alberca// 110985 M.Galán” (IBE); 1 ex. “Tarragona// Vila-rodona// Jardín: alberca// 201085 I.Ribera” (IBE); 2 ♂♂, 2 ♀♀ “16/v/1994// SPAIN Tarragona// stream 2km SW of// Esblada D. T. Bilton leg.” (CBP); 1 ♂, 3 ♀♀ “3 ES Huesca 6km S Bernués// Bco. de Bernués 6.9.2013// 42°26'38"N 0°36'51"W// 690m I.Ribera & A.Cieslak leg” (IBE); 3 exx. “1 ES Castellón, Ballestar// r. Sénia, pools upstr. reservoir// 500m N40°41'41" E0°13'25.5"// I. Ribera leg. 2.6.2006” (IBE); 1 ♂, “Teruel// Calaceite// Riu Matarranya// 240794 I.Ribera & P.Aguilera” (IBE); 1 ♂, 1 ♀ “19/ix/1999 SPAIN Murcia// Fte. Caputa, Rio Mula// A. Millán leg.” (IBE); 1 ♂ “19/ix/1999 SPAIN Murcia// Fte. Caputa, Rio Mula// A. Millán leg.” “Fte Caputa// R. Mula 19-9-99// A. Mellado” [HW] (IBE); 1 ♂ “19: SPAIN: CACERES:// 21 km S of Aldeanuo// del Camino: granite// stream 18 April 1985” (CBP); 1 ♂, 1 ♀ “12/v/1990 SPAIN Cáceres// stream with pools 7km N of// Plasencia on N630 road” (CBP); 2 ♀♀ “27/iv/1993// Spain Extremadura// Cáceres stream by N630// 15km N of Plasencia” (CBP); 1 ex. “3 ES Cáceres, PN Monfragüe// Arroyo de Malvecino// N39°51'04.0" W6°02'22.3" 284m// I.Ribera & P.Abellán leg. 12.6.2008” (IBE); 1 ♀ “SPAIN: Cordoba.// Posadas – Villaviciosa Rd.// 20 Km N. of Posadas// Arroyo Calderas. 500m.// 14.V.1967” “Meladema coriacea// CAST. I. RIBERA det 1990” [HW] (BMNH); 1 ♂ “MALAGA// H. Clark.// May 1856” “Fry Coll.// 1905-100” (BMNH); 2 ♀♀ “MALAGA// H. Clark.// May 1856” (BMNH); 1 ex. “1 ESP. Malaga 3.1.03// Villanueva del Trabuco// r. Guadalhorce, cta. MA156// I.Ribera & A.Cieslak leg.” (IBE); 1 ♂, 1 ♀ “Almería// Tabernas// Rbla. de Tabernas: charca 1// 110597 I.Ribera & A.Millán” (IBE); 1 ♂ “Nerja” [HW] (MNHN); 1 ♂ “24/iv/1993// Spain Andalucia Cadiz// Drying stream by N340// 28km N of Tarifa” (CBP); 1 ♀ “17: SPAIN: HUELVA: 1 km// S. of Santa Ollala del// Cala: weedy stream:// 18 April 1985// R.B. Angus & G.N. Foster” “Meladema corac” [HW] (BMNH); 2 ♂♂, 1 ♀ “17: SPAIN: HUELVA: 1 km// S. of Santa Ollala del// Cala: weedy stream:// 18 April 1985// R.B. Angus & G.N. Foster” “R. B. Angus// BMNH(E) 2010-22” (BMNH); 1 ♂, 1 ♀ “Hispania” [HW] “Meladema// Lap.// coriacea// Cast.” [HW, genus name on green label, glued on top of original] “Coll. Martin// Le Moult vendit” (ISNB); 1 ♂, 1 ♀ “Hispan” [HW] “Fry Coll.// 1905-100” (BMNH); 1 ♂ “Hispa// nia” [HW] “57.// 107.” [blue, circular label, HW] (BMNH). **Spain, Mallorca**: 2 exx. “1 Mallorca, Lloseta 13.5.2007// te. des Estorall, potabilizadora// 170m N39°43'49.7" E2°50'04.6"// I.Ribera & A.Cieslak leg.” (IBE); 1 ♂, 1 ♀ “2 Mallorca, Bunyola 13.5.2007// te. de Bunyola, Ma 2100 pk 5.5// 455m N39°43'24.7" E2°43'15.7"// I.Ribera & A.Cieslak leg.” (IBE) ; 1 ♂ “Inca, Majorca.// March.// O. Thomas &// R. I. Pocock.// 1900-215” (BMNH). **Spain, Canary Islands**: 2 ♀♀ “Canary Is.// Gran Canaria,// Sta Brigida.// 5.ix.1927.// E. Appenhagen.// B. M. 1928-20” [5 & ix HW] (BMNH); 1 ♂ “Canary Is.// Gran Canaria,// Sta Brigida.// 5.ix.1927.// E. Appenhagen.// B. M. 1928-20” [5 & ix HW] “Colymbetes// coriaceus, Cast.// 5-9-27.// 206” [HW] (BMNH); 1 ♀ “CANARY IS.// Gran Canaria// 29.xii.92-11.i.93” “Bco de// Ayagaures// ca 100 m.” [HW] “P. M. Hammond// B. M. 1993-11” “coriaceus” [HW] (BMNH); 1 ♂ “Barranco de// Temise près Haria// (Lanzarote) 2” [HW] “coll. A. Fauvel” (ISNB); 1 ♀ “Colymbetes// coriaceus Hoffm.// Bajamar XI. 1909” [HW] “Le Moult vend.” (ISNB); 1 ♂ “Teneriffa// geibelez” [HW] “DON// P DUPUIS” (ISNB); 1 ♀ “Colymbetes Clairv.// 203 coriaceus Lap.// 6 ex Teneriffe 1904 (Prevost):Elliott Coll.” [HW, folded] “Claude Morley// Collection// B.M. 1952-159” “Meladema// coriacea Cast.// C. R. Smith det. 1982” [Latin name, describer & 2 HW] (BMNH); 2 ♂♂, 1 ♀ “Claude Morley// Collection// B.M. 1952-159” [part of same series as previous] (BMNH); 1 ♂ “Claude Morley// Collection// B.M. 1952-159” “Meladema// coriacea Cast.// C. R. Smith det. 1982” [Latin name, describer & 2 HW] [part of same series as previous] (BMNH); 2 ♂♂, 2 ♀♀ “Canary Is.// Teneriffe,// Guajonje,// 17.iii.1927// E. Appenhagen” [Guajonje & date HW] (BMNH); 1 ♀ “Canary Is.// Teneriffe,// Guajonje,// 17.iii.1927// E. Appenhagen” [Guajonje & date HW] “Colymbetes// coriaceus, Lap.// G. J. Arrow det.” [Latin name & describer HW] (BMNH); 1 ♂ “Tenerife// Barr. Bufadno” [HW] “6.2.1949// Fernandez” [6, 2 & last 9 HW] “Brit. Mus.// 1951-318” “Meladema// coriaceum Lap.// Harald Lindb. det” [Latin name & describer HW] (BMNH); 1 ♂ “ES: Tenerife// Masca 12 April// 1991 AN Nilsson” “Meladema coriacea// Laporte, 1835// Det AN Nilsson 1991” (CBP); 1 ♂ “April 1998 SPAIN// Islas Canarias Tenerife// Bco. del Infierno Pools// D.T. Bilton leg.” (CBP); 1 ♂, 1 ♀ “April 1998 SPAIN// Islas Canarias Tenerife// Bco. de Masca Pools// D.T. Bilton leg.” (CBP); 1 ♀ “29/xi/97 Tenerife// Anaga Massif – stream// @ Iguana D.T. Bilton” [HW] (CBP); 1 ♀ “CANARIES” “Coll. R. Van Dorsselaer” (ISNB); 1 ♀ “Canary Is.// 1903 (Prevost)// coll. Elliott . 36” [HW] “Claude Morley// Collection// B.M. 1952-159” (BMNH). **Tunisia**: 3 ♀♀ “TUN.: 3.8.91(7)// Beni Melir SE// A. Draham, Schödl” (NMW). **Turkey**: 1 ♀ “Asia min. occ.// Izmir (H. Mudja)// 20 m. St. 71// 8-V-’31 (Orch.)” “Reg. Mus. Hist. Nat.// Belg. I.G. 9642” “A. Ball det. 1935// Meladema// coriacea Cast.” [1935, Latin name & describer HW] (ISNB). **Without locality data**: 1 ♂ “Coll Abcedal” [HW] “Colymbetes// coriaceus” [HW] “R.I.Sc.N.B. 21.418// Coll. P. de Moffarts” “Meladema// coriaceum// Lap.” [green label, HW] (ISNB); 1 ♂ “Coll Abcedal” [HW] “Coll. P. de Moffarts” (ISNB); 1 ♀ “Meladema// coriaceum// Coll. Hevon” [HW] “Ex coll. Bettinger” (ISNB); 1 ♂ “121” “Meladema// coriaceum” [HW] “Ex. Coll. Bettinger” (ISNB); 1 ♂ “ex coll R P David// ex coll Peres Jesuites// (Le Moult vendit)” “coriaceus Cast.// Region mediterranee” [HW] (ISNB); 1 ♂ “Coll. P. Boppe// Le Moult vendit” “Meladema// coriaceum// Cast.” [HW] (ISNB); 1 ♂ “Meladema// coriaceum” [HW] “Coll. E. Dongé// Le Moult vendit” (ISNB); 1 ♂ “H Guyon” [HW] “Coll. E. Traizet// Le Moult vendit” (ISNB); 7 ♂♂, 4 ♀♀ “coll. A. Fauvel” (ISNB); 1 ♂ “coll. Delgrange” (ISNB); 1 ♂ “G. C. Champion Coll.// B. M. 1927-409” (BMNH); 1 ♀ “Coll. Odier.// B. M. 1921-288” (BMNH); 2 ♀♀ [small, circular brown label, no text] “Coll. Odier.// B. M. 1921-288” (BMNH); 1 ♂ “Alono-// dorsar” [? HW] “Meladema// coriacea” [HW] (BMNH); 1 ♂ “Europa// B. Heyne” [HW] (BMNH); 1 ♂ “coriaceus Lap.” [folded, HW] (BMNH); 1 ♂ “Dytiscus coriaceus// no. in list. 19.” [HW] reverse “Presented by// Signor Passerini” [name HW] (BMNH); 1 ♂ “1911” [round label, HW] (BMNH). All with “*Meladema*// *coriacea* Laporte, 1835// D T Bilton [or I Ribera] det. 2017”.

#### Description.

*Size*: Neotype TL = 22.66 mm; EL = 16.90 mm; MW = 11.52 mm. Other material examined TL = 18.56–23.17 mm; EL = 14.34–16.90 mm; MW = 8.45–11.14 mm.

*Colour*. Dorsum dark reddish brown to black (Figure 3A); lateral margins of pronotum, labrum and anterior half of clypeus somewhat paler, sometimes with diffuse lateral maculae. Elytra unicolorous, without distinct mottling even when lifted (Figure 4A). Head with a pair of oval, reddish yellow medial interocular patches, slightly elongated apicolaterally. Antennae and maxillary and labial palpi reddish yellow. Legs dark reddish brown to black with golden yellow setae; large spines somewhat paler. Venter reddish brown to black; gula, meso and metacoxae and trochanters paler.

*Head*. Labrum shining, with moderate to coarse, sparse punctures. Reticulation absent in apical half, becoming increasingly more evident basally, here forming weakly impressed, transverse meshes. Clypeus and anterior half of frons shining, doubly punctate, without reticulation and with very close, fine and very sparse, coarse punctures. Coarse punctures approximately 5–8x diameter of fine; without visible reticulation. Paired epicranial foveae, one immediately behind the other, on each side of frons, close to lateral margins and immediately behind lateral remnants of frontoclypeal suture. Anterior epicranial foveae transverse, posterior slightly elongate oval; both with cluster of stout, yellow recumbent to decumbent setae. Areas between anterior and posterior foveae with coarse wrinkles. Posterior frons with open, elongate, wrinkled reticulation, especially alongside lateral margins of compound eyes and onto vertex; meshes tumid, with rugose appearance. Internal and posterior borders of compound eyes distinct, raised relative to level of adjacent cuticle. Lateral margins bordered by distinct narrow channel; deeper anteriorly than posteriorly and continuing behind posterior margin of eye onto vertex. Channel with dense punctures, bearing long, stiff, yellow recumbent to decumbent setae.

*Pronotum*. Posterior margin strongly sinuate laterally (Figure 3A). Surface somewhat shining, strongly rugose. Reticulation meshes large, open, almost isodiametric and relatively flat either side of mid-line on disc; smaller, tumid and more uneven in size and shape towards all margins. Transverse irregular row of medium punctures bearing long, yellow recumbent to decumbent setae 1/5 behind anterior pronotal margin; interrupted briefly in centre, continuing inside lateral margins and inside lateral third of posterior margin. Centre of disc with elongate, narrow, slit-like fovea, sometimes partially interrupted in mid-length. Lateral margins slightly raised, shining, without rugose sculpture and with fine, scattered punctures.

**Figure 7.**
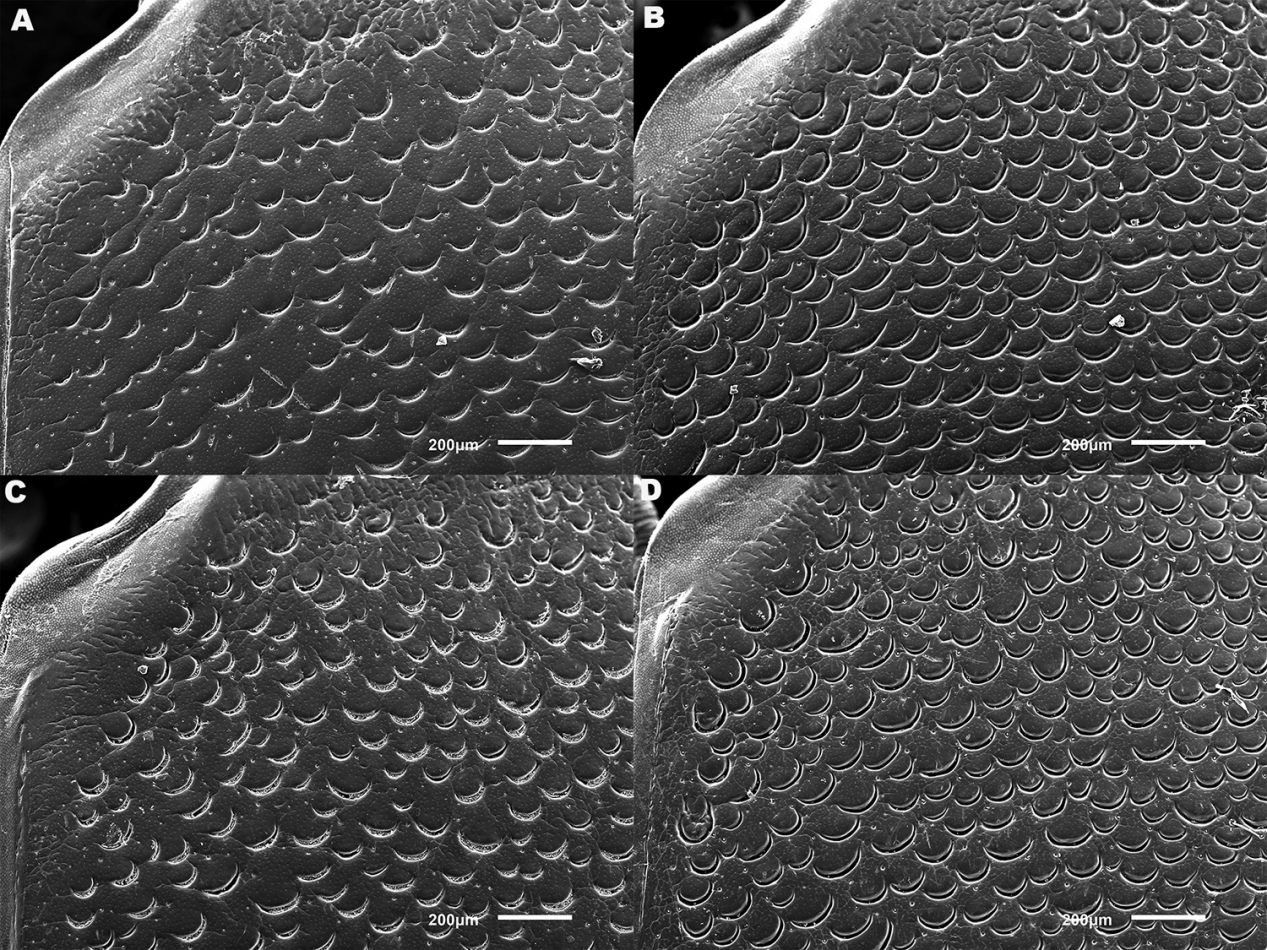
*Meladema* species elytral shoulder sculpture SEMs (DNA voucher codes where applicable). **A** *M. coriacea*, male, Spain, Murcia, Fte. Caputa **B** *M. lepidoptera* sp. n., male, Corsica, Cap Corse (NHM-IRM12F) **C** *M. coriacea*, female, Spain, Murcia, Fte. Caputa **D** *M. lepidoptera* sp. n., female, Corsica, Porto-Vecchio (NHM-IRM12A).

**Figure 8.**
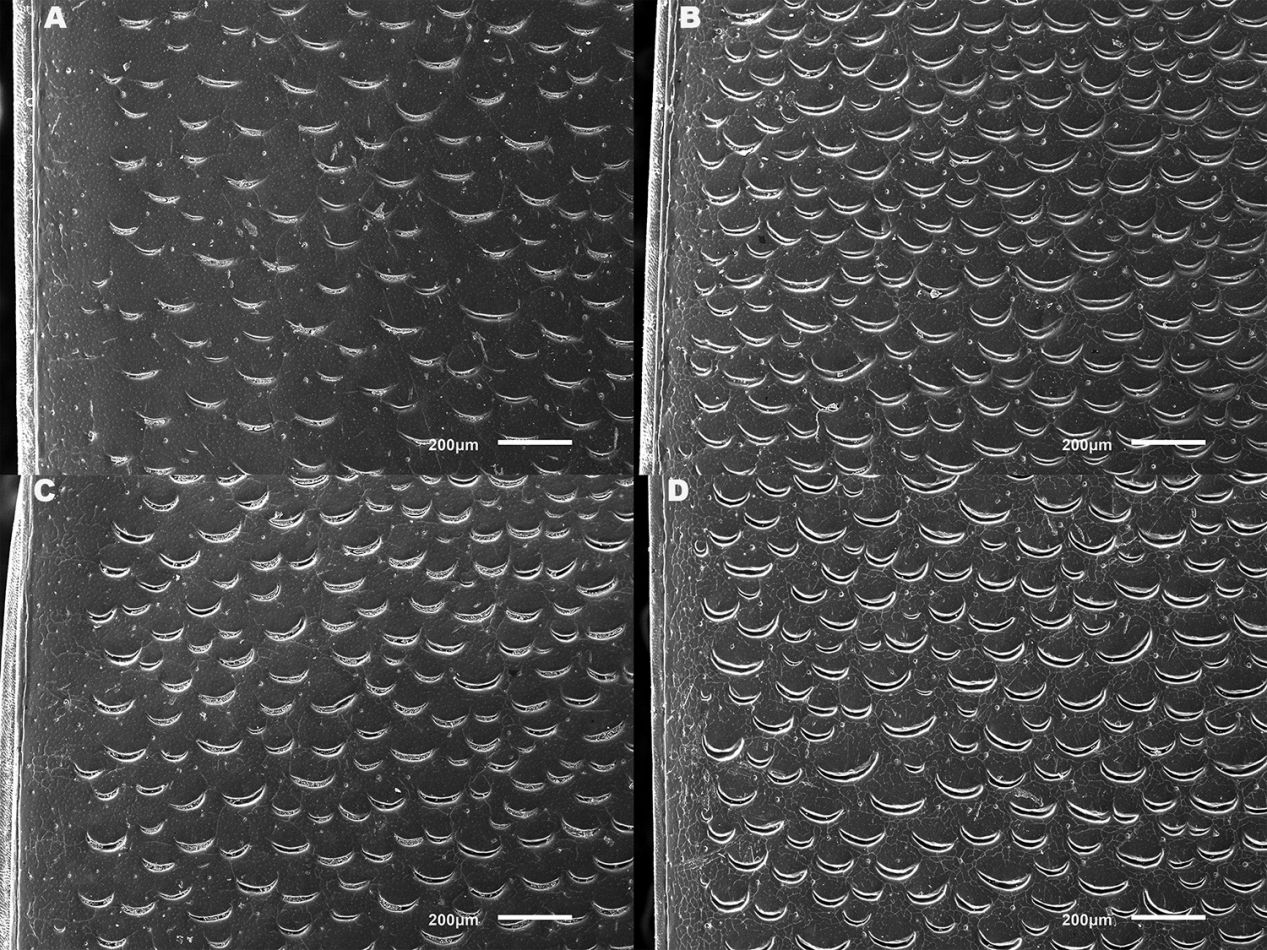
*Meladema* species elytral middle sculpture SEMs (DNA voucher codes where applicable). **A** *M. coriacea*, male, Spain, Murcia, Fte. Caputa **B** *M. lepidoptera* sp. n., male, Corsica, Cap Corse (NHM-IRM12F) **C** *M. coriacea*, female, Spain, Murcia, Fte. Caputa **D** *M. lepidoptera* sp. n., female, Corsica, Porto-Vecchio (NHM-IRM12A).

**Figure 9.**
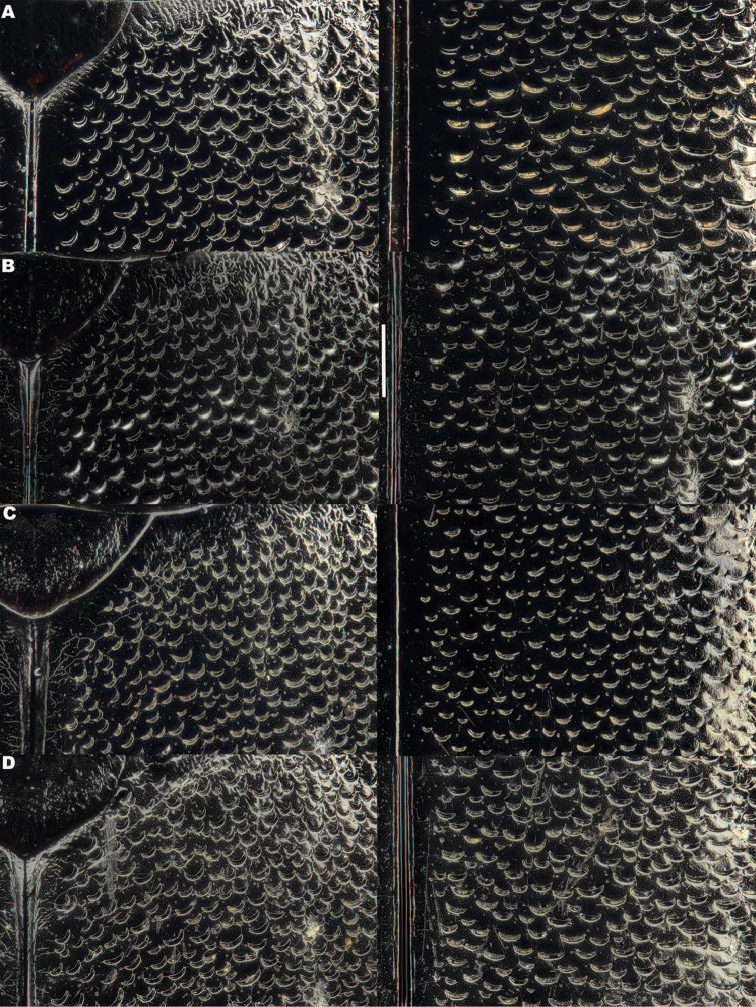
*Meladema coriacea* female elytral sculpture; shoulder and middle left and right, respectively (DNA voucher codes). **A** neotype, France, Var, La-Londe-les-Maures (NHM-IRM11C) **B** Spain, Córdoba, Baena (NHM-IRM14B) **C** Morocco, Tazzeka (NHM-IRM1A) **D** Algeria, Oued Bagrat (MNCN-HI4). Scale bar = 0.5 mm.

**Figure 10.**
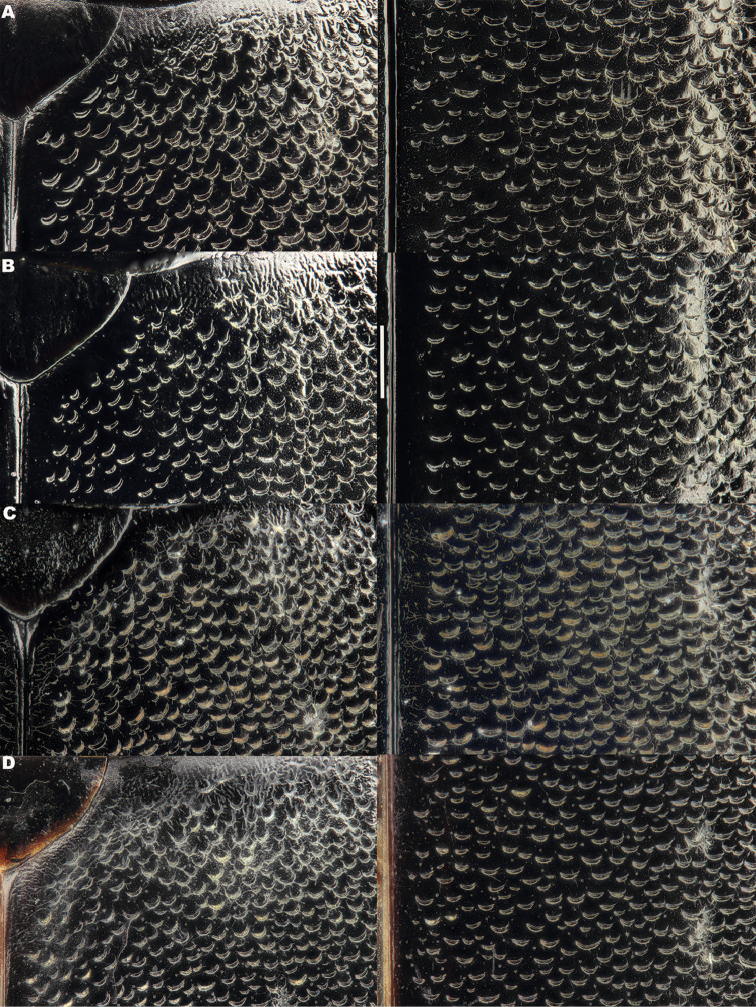
*Meladema coriacea* elytral sculpture; shoulder and middle left and right, respectively (DNA voucher codes). **A** Female, Turkey, Izmir (IBE-DV294) **B** male, Spain, Córdoba, Baena (NHM-IRM14A) **C** male, Morocco, Oued Massa (NHM-IRM2A) **D** male, Algeria, Aïn Damous (MNCN-HI6). Scale bar = 0.5 mm.

*Elytra*. Somewhat shining, with dense, transverse, crescentic striolae (Figures 5A, 6A) giving a scaly appearance (Figure 3A). Striolae less dense anteriorly and medially, here mostly distinctly separated from each other laterally (e.g. Figures 5A, 7A, C, 8A, C, 9, 10). Size of crescentic striolae variable, especially on disc close to suture (e.g. Figures 9, 10). Crescentic striolae becoming denser and somewhat continuous laterally and posteriorly (Figure 3A). Surface between crescentic striolae (Figures 5A, 6A) doubly punctate, with very fine, close punctures and medium, very sparse punctures (the latter bearing short, peg-like setae); also with fine, obsolete, open reticulation, usually more evident in apical two thirds, and more evident in some specimens than others; sometimes apparently absent. Puncture rows well-marked, continuous almost to elytral apices; punctures shallower posteriorly than anteriorly.

**Figure 11.**
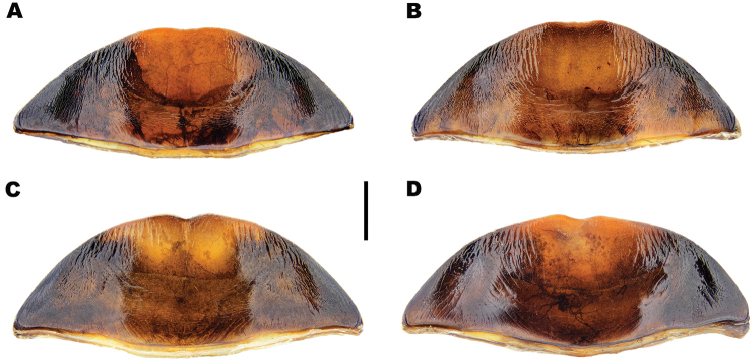
*Meladema* species males, abdominal ventrite 6 (DNA voucher codes where applicable). **A** *M. coricaea*, Spain, Murcia, Fte. Caputa **B** *M. lepidoptera* sp. n., Corsica, Cap Corse (NHM-IRM12F) **C** *M. imbricata*, La Gomera, El Cedro (NHM-IRM3A) **D** *M. lanio*, Madeira, Ribeira dos Cedros (NHM-IRM8A). Scale bar = 1 mm.

*Venter*. Prementum shining, tumid in centre, with fine and medium, sparse punctures. Mentum shining; central projection with shallow median emargination. Lateral lobes with medium, sparse punctures, and scattered, whitish recumbent to decumbent setae. Submentum shining, with transverse wrinkles centrally, and elongate wrinkles laterally. Central 1/4 with medium, sparse punctures bearing long, white-yellowish, erect setae. Gula shining, with sparse, shallow, transverse wrinkles; patch of medium-coarse punctures posterolaterally. Genae shining, with obsolete, open, elongate reticulation. Prosternum shining, with irregular transverse ridges laterally. Strongly arched in centre and with fine, moderate to close punctures laterally, bearing long, white-yellowish, recumbent to erect setae; punctures and setae extending in a sparse, irregular row onto process, just below arch. Process lanceolate, tectiform; apex acuminately rounded. Centre of prosternum and process with double punctation of very fine, moderate and medium, very sparse punctures. Pronotal hypomeron shining, impunctate. Elytral epipleurs shining, with fine wrinkles; irregular puncture row close to internal margin, from centre to close to apex; punctures bearing fine, whitish, erect setae. Metaventrite shining, central portion with sparse, transverse scratches and fine to very fine, sparse to very sparse punctures; not clearly forming two size classes. Metaventral process strongly reticulate, with transverse, rugose meshes and traces of fine, sparse punctures; small, central patch at base with very small reticulation meshes. Metaventral process relatively broad; apex acuminate and upturned slightly anterolaterally. Metacoxal lines almost reaching anterior border of metacoxae; shallow and interrupted in anterior 1/5. Internal laminae of metacoxae shining, sculpture as on centre of metaventrite. Metacoxal lobes sculptured as internal laminae, strongly rounded, with irregular, elongate field of medium to coarse punctures close to lateral margins, bearing fine, white, recumbent to erect setae. External laminae of metacoxae shining, smooth close to process, but with strong reticulation elsewhere; reticulation meshes very elongate posteromedially, to transverse anteriorly. Abdominal ventrites shining. Ventrites 3–5 with cluster of golden, erect setae anteromedially. Ventrite 1 with elongate reticulation throughout. Ventrite 2 with similar reticulation; absent close to centre. Ventrite 3 with elongate reticulation laterally, becoming transverse close to smooth central 1/5. Reticulation of ventrites 4 and 5 restricted to lateral third and with superimposed elongate furrows. Ventrites 2–5 doubly punctate; very fine, moderate and fine, sparse to very sparse punctures; punctures most evident in areas without reticulation. Ventrites 3–5 with transverse irregular row of long, yellowish, recumbent to decumbent setae laterally. Ventrite 6 (Figure 11A) with very fine, moderate punctures and medium to coarse, sparse to moderate punctures; punctures coarser close to apex. Elongate, semicircular wrinkles and channels apicolaterally. Some punctures in channels bearing elongate, whitish, erect setae.

**Figure 12.**
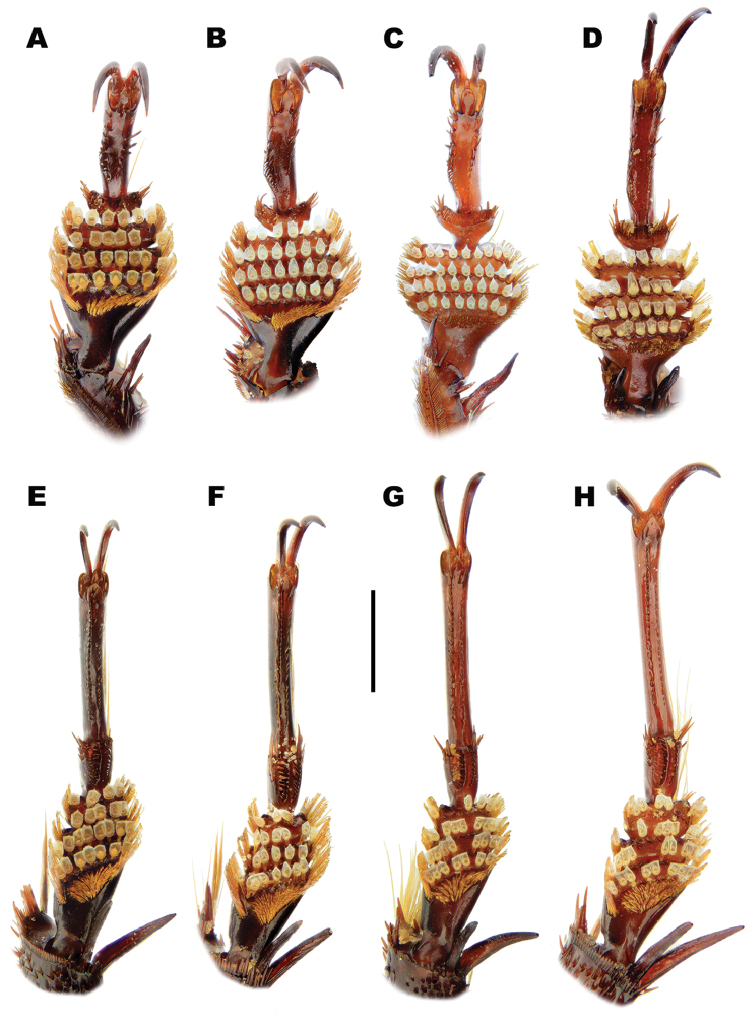
*Meladema* species males, fore (**A–D**) and mesotarsi (**E–H**), ventral view (DNA voucher codes where applicable). **A, E** *M. coriacea* Spain, Cáceres, nr. Plasencia **B, F** *M. lepidoptera* sp. n. holotype, Corsica, Cap Corse (NHM-IRM12E) **C, G** *M. imbricata*, La Gomera, El Cedro (NMH-IRM3A) **D, H** *M. lanio*, Madeira, Rabacal. Scale bar = 1 mm.

**Figure 13.**
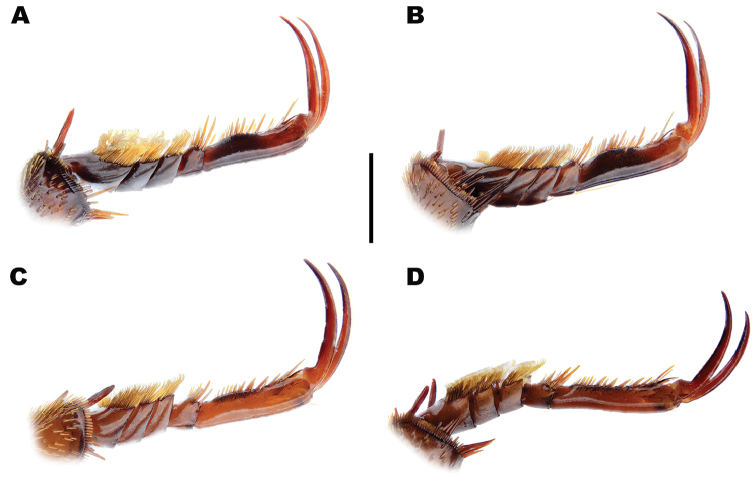
*Meladema* species males, fore tarsal claws, lateral view (DNA voucher codes where applicable). **A** *M. coriacea* Spain, Cáceres, nr. Plasencia **B** *M. lepidoptera* sp. n. holotype, Corsica, Cap Corse (NHM-IRM12E) **C** *M. imbricata*, La Gomera, El Cedro (NMH-IRM3A) **D** *M. lanio*, Madeira, Rabacal. Scale bar = 1 mm.

**Figure 14.**
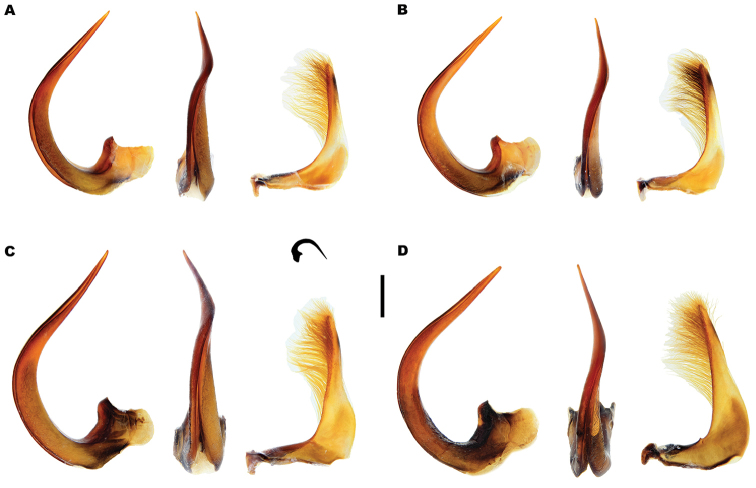
*Meladema* species male genitalia. Median lobe, lateral and ventral view; paramere (DNA voucher codes where applicable). **A** *M. coriacea* Spain, Cáceres, nr. Plasencia **B** *M. lepidoptera* sp. n. holotype, Corsica, Cap Corse (NHM-IRM12E) **C** *M. imbricata*, La Gomera, El Cedro (NMH-IRM3A) **D** *M. lanio*, Madeira, Rabacal. Silhouette indicates orientation of median lobe for imaging in ventral view. Scale bar = 1 mm.

*Male*. Foretarsi (Figure 12A) with articulo-setal counts as follows (base to apex): row 1, 7; row 2, 8–10; row 3, 8–10; row 4, 6-8. Number of setae in rows may differ from right to left tarsus in same beetle. Curved, golden setae bordering articulo-setal field dense, particularly basally. Foretarsal claws (Figures 12A, 13A) elongate, curved; interior margin straight or somewhat raised in basal two thirds, strongly curved in apical third. Mesotarsi (Figure 12E) with articulo-setal counts as follows (base to apex): row 1, 7–8; row 2, 8; row 3, 7; row 4, 4 (2 clusters of 2, situated laterally). Curved, golden setae bordering articulo-setal field dense, particularly basally. Mesotarsal claws (Figure 12E) elongate, strongly curved. Abdominal ventrite 6 (Figure 11A) with apex rounded, with very shallow median emargination. Median lobe asymmetrical (Figure 14A), sinuation weak, approximately 1/5–1/4 from apex; ventral margin of apical portion relatively straight in lateral view. Parameres (Figure 14A) with basal portion of inner margin relatively evenly curved; outer and inner margins almost straight over apical two thirds.

**Figure 15.**
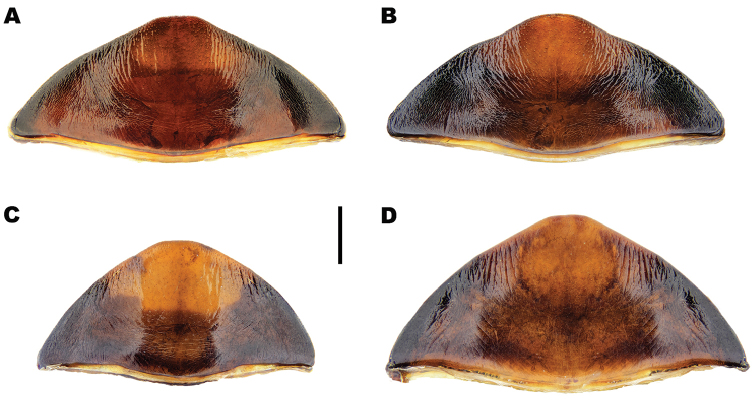
*Meladema* species females, abdominal ventrite 6 (DNA voucher codes where applicable). **A** *M. coricaea*, Spain, Murcia, Fte. Caputa **B** *M. lepidoptera* sp. n., Corsica, Cap Corse (NHM-IRM12C) **C** *M. imbricata*, La Palma, Bco. Hoyo Verde **D** *M. lanio*, Madeira, Ribeira dos Cedros (NHM-IRM9A). Scale bar = 1 mm.

*Female*. As male, except for simple fore and mesotarsi, differently shaped abdominal ventrite 6 (with bluntly pointed apex, Figure 15A). The density of crescentic striolae on the elytra does not differ consistently between sexes, contrary to the statement of Balfour-Browne (1948).

*Variation*. Variation is evident in a number of characters. The size and density of the crescentic striolae on the elytra differs between individuals (e.g. Figures 5A, 7A, C, 8A, C, 9, 10, 17, 18D, 19), and shows some geographical structure. Specimens of both sexes from the Iberian Peninsula (e.g. Figures 7A, C, 8A, C, 9B, 10B) have relatively sparse crescentic striolae, with individual striolae relatively small in size, features also seen in most specimens from the Balkans and Anatolia (e.g. Figure 10A). Some specimens from France, North Africa and southern Italy (e.g. Figures 9D, 10C, D, 19) have slightly denser striolae, with some of these slightly larger. Specimens from massifs in the central Sahara (Hoggar and Tassili n’Ajjer (Algeria), Tibesti (Chad)) have very dense crescentic striolae (Figure 17). In these beetles the striolae are relatively small and strongly curved, giving a distinctly tuberculate appearance to the elytra, even close to the shoulders. In no cases do the elytral striolae approach the condition seen in *M. lepidoptera* sp. n., however (see below). The degree of curvature of male foretarsal claws differs between individuals, even within the same population. Some specimens have claws which are shorter, and broader at their apices that in Figure 13A, these almost certainly being older insects, whose claws have become worn down during life. The size and shape of the median lobe also varies slightly between individuals and populations (e.g. Figure 20). Specimens from North Africa, for example, (Figure 20H–K) have narrower apical portions, whilst the largest median lobes are seen in Greek animals (Figure 20M–N).

#### Differential diagnosis.

Morphologically, this species is almost identical to *M. lepidoptera* sp. n., something which has prevented the latter’s formal description until now. The two species can be reliably separated only on details of their elytral sculpture, *M. coriacea* having smaller, less dense crescentic striolae than *M. lepidoptera* sp. n., this being particularly evident at the elytral base, close to the scutellum, and in the middle, close to the suture (see Figure 1 and e.g. Figures 7A, C, 8A, C, 9, 10 vs. 7B, D, 8B, D, 21, 22). In *M. coriacea*, the striolae are largely well separated from each other in both regions (see comment by Aubé 1836, quoted above) whereas in *M. lepidoptera* sp. n. they are typically much more contiguous (see below). In the mid-elytra of *M. coriacea* there is also typically a distinct gap between the elytral suture and the first crescentic striolae (see e.g. Figure 8A, C), whereas in *M. lepidoptera* sp. n. the striolae begin much closer to the suture (e.g. Figure 8B, D). Whilst there is some variation, as illustrated in the figures, these features appear consistent throughout the wide geographical range of *M. coriacea*. Other characters, including male genitalia, claws, and the shape and sculpture of abdominal ventrite 6 in both sexes, do not differ consistently between the two species, there being as much intraspecific as interspecific variation.

The genetic differences between *M. coriacea* and *M. lepidoptera* sp. n. are well defined and comparable to those seen between *M. imbricata* and *M. lanio*, although mostly seen in mitochondrial markers (Sýkora et al. 2017). Of the two nuclear markers sequenced, H3 was identical in *M. coriacea* and *M. lepidoptera* sp. n. (but different from *M. lanio* and *M. imbricata*), only wingless having some diagnostic positions separating the two species (see Sýkora et al. 2017).

#### Distribution.

Even as redefined here, this is by far the most widespread species of the genus, distributed from the Canary Islands to Turkey, and south to massifs of the central Sahara (Figure 23). We have examined material from all countries from which this species has been reported, with the exception of Bulgaria (Guéorguiev 1987). Despite being listed from Lybia in the Palaearctic and World Catalogues of Dytiscidae (Nilsson and Hájek 2017a, 2017b), all Saharan records which we have traced emanate from Algeria and Chad. As discussed by Sýkora et al. (2017), basal splits within the *M. coriacea* lineage apparently took place in north-west Africa, with subsequent range expansion across the western Mediterranean, and several apparently independent, recent, colonisations of the Canary Islands from Morocco. The most easterly specimens included in genetic analyses (from Malta and Izmir, Turkey) were more closely related to beetles from north Africa than those from Iberia or southern France, suggesting that colonisation of the eastern part of the species range occurred via dispersal from Algeria/Tunsia, likely through Malta, Sicily and southern Italy. Genotyped individuals have been studied from throughout the wide range of *M. coriacea*, as well as a large number of additional non-genotyped specimens (Figure 23). The exact limits of *M. coriacea* in the Italian Peninsula remain unclear, and can only be resolved through combined genetic and morphological study of additional, fresh material. It is apparent, however, that *M. coriacea* is present in at least Sicily and that *M. lepidoptera* sp. n. occurs in the north and centre of the peninsula (Toscana and Lazio – as confirmed from genotyped specimens). Beetles morphologically intermediate between *M. coriacea* and *M. lepidoptera* sp. n. have been seen from Apulia and Campania (Figure 18B, C), suggesting possible hybridization between the two species where their ranges come into contact. Such a process is already established to have occurred between *M. coriacea* and *M. imbricata* on Tenerife (see below and Ribera et al. 2003). *Meladema coriacea* is a species typical of seasonally fluctuating or intermittent Mediterranean stream systems, being particularly characteristic of localities with winter-spring flow, which dry down to pools in summer. It is associated with drier and more seasonally variable conditions than other species of the genus, something which may have facilitated its range expansion (Sýkora et al. 2017).

### Meladema lepidoptera sp. n.

Taxon classificationAnimaliaColeopteraDytiscidae

http://zoobank.org/C28258E3-0B86-4983-AB52-7C1301A4A24A

Figures 3B , 4B , 5B , 6B , 7B, D , 8B, D , 11B , 12B, F , 13B , 14B , 15B , 18A , 20O–U , 21 , 22 , 23

#### Type locality.

France, Corsica, Cap Corse, stream nr. Bettolacce, 42°58'2.4"N 9°24'42.4"E.

#### Type material

**(genotyped specimens only).** Holotype ♂: “11 FR Corsica 21.ix.1999// Cap Corse: Bettolacce// 42°58'2.4"N 9°24'42.4"E 250m// I.Ribera & A. Cieslak leg.” “DNA voucher// NHM-IRM12E” “*Meladema lepidoptera*// Bilton & Ribera, 2017//HOLOTYPE” (NMW). Dry card mounted, tissue samples and DNA aliquotes, with same data, in IBE. Sequence data from the holotype has been deposited in GenBank with accession numbers AF428206 (COI-3’) and AF428188 (16S ribosomal RNA). **Paratypes (13)**: 1 ♂ “11 FR Corsica 21.ix.1999// Cap Corse: Bettolacce// 42°58'2.4"N 9°24'42.4"E 250m// I.Ribera & A. Cieslak leg.”“DNA voucher// NHM-IRM12F” (NMW); 1 ♀ “11 FR Corsica 21.ix.1999// Cap Corse: Bettolacce// 42°58'2.4"N 9°24'42.4"E 250m// I.Ribera & A. Cieslak leg.” “DNA voucher// NHM-IRM12C” (BMNH); 1 ♀ “5 FR Corsica 19.ix.1999// Porto-Vecchio, l’Ospedale// 41°39'13.7"N 9°12'41.0"E 690m// I.Ribera & A. Cieslak leg.” “DNA voucher// NHM-IRM12A” (CBP); 1 ♀ “5 FR Corsica 19.ix.1999// Porto-Vecchio, l’Ospedale// 41°39'13.7"N 9°12'41.0"E 690m// I.Ribera & A. Cieslak leg.” “DNA voucher// NHM-IRM12D” (MNHN); 1 ♀ “5 FR Corsica 19.ix.1999// Porto-Vecchio, l’Ospedale// 41°39'13.7"N 9°12'41.0"E 690m// I.Ribera & A. Cieslak leg.” “DNA Voucher// NHM-IRM12g” (IBE); 1 ♀ “9 FR Corsica 19.ix.1999// Ghisoni, road to Campannella// 42°4'8.7"N 9°11'6.0"E 830m// I.Ribera & A. Cieslak leg.” “DNA Voucher// NHM-IRM12b” (IBE); 1 ♂ “Toscana (PI) S. Luce// ‘Boso’ de Castagni, s tr. s.// Luce-Castellina Marittima// 3.X.2007 leg. M. Toledo” “DNA voucher// IBE-AN693” (CTP); 1 ♀ “April 2015 Italy Monti della Tolfa// Rio Ippovia della Cicugnola// (pozze ruscellamento)// 42°4'26.27"N 11°56'12.25"E// V. Buono leg.” “DNA voucher// IBE-AN760” (CVR); 1 ♂ “I. D’ELBA – POMONTE// Fosso BARIONE// m 250-300// 29.V.94 TOLEDO LGT.” [HW] “DNA voucher// IBE-AN692” (CTP); 1 ♂ “3/vi/2014 ITALY Montecristo// 42.334N 10.308E 260m// R. Vila leg.” “DNA voucher// IBE-DV289” (IBE); 1 ♂ “3/vi/2014 ITALY Montecristo// 42.334N 10.308E 260m// R. Vila leg.” “DNA voucher// IBE-DV290” (ISNB); 1 ♂ “27/v/2009 ITALY Sardinia// Nuoro prov.(Ogliastra historical region)// brook 562m ESE Villagrande Strìsaili (WNW Tortoli)// 39.95084N 9.51912E H. Fery & M. Toledo leg.” “DNA voucher// IBE-RA5” (MNCN); 1 ♀ “27.5.2009 Italy, Sardinia// Ogliastra prov., ca. 5 km// WNW Tortoli, (on road// Tortoli – Villagrande Stris.)” “39.93982N 9.59280E// ca 80m, brook// Fery & Toledo leg.” “DNA voucher// IBE-RA18” (MNCN). Each with red label “*Meladema lepidoptera*// Bilton & Ribera, 2017//PARATYPE”. All dry card mounted, tissue samples and DNA aliquotes, with same data, in IBE.

#### Additional material examined

**(non-genotyped specimens). France, Corsica**: 2 ♂♂, 1 ♀ “11/iv/1993// Corsica Francardo// Mediterranean stream// D. T. Bilton leg.” (CBP); 1 ♀ “Calvi// 29.VIII.” “Pietra// Maggiore” “KORSIKA// VIII.1955” “Meladema// coriacea Cast.// M/ Balke det. 1990” [Latin name, describer & 90 HW] (NMW); 1 ♂, 2 ♀♀ “12.4.79 Korsika// Pinito, ca 700m// Bach” [HW] reverse “Fery leg.” “Mel.// coriacea// Cast.” [HW] (NMW); 1 ♀ “12.4.79 Korsika// Pinito, ca 700m// Bach” [HW] reverse “Fery leg.” “Mel.// coriacea// Cast.” [HW] “coll. Shaverdo” (NMW); 1 ♀ “Korsika// 7.80” [HW] “Meladema// coriacea Cast.// M. Balke 1990” [Latin name, describer & 90 HW] (NMW); 1 ♂ “Corse” [HW] “Reveliere” [yellow, square label, HW] “Coriaceus// Corsica” [HW] “C. Epplsh.// Steind. d.” (NMW); 1 ♂ “Corsica” (NMW); 1 ♀ “12.4.79 Korsika// Pinito, ca. 700m// Bach” [HW] “Mel.// coriacea// Cast.” [HW] “Coll.// HENDRICH// Berlin” (ZSM). **Italy, Sardinia**: 5 ♂♂, 3 ♀♀ “Sardinia (CA) Dolianova// Rio Flumini 30.VI.91// leg. Meloni” (CBP, CTP); 3 ♂♂, 1 ♀ “SARDINIEN// Bosa// 24.5-24.6.//1963, Budberg” reverse “Meladema// coriaceum Lap.” [HW] (NMW); 1 ♀ “coll. Win-// gelmüller” “coriaceum Lap.// Sardinien” [HW] (NMW); 1 ♂ “Villasimius// Sard. m. 19.9.59// E. Jünger” [19 HW] “A7598// coricae Cast.// det. K. Hoch 1959” [number, Latin name, describer & 9 HW] (ZSM); 1 ♀ “Sardegna// Lode// 28.7.79// S. Gottwalt” [HW] “coll.//HENDRICH// Berlin” (ZSM); 2 ♀♀ “Sardinien: bei Nuvafus,// 09.IX.1978” “Meladema// coriacea// Cast.” [HW] “entnommen// 1 Exempl.” [HW] “Sardinien// S. Nuvafus// 9.9.78” [HW] (ZSM); 1 ♂, 1 ♀ “Sardinien: S Monte// Limbara. Tempio, 650m,// 28.VIII.1978” “Meladema// coriacea// Cast.” [HW] “Entnommen// 1 Exempl.” [HW] “Sardinien// Südl. Monte. Liombara// Tempio 650m// 28.8.78” [HW] “Fuss Monte// Limbara 650m// 28.8.78” [HW] (ZSM); 1 ♀ “Sardinien: Lagoatto di// Flumendosa, Villanova// Strisaili, 05.IX.1978” “Meladema// coriacea// Cast.” [HW] “Sardinien// Lagoatto di Flumendosa// Villanova Strisaili// 5.9.1978” [HW] (ZSM); 6 ♂♂, 3 ♀♀ “Meladema// coriacea Cast.// Italien/ Sardinien// Giara di Gesturi// - 9.1980// Coll./ leg.// Burmeister” “Sardinien// Giara di Gesturi// 9.1980// leg. Burmeister” [HW] “Meladema// coriacea// Cast.” [HW] (ZSM); 1 ♂ “Meladema// coriacea Cast.// Italien/ Sardinien// b. Tempio// Bergbach am// Monte Limbarra// - 9.1980// Coll./ leg.// Burmeister” “Sardinien// b. Tempio// Bergbach am// Monte Limbarra// 9. 1980” [HW] “Meladema// coriacea// Cast.” [HW] (ZSM); 2 ♂♂, 2 ♀♀ “Meladema// coriacea Cast.// Italien/ Sardinien// Pass b. Genne// Cruxi// 1.9.1980// Coll./ leg.// Burmeister” “Sardinien// Pass b. Genne Cruxi// 10. [circled]// leg. Burmeister” [HW] (ZSM); 2 ♀♀ “Meladema// coriacea Cast.// Italien/ Sardinien// b. Nuragus// (Viehtränke)// 3.9.1980// Coll./ leg.// Burmeister” “Sardinien// b. Nuragus// Viehtränke// 3.9.1980 23. [circled]// leg. Burmeister” [HW] “Meladema// coriacea// Cast.” [HW] (ZSM); 1 ♂ “Meladema// coriacea Cast.// Italien/ Sardinien// b. Tempio// Bergbach// b. Mnte Limbarra// - 9.1980// Coll./ leg.// Burmeister” “Sardinien// b. Tempio, Bergbach// b. Monte Limbarra// 9/1980 39. [circled]// leg. Burmeister” [HW] ‘Meladema// coriacea// Cast.” [HW] (ZSM); 1 ♂ “Glasgow University// HUNTERIAN MUSEUM// ex. coll. G.N. Foster” “Sardinia 32T UTH// 04602/44750// S of Mont Minerva// G N Foster 16.10.2006” (CFA); 1 ♀ “27.5.2009 Italy, Sardinia// Ogliastra prov., ca. 5 km// WNW Tortoli, (on road// Tortoli – Villagrande Stris.)” “39.93982N 9.59280E// ca 80m, brook// Fery & Toledo leg.” (IBE). 1 ♂ “9 Sardinia, Calangianus 11.iv.2017// stream, way to Pascaredda tomb// 40°54'35.6"N 9°10'12.8"E 435m// I.Ribera & A.Cieslak leg.” (IBE); **Italy, Elba**: 1 ♂ [no head] “Ins. Elba// 1908// Paganetti” (NMW); 2 ♂♂ “Elba: bei Marciana, Bach in// Kastanienwald, 17.IX.1975,// leg.: Schmalfuss” “17.9.75 Elba, bei Murciana// Bach in Kastanienwald// Schmalfuss leg.” [HW] “Meladema// coriaceum// Lap.” [HW] “Meladema coriacea Cast.” [HW] (ZSM). **Italy, mainland**: 5 ♂♂, 3 ♀♀ “Levante,// Liguria, Italy.” “Coll. Odier.// B.M. 1921-288.” (BMNH); 1 ♂, 1 ♀ “Levante,// Liguria, Italy.” “Coll. Odier.// B.M. 1921-288.” “Meladema// coriacea Cast// C.R. Smith det. 1982” [Latin name, describer & 2 HW] (BMNH); 1 ♀ “ITALY: Liguria.//Torr. 2km W. of Pogli,// trib. of Torr. Arroscia, nr. Albenga, 7.iv.1958// J. Balfour-Browne.” “Brit. Mus.// 1960-482.” [482 HW] “Meladema// coriacea Cast// C.R. Smith det. 1982” [Latin name, describer & 2 HW] (BMNH); 3 ♂♂, 1 ♀ “Italia// Ruta// 2-6. VII.95// A. Fiori” [HW, A. Fiori printed] (NMW); 1 ♀ “coriaceus// Toscana// V. Heyden” [HW] “c. Epplsh.// Steind. d.” (NMW); 1 ♂ “S. gimignano// Sienna → Florenz// 3.8.36 Eiselt” [HW] “Meladema// coriaceum” [HW] (NMW); 1 ♂ “San Gimignano// IX 36 EISELT” “Meladema// coriaceum Lap.” [HW] (NMW); 1 ♀ “San Gimignano// IX 36 EISELT” (NMW); 1 ♂ “Nervi// 27.III.05” [HW] (ZSM); 2 ♀♀ “Italia// 26.3.05” [date HW] “Nervi” (ZSM); 1 ♀ “Italien// 11.xii.05” [HW] “♀” “Samml. A.// Zimmermann” (ZSM); 1 ♀ [small, circular brown label, no text] “Pisa” [HW] “alte// Sammlung” (ZSM); 1 ♀ “Italia: Umbria, NE Gosparini,// 550m, dry stream, 09.IX.2015// 43°14'31.73"N, 12°6'19.69"E,// leg. Komarek, Beutel (TM7)” “Meladema// coriacea Lap.// det. H. Shaverdo 2016” [Latin name, describer & 16 HW] (NMW). **Without locality data**: 1 ♀ [small, circular brown label, no text] “Coll. Odier.// B.M. 1921-288.” (BMNH); 1 ♀ “coriaceus” [HW] “E Coll.// Curtis” [HW] (BMNH); 1 ♂ “40// 4 2// 2135” [round label, HW] (BMNH). All with “*Meladema lepidoptera*// Bilton & Ribera// D T Bilton [or I Ribera] det. 2017”.

#### Description.

*Size*: Holotype TL = 20.74 mm; EL = 15.74 mm; MW = 10.50 mm. Other material examined TL = 19.20–20.99 mm; EL = 14.98–16.38 mm; MW = 9.73–11.39 mm.

*Colour*. Dorsum dark reddish brown to black (Figure 3B); lateral margins of pronotum, labrum and anterior half of clypeus somewhat paler, sometimes with diffuse lateral maculae. Elytra unicolorous, without distinct mottling even when lifted (Figure 4B). Head with a pair of oval, reddish yellow medial interocular patches, slightly elongated apicolaterally. Antennae and maxillary and labial palpi reddish yellow. Legs dark reddish brown to black with golden yellow setae; large spines somewhat paler. Venter reddish brown to black; gula, meso and metacoxae and trochanters paler.

*Head*. Labrum shining, with moderate to coarse, sparse punctures. Reticulation absent in apical half, becoming increasingly more evident basally, here forming weakly impressed, transverse meshes. Clypeus and anterior half of frons shining, doubly punctate, without reticulation and with very close, fine and very sparse, coarse punctures. Coarse punctures approximately 5–8x diameter of fine; without visible reticulation. Paired epicranial foveae, one immediately behind the other, on each side of frons, close to lateral margins and immediately behind lateral remnants of frontoclypeal suture. Anterior epicranial foveae transverse, posterior slightly elongate oval; both with cluster of stout, yellow recumbent to decumbent setae. Areas between anterior and posterior foveae with coarse wrinkles. Posterior frons with open, elongate, wrinkled reticulation, especially alongside lateral margins of compound eyes and onto vertex; meshes tumid, with rugose appearance. Internal and posterior borders of compound eyes distinct, raised relative to level of adjacent cuticle. Lateral margins bordered by distinct narrow channel; deeper anteriorly than posteriorly and continuing behind posterior margin of eye onto vertex. Channel with dense punctures, bearing long, stiff, yellow recumbent to decumbent setae.

*Pronotum*. Posterior margin strongly sinuate laterally (Figure 3B). Surface somewhat shining, strongly rugose. Reticulation meshes large, open, almost isodiametric and relatively flat either side of mid-line on disc; smaller, tumid and more uneven in size and shape towards all margins. Transverse irregular row of medium punctures bearing long, yellow recumbent to decumbent setae 1/5 behind anterior pronotal margin; interrupted briefly in centre, continuing inside lateral margins and inside lateral third of posterior margin. Centre of disc with elongate, narrow, slit-like fovea, sometimes partially interrupted in mid-length. Lateral margins slightly raised, shining, without rugose sculpture and with fine, scattered punctures.

*Elytra*. Somewhat shining, with dense, transverse, sometimes contiguous, crescentic striolae, giving a very scaly appearance (Figure 3B). Striolae relatively dense throughout, frequently contacting each other laterally on shoulder close to suture (e.g. Figures 5B, 7B, 18A, 21, 22, and relatively dense in mid-elytra close to suture (e.g. Figures 7D, 8D, 18A, 21, 22)). Size of crescentic striolae relatively large, both in shoulder and mid-elytral regions (e.g. Figures 7B, D, 8B, D, 18A, 21, 22). Crescentic striolae becoming denser and somewhat continuous laterally and posteriorly. Surface between crescentic striolae (Figures 5B, 6B) doubly punctate, with very fine, close punctures and medium, very sparse punctures (the latter bearing short, peg-like setae); also with fine, obsolete, open reticulation, usually more evident in apical two thirds, and more evident in some specimens than others. Puncture rows well-marked, continuous almost to elytral apices; punctures shallower posteriorly than anteriorly.

*Venter*. Prementum shining, tumid in centre, with fine and medium, sparse punctures. Mentum shining; central projection with shallow median emargination. Lateral lobes with medium, sparse punctures, and scattered, whitish recumbent to decumbent setae. Submentum shining, with transverse wrinkles centrally, and elongate wrinkles laterally. Central 1/4 with medium, sparse punctures bearing long, white-yellowish, erect setae. Gula shining, with sparse, shallow, transverse wrinkles; patch of medium-coarse punctures posterolaterally. Genae shining, with obsolete, open, elongate reticulation. Prosternum shining, with irregular transverse ridges laterally. Strongly arched in centre and with fine, moderate to close punctures laterally, bearing long, white-yellowish, recumbent to erect setae; punctures and setae extending in a sparse, irregular row onto process, just below arch. Process lanceolate, tectiform; apex acuminately rounded. Centre of prosternum and process with double punctation of very fine, moderate and medium, very sparse punctures. Pronotal hypomeron shining, impunctate. Elytral epipleurs shining, with fine wrinkles; irregular puncture row close to internal margin, from centre to close to apex; punctures bearing fine, whitish, erect setae. Metaventrite shining, central portion with sparse, transverse scratches and fine to very fine, sparse to very sparse punctures; not clearly forming two size classes. Metaventral process strongly reticulate, with transverse, rugose meshes and traces of fine, sparse punctures; with small central patch of reticulation with very small meshes. Metaventral process relatively broad; apex acuminate and upturned slightly anterolaterally. Metacoxal lines almost reaching anterior border of metacoxae; shallow and interrupted in anterior 1/5. Internal laminae of metacoxae shining, sculpture as on centre of metaventrite. Metacoxal lobes sculptured as internal laminae, strongly rounded, with irregular, elongate field of medium to coarse punctures close to lateral margins, bearing fine, white, recumbent to erect setae. External laminae of metacoxae shining, smooth close to process, but with strong reticulation elsewhere; reticulation meshes very elongate posteromedially, to transverse anteriorly. Abdominal ventrites shining. Ventrites 3–5 with cluster of golden, erect setae anteromedially. Ventrite 1 with elongate reticulation throughout. Ventrite 2 with similar reticulation; absent close to centre. Ventrite 3 with elongate reticulation laterally, becoming transverse close to smooth central 1/5. Reticulation of ventrites 4 and 5 restricted to lateral third and with superimposed elongate furrows. Ventrites 2–5 doubly punctate; very fine, moderate and fine, sparse to very sparse punctures; punctures most evident in areas without reticulation. Ventrites 3–5 with transverse irregular row of long, yellowish, recumbent to decumbent setae laterally. Ventrite 6 (Figure 11B) with very fine, moderate punctures and medium to coarse, sparse to moderate punctures; punctures coarser close to apex. Elongate, semicircular wrinkles and channels apicolaterally. Some punctures in channels bearing elongate, whitish, erect setae.

*Male*. Foretarsi (Figure 12B) with articulo-setal counts as follows (base to apex): row 1, 7; row 2, 8–10; row 3, 8–10; row 4, 6–8. Number of setae in rows may differ from right to left tarsus in same beetle. Curved, golden setae bordering articulo-setal field dense, particularly basally. Foretarsal claws (Figures 12B, 13B) elongate, curved; interior margin straight or somewhat raised in basal two thirds, strongly curved in apical third. Mesotarsi (Figure 12F) with articulo-setal counts as follows (base to apex): row 1, 7–8; row 2, 8; row 3, 7; row 4, 4 (2 clusters of 2, situated laterally). Curved, golden setae bordering articulo-setal field dense, particularly basally. Mesotarsal claws (Figure 12F) elongate, strongly curved. Abdominal ventrite 6 (Figure 11B) with apex rounded, with very shallow median emargination. Median lobe asymmetrical (Figure 14B), sinuation weak, approximately 1/5–1/4 from apex; ventral margin of apical portion relatively straight in lateral view. Parameres (Figure 14B) with basal portion of inner margin relatively evenly curved; outer and inner margins almost straight over apical two thirds.

*Female*. As male, except for simple fore and mesotarsi, differently shaped abdominal ventrite 6 (with bluntly pointed apex - Figure 15B). As with *M. coriacea*, no consistent differences between males and females are evident in terms of elytral sculpture.

*Variation*. The size and density of the crescentic striolae on the elytra differs somewhat between individuals and localities (Figures 5B, 7B, D, 8B, D, 18A, 21, 22), these being relatively dense in mainland Italy, Corsica and the Tuscan archipelago, and less so in most Sardinian specimens (e.g. Figure 22). The combination of size and density of these striolae is always greater than seen in *M. coriacea*, however (see above). The degree of curvature of male foretarsal claws differs between individuals, as in *M. coriacea*, as does the size and shape of the median lobe (e.g. Figure 20O–U).

#### Differential diagnosis.

Morphologically almost identical to *M. coriacea* (see above). Only distinguishable on the size, shape and density of crescentic striolae on the elytra, which give *M. lepidoptera* sp. n. a very scaly appearance, evident even at relatively low magnification (e.g. Figure 3A vs. 3B). See above for genetic differences between this species and *M. coriacea*.

#### Etymology.

From the ancient Greek “lepidos” (λεπίδος, scale, but also referring to roof tiles) and “pteron” (πτερόν, wing). The specific epithet is a noun in the nominative plural.

#### Distribution.

On the basis of current data, found on Corsica and Sardinia, islands of the Tuscan Archipelago (Elba, Montecristo) and parts of peninsular Italy, from Liguria to Umbria (Figure 23). *M. lepidoptera* sp. n. is apparently the only species of the genus found on Corsica, Sardinia, Elba and Montecristo (past records of *M. coriacea* from these islands - e.g. Poggi 1976, Franciscolo 1979, Dettner 2007 - almost certainly referring to this species), but co-occurs with *M. coriacea* in the Italian peninsula. The exact limits of the distribution of the two species in peninsular Italy remain unclear (see above), but there is morphological evidence suggesting hybridization where they meet, at least in the south (see below). The contact zone between the two species in the north appears to be situated on the Mediterranean coast, somewhere close to the French-Italian border, but to date no intermediate specimens have been seen from this area. Clearly future work, using both genetic and morphological approaches, would be illuminating in understanding the location and dynamics of these contact zones. As with other extant *Meladema* lineages, this species appears to have originated in the early Pleistocene, colonisation of the Tyrrhenean islands occurring long after the Messinian Salinity Crisis (Ribera et al. 2003, Sýkora et al. 2017). Sýkora et al. (2017) suggest that *M. lepidoptera* sp. n. may have originated following the colonization of the Tyrrhenian islands, a hypothesis which should be tested in the future through genetic study of more individuals from peninsular Italy. Sýkora et al. (2017) additionally suggest that this species is characteristic of sites with lower seasonality than is typical for *M. coriacea*, based on MaxEnt modelling. They note that many of the peninsular Italian localities (obtained from the literature) included in their analyses (from which specimens were not studied) fitted into the climatic space occupied by *M. lepidoptera* sp. n. (as ‘*coriacea* CSM’), not surprising given our finding that this species does indeed occur on the Italian mainland. Clearly it would be interesting to repeat Sýkora et al.’s (2017) analyses in the future, once the range limits of these taxa are better established. On the basis of current evidence, this species occurs in similar habitats to those occupied by *M. coriacea*, although the two taxa have not been detected to date in the same locality.

### Meladema imbricata

Taxon classificationAnimaliaColeopteraDytiscidae

(Wollaston, 1871)

Figures 3C , 4C , 5C , 11C , 12C, G , 13C , 14C , 15C , 23 , 24A , 25D, F

Scutopterus imbricatus Wollaston, 1871: 220.
Meladema imbricata (Wollaston, 1871): Sharp 1882: 824; Régimbart 1895: 184; Machado 1987: 58; Balke et al. 1990: 364.
Meladema lanio ab. imbricata (Wollaston, 1871): Gcshwendtner 1936: 42.
Meladema imbricatum Branden, 1885: 95.
Meladema lanio f. imbricata Sanfilippo, 1966: 49.

#### Type locality.

“Madeira” [mislabelled].

#### Type material

**(BMNH).** Holotype ♀ (Figure 24A): “Scutopterus// imbricatus, Woll” [HW] Scutopterus// imbricatus Woll// M.E.Bacchus det 1977// HOLOTYPE” [Latin name, describer & last 7 HW] “Holo-// type” [small, circular label, red margin] “Meladema// imbricata (Woll.)// M. Balke det. 1989” [Latin name, describer & 89 HW] (dry pinned, BMNH, Wollaston Collection).

Note that as discussed by Machado (1987), the type specimen must have been mislabelled, as this species is now known to be endemic to the Canary Islands.

#### Additional material examined

**(genotyped specimens). Spain, Canary Islands.** 1 ♂ “1998 SPAIN Islas Canarias// La Gomera// El Cedro – stream in laurysilva// D. T. Bilton leg.” “M. IMBRICATA” [HW] “G4 Mel// below G3” [HW] “DNA voucher// NHM-IRM3A” (IBE); 1 ♂ “1998 SPAIN Islas Canarias// La Gomera// El Cedro – stream in laurysilva// D. T. Bilton leg.” “M. IMBRICATA” [HW] “G1 Mel” [HW] “DNA voucher// NHM-IRM4A” (IBE); 1 ♂ “1998 SPAIN Islas Canarias// La Gomera// El Cedro – stream in laurysilva// D. T. Bilton leg.” “DNA voucher// NHM-IRM4b” (IBE); 1 ♀ "15/i/2000 SPAIN Islas Canarias// La Gomera// El Cedro// D. T. Bilton leg." "DNA voucher// NHM-IRM15A" (CBP); 1 ♂ "15/i/2000 SPAIN Islas Canarias// La Gomera// El Cedro// D. T. Bilton leg." "DNA voucher// NHM-IRM15B" (CBP); 1 ♂ “April 1999 SPAIN// Islas Canarias La Palma// Bco. Hoyo Verde Caldera de// Taburiente D. T. Bilton leg.” “DNA voucher// NHM-IRM6A” (CBP); 1 ♀ “April 1999 SPAIN// Islas Canarias La Palma// Bco. Hoyo Verde Caldera de// Taburiente D. T. Bilton leg.” “DNA voucher// NHM-IRM6B” (CBP); 1 ♀ “April 1999 SPAIN// Islas Canarias La Palma// Bco. Hoyo Verde Caldera de// Taburiente D. T. Bilton leg.” “DNA voucher// NHM-IRM6C” (CBP); 1 ♂ "April 1998 SPAIN// Islas Canarias La Palma// Bco. del Rio upper reaches in// Laurisylva D. T. Bilton leg." "DNA voucher// NHM-IRM7A" (CBP); 1 ♂ “1998 SPAIN Islas Canarias// Tenerife// Barranco del Río 1,600m// D. T. Bilton leg.” “M. IMBRICATA” [HW] “T7// Mel.” [HW] “DNA voucher// NMH-IRM5A” (IBE); 1 ♂ “1998 SPAIN Islas Canarias// Tenerife// Barranco del Río 1,600m// D. T. Bilton leg.” “DNA voucher// NMH-IRM5D” (IBE); 1 ♂ “13/i/2000 SPAIN Islas Canarias// Tenerife// Barranco del Rio 1,600m// D. T. Bilton leg.” "DNA voucher// NHM-IRM17A" (CBP); 1 ♀ “13/i/2000 SPAIN Islas Canarias// Tenerife// Barranco del Rio 1,600m// D. T. Bilton leg.” "DNA voucher// NHM-IRM17B" (CBP). All with “*Meladema imbricata*// (Wollaston, 1871)// D T Bilton [or I Ribera] det. 2017”.

**Additional material examined (non-genotyped specimens). Spain, Canary Islands.** 1 ♂ “April 1998 SPAIN// Islas Canarias la Gomera// El Cedro stream in Garajonay// laurisylva D. T. Bilton leg.” (CBP); 1 ♀ “April 1998 SPAIN// Islas Canarias La Palma// Bco. del Río upper reaches in// Laurisylva D. T. Bilton leg.” (CBP); 1 ♂ “Islas Canarias: Tene-// rife, 9.-10.vi. 1989// Bco. del Río 1100m.// Balke & Hendrich leg.” “Meladema// imbricata// M. Balke det 2011” “M. Balke// BMNH(E) 2013-119” (BMNH); 1 ♂ “I. Canarias/ Tenerife// Bco. del Rio, 1400m// 9.-10.6.1989, Bach// Balke/ Hendrich, leg.” “Meladema// imbricata Woll.// HENDRICH det. 1995” [Latin name & describer HW] (CBP); 2 ♂♂, 2 ♀♀ “9.-10.6.89 Islas Canarias// Tenerife, Barranco// del Rio, ca. 1000m// Balke, Hendrich, Fery leg.” [HW] “Meladema// imbricata// Woll.// Fery det. 89” [HW] (CBF); 2 ♂♂, 1 ♀ “ESP. Tenerife// Bco. del Río 1600m// 2.xi.1991 AN Nilsson” “Meladema imbricata// (Wollaston, 1871)// Det. AN Nilsson 1991” (CBP); 1 ♂ “April 1998 SPAIN// Islas Canarias Tenerife// Bco. del Río Upper// Reaches D. T. Bilton leg” (CBP). All with “*Meladema*// *imbricata* (Wollaston, 1871)// D T Bilton [or I Ribera] det. 2017”.

#### Description

**(based on all material examined).** *Size*: Holotype TL = 22.13 mm; EL = 15.79 mm; MW = 10.22 mm. Other material examined TL = 18.05–21.38 mm; EL = 13.57–15.62 mm; MW = 8.83–9.98 mm.

*Colour*. Dorsum (Figure 3C), dark reddish brown to yellow. Labrum yellowish; clypeus yellowish except central 1/4 red to blackish, connected to dark pigmentation on frons. Frons with transverse pale strip anterolaterally, adjacent to pale parts of clypeus, otherwise dark reddish brown. Medial, paired interocular patches on frons yellow; strongly transverse apicolaterally, almost reaching channel around interior margin of compound eye. Pronotum dark reddish brown on disc; narrowly reddish along anterior margin; lateral margins broadly yellowish to pale red. Elytra yellowish brown, with black irrorations; pattern much more clearly visible when lifted (Figure 4C). Legs yellowish brown to black; posterior tibiae and tarsi darkest. Antennae and maxillary and labial palpi yellowish to reddish. Venter reddish brown; prementum and posterior genae yellow; mentum and submentum reddish. Pronotal hypomeron and shoulder, outer portion of elytral epipleurs and apex of metacoxal process yellowish.

*Head*. Labrum shining, with medium to fine, sparse punctures. Reticulation absent anteriorly, clearly evident in posterior half, here fine and composed of small, isodiametric to slightly transverse meshes. Clypeus weakly shining, with medium to fine, sparse punctures and traces of very fine, shallow, close punctures. Frons weakly shining, entire surface with coarse, open reticulation, becoming stronger and more evident posteriorly. Meshes transverse to isodiametric apically and medially, strongly elongate posteriorly and onto vertex. Paired epicranial foveae on anterior frons, one immediately behind the other. Anterior foveae transverse, posterior foveae elongate oval. Foveae all strongly reticulate; anterior and posterior foveae linked by reticulated channel. Internal and posterior borders of compound eyes distinct, raised relative to level of adjacent cuticle. Lateral margins bordered by distinct narrow channel; deeper anteriorly than posteriorly and continuing behind posterior margin of eye onto vertex. Channel with dense punctures, bearing long, stiff, yellow recumbent to decumbent setae.

*Pronotum*. Posterior margin weakly sinuate laterally (Figure 3C). Surface somewhat shining, strongly rugose. Reticulation meshes large, open, flat and with varying sizes and orientations. Transverse irregular row of medium punctures bearing long, yellow recumbent to decumbent setae 1/5 behind anterior pronotal margin; interrupted briefly in centre, obscured by reticulation inside lateral margins but continuing inside lateral third of posterior margin. Reticulation weak and obsolete anterior to transverse row, surface here clearly doubly punctate, with very fine, dense and medium, sparse to very sparse punctures. Scattered medium punctures visible elsewhere, amongst meshes of reticulation. Centre of disc with traces elongate, narrow, slit-like fovea, typically obscured by reticulation, but traceable as an elongate reticulation channel. Lateral margins slightly raised, shining, without rugose sculpture and with fine, scattered punctures.

*Elytra*. Shining, with short, transverse, usually straight or weakly curved crescentic striolae of varying sizes and density (Figures 5C, 25D, F). Striolae shallow and moderate on shoulder and anterior disc, widely separated; becoming closer, larger and more curved posteriorly. Posterior third of elytra with an almost scaly appearance (Figure 3C); striolae here almost touching each other laterally. Surface between crescentic striolae doubly punctate and reticulate (Figure 5C); with very fine, close and medium, sparse to very sparse punctures (the latter bearing short, peg-like setae). Reticulation fine, somewhat obsolete, meshes isodiametric; more evident in posterior half, sometimes obscuring very fine punctation. Puncture rows well-marked, continuous almost to elytral apices; punctures shallower posteriorly than anteriorly.

*Venter*. Prementum shining, tumid in centre, with fine and medium, sparse punctures. Mentum shining; central projection with shallow median emargination. Lateral lobes with very fine, close punctures, scattered, whitish recumbent to decumbent setae and longitudinal wrinkles. Submentum shining, with transverse wrinkles. Central 1/4 with medium, sparse punctures bearing long, white-yellowish, erect setae. Gula shining, with sparse, shallow, transverse wrinkles; patch of medium-coarse punctures posterolaterally. Genae shining, strongly reticulate; meshes transverse anteriorly and posteriorly, almost isodiametric in centre. Prosternum shining, with weak, low irregular transverse ridges laterally. Arched in centre and with fine, moderate to close punctures laterally, bearing long, white-yellowish, recumbent to erect setae; punctures and setae extending in an irregular row onto process, just below arch. Process lanceolate, arched; apex acuminately rounded. Centre of prosternum and process with double punctation of very fine, close to very close and medium, sparse to moderate punctures. Pronotal hypomeron shining, impunctate. Elytral epipleurs shining, with fine wrinkles; irregular puncture row close to internal margin, from centre to close to apex, punctures bearing fine, whitish, erect setae. Metaventrite shining, central portion with reticulation reduced to sparse, transverse scratches and very fine, close and fine to medium, sparse punctures. Metaventral process strongly reticulate, with transverse to elongate, rugose meshes and traces of fine, sparse punctures; small central area with reticulation of very small meshes. Metaventral process relatively broad; apex acuminate and upturned slightly anterolaterally. Metacoxal lines not reaching anterior border of metacoxae, disappearing approx 1/10 from margin. Internal laminae of metacoxae shining, sculpture as on centre of metaventrite. Metacoxal lobes sculptured as internal laminae, strongly rounded, with irregular, elongate field of medium to coarse punctures close to lateral margins, bearing fine, white, recumbent to erect setae. External laminae of metacoxae shining, smooth close to process, but with strong reticulation elsewhere; reticulation meshes very elongate posteromedially, to transverse anteriorly. Abdominal ventrites shining. Ventrites 3–5 with cluster of golden, erect setae anteromedially. Ventrite 1 with elongate reticulation throughout. Ventrite 2 with similar reticulation; absent close to centre. Ventrite 3 with elongate reticulation laterally, becoming transverse close to smooth central 1/5. Reticulation of ventrites 4 and 5 restricted to lateral third and with superimposed elongate furrows. Ventrites 2–5 doubly punctate; very fine, moderate and fine, sparse to very sparse punctures; punctures most evident in areas without reticulation. Ventrites 3–5 with transverse irregular row of long, yellowish, recumbent to decumbent setae laterally. Ventrite 6 (Figure 11C) with very fine, moderate punctures and medium to coarse, sparse to moderate punctures; punctures coarser close to apex. Elongate, semicircular wrinkles and channels apicolaterally and centrally; apicolateral sculpture extending basally around central portion of ventrite. Some punctures in channels bearing elongate, whitish, erect setae.

*Male*. Foretarsi (Figure 12C) with articulo-setal counts as follows (base to apex): row 1, 8; row 2, 10; row 3, 10; row 4, 8. Curved, golden setae bordering articulo-setal field dense, particularly basally. Foretarsal claws (Figures 12C, 13C) elongate, curved. Mesotarsi (Figure 12G) with articulo-setal counts as follows (base to apex): row 1, 8; row 2, 9–10; row 3, 8–9; row 4, 7 (2 clusters, 4 on inner side, 3 on outer side, situated laterally). Curved, golden setae bordering articulo-setal field relatively dense, especially basally. Mesotarsal claws (Figure 12G) elongate, curved. Abdominal ventrite 6 (Figure 11C) with apex rounded, with well-marked median emargination. Median lobe asymmetrical (Figure 14C), sinuation strong, approximately 1/4–1/3 from apex; ventral margin of apical portion weakly concave in lateral view. Parameres (Figure 14C) with inner margin almost right-angled at base; outer and inner margins undulated slightly.

*Female*. As male, except for simple fore and mesotarsi and differently shaped abdominal ventrite 6 (with bluntly pointed apex - Figure 15C).

*Variation*. The size and density of the crescentic striolae on the elytra differs somewhat between individuals and localities (e.g. Figure 25D, F). At least some of this variation may be due to hybridization with *M. coriacea* (see below), making the extent to which this is truly intraspecific unclear. On La Palma, however, an island with no known populations of *M. coriacea*, and no genetic evidence of hybridization, the crescentic striolae are relatively very large and dense in some females (Figure 25F), approaching the situation seen in some females of *M. lanio* (see below).

#### Differential diagnosis.

Morphologically somewhat intermediate between *M. coriacea*/*M. lepidoptera* sp. n. and *M. lanio*. From *M. coriacea* and *M. lepidoptera* sp. n. *M. imbricata* can be distinguished on its different dorsal colouration, particularly the strongly mottled elytra, with much smaller, sparser crescentic striolae, as well as the less strongly sinuate posterior pronotal margin, details of the male genitalia (median lobe with sinuation further away from apex, with concave ventral margin in lateral view) and the last abdominal ventrites of both sexes. The habitus of *M. imbricata* is also typically more elongate than either of the above species (Figure 3). There are also additional minor differences in dorsal and ventral sculpture, as described above. With the exception of some females (see below), *M. imbricata* can be separated from *M. lanio* on the presence of crescentic striolae on the elytra. The male genitalia of the two species are also different, the sinuation of the median lobe of *M. lanio* occurring further from the apex than *M. imbricata* (see Figure 14C, D). *M. imbricata* also differs from all individuals of *M. lanio* in its less elongate habitus (Figure 3) and the much stronger sculpture of the metacoxae and abdominal ventrites.

**Figure 16.**
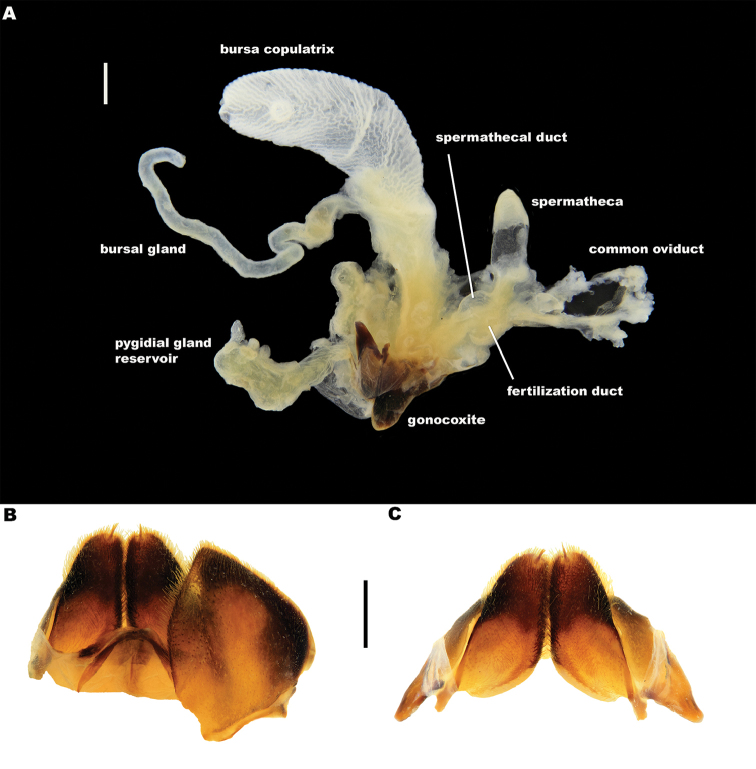
*Meladema coriacea*, Var, France, La-Londe-les-Maures, female reproductive tract and genitalia (DNA voucher codes). **A** reproductive tract anatomy (NHM-IRM11A) **B** gonocoxae and gonocoxosternite (left gonocoxosternite removed) (NHM-IRM11B) **C** gonocoxae with laterotergites expanded (NHM-IRM11B). Scale bars = 1 mm.

**Figure 17.**
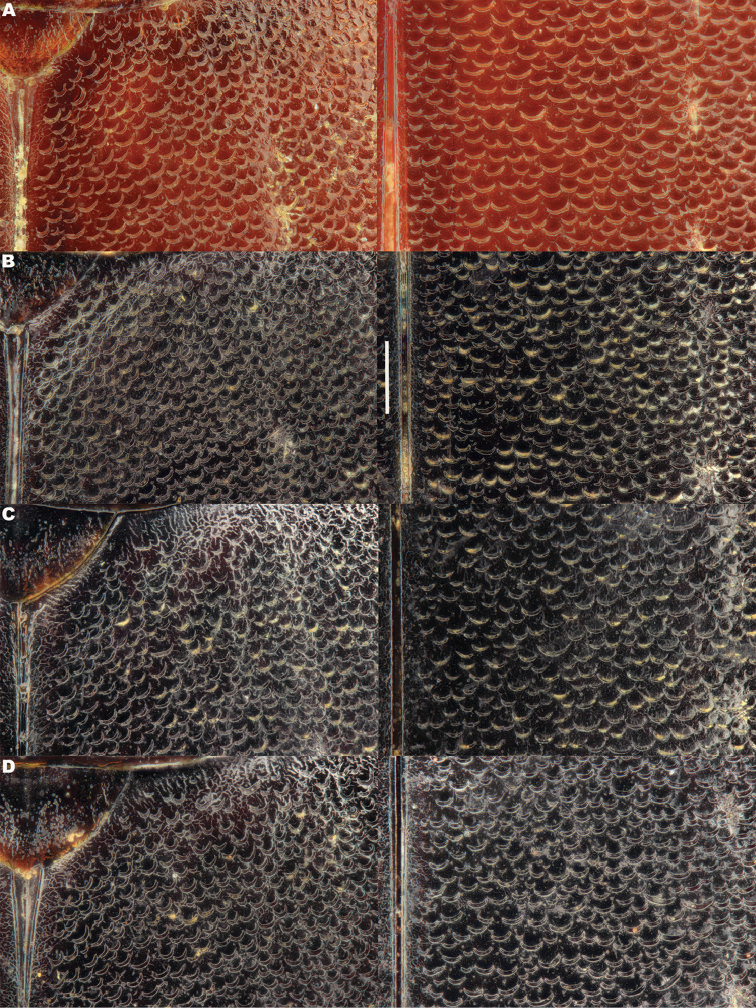
*Meladema coriacea*, Sahara, elytral sculpture; shoulder and middle left and right, respectively. **A** male, Chad, Tibesti, Koudou **B** female, Chad, Tibesti, Bassin de Gorrom **C** male, Algeria, Hoggar, Aguelmanne **D** female, Algeria, Hoggar, Aguelmanne. Scale bar = 0.5 mm.

#### Distribution.

Endemic to the western Canary Islands (Figure 23), being erroneously reported from Madeira in the original description, as discussed by Machado (1987). We have only seen material from a single locality on Tenerife (upper reaches of Barranco del Río), one on La Gomera (El Cedro, in the laurel forest of Garajonay National Park), and two streams on La Palma (Barranco del Río and Barranco Hoyo Verde, situated on opposite sides of the Caldera de Taburiente). Lüderitz et al. (2010) report the species from one additional locality each on La Gomera and Tenerife, although the Tenerife locality (Barranco del Infierno, 500 m) has been visited by DTB in the early 2000s, when it contained only *M. coriacea*, as reported by Malmqvist et al. (1995). Additionally, neither of these records are mentioned by Lüderitz et al. (2016), casting some doubt on both of them. The number of permanent stream systems on the Canary Islands has declined seriously in recent decades, as a result of unsustainable water use. Streams have been variously diverted, piped, dammed, and negatively affected by abstraction directly from aquifers (Malmqvist et al. 1993, 1995, Kelly et al. 2002, Lüderitz et al. 2010, 2016). As a consequence, *M. imbricata*, which appears to be restricted to permanent reaches at relatively high altitude, particularly in forested regions, is very rare, being listed as Critically Endangered (1Ac) in the IUCN Red List (Foster 1996a). The species is also potentially threatened by hybridization with *M. coriacea* (see Ribera et al. 2003 and below), a process which may be further facilitated by ongoing climate change favouring the expansion of this more eurytopic species. Lüderitz et al. (2016) suggest that *M. imbricata* may have disappeared from the El Cedro stream on La Gomera recently, apparently being replaced by *M. coriacea* between 2006 and 2013. It is not clear, however, whether the same stream reaches were sampled on these two occasions. Work establishing the current status of this species in the Canary Islands is clearly a conservation priority.

### Meladema lanio

Taxon classificationAnimaliaColeopteraDytiscidae

(Fabricius, 1775)

Figures 3D , 4D , 5D , 11D , 12D, H , 13D , 14D , 15D , 23 , 24B , 26.

Dytiscus lanio Fabricius, 1775: 231.
Colymbetes lowei Gray, 1831: 284.
Scutopterus lanio (Fabricius, 1775): Dejean 1833: 61; Wollaston 1871: 221.
Colymbetes lanio (Fabricius, 1775): Aubé 1838: 221; Wollaston 1854: 82; Wollaston 1865:68.
Meladema lanio (Fabricius, 1775): Sharp 1882: 632; Régimbart 1895: 184; Gschwedntner, 1936: 42; Falkenstöm 1938: 15; Guignot 1961: 770; Balke and Hendrich 1989: 71.

#### Type locality.

“Maderae aquis”

#### Type material

**(BMNH).** Lectotype ♀ (herein designated, Figure 24B): “Dytiscus lanio// Fab. Entom. p. 231. n. 8.” [Latin name & 231. n. 8. HW] “*Dytiscus lanio*// Fabricius, 1775// LECTOTYPE// DT Bilton & I Ribera des. 2017” (dry pinned, BMNH, Banks Collection). Supposed syntypes comprise the above specimen and one other female, located in the Zoological Museum, University of Copenhagen (Zimsen, 1964; A. Solodovnikov, *pers. comm*.). Sree Selvantharan and Aslak Hansen have kindly communicated a photo of the Copenhagen specimen labelled as *lanio* (both on its pin, and above it in the cabinet). This is in fact a species of *Cybister*.

#### Additional material examined

**(genotyped specimens). Portugal, Madeira**: 1 ♂ “vi/1998 PORTUGAL Madeira// Ribeira dos Cedros// L. C. Kelly leg.” “M3 Hand// Coleoptera” [HW] “Meladema// lanio” [HW] “DNA voucher// NHM-IRM8A” (IBE); 1 ♀ “vi/1998 PORTUGAL Madeira// Levada das Faias// L. C. Kelly leg.” “M2 handsearch// Coleoptera” [HW] “Meladema// lanio” [HW] “DNA voucher// NHM-IRM9A” (IBE); 1 ♀ “vi/1998 PORTUGAL Madeira// Levada das Faias// L. C. Kelly leg.” “DNA voucher// NHM-IRM9B” (IBE); 1 ♂ “Madeira, Canhas// lado sur de Paul da Serra// 1398 m 32°44'36"N 17°05'56"W// charca sobre arroyo de pietras// 19.7.2011 A. Rudoy leg.” “DNA Voucher// IBE-DV298” (IBE). All with “*Meladema*// *lanio* (Fabricius, 1775)// D T Bilton [or I Ribera] det. 2017”

#### Additional material examined

**(non-genotyped specimens). Portugal, Madeira**: 1 ♀ “pouzo” [HW] “var. squamata” [HW] “passage à la m.// imbricatum Woll.” [HW] (ISNB); 1 ♀ “Madeira// 90.32.” [HW] (BMNH); 1 ♀ “Madeira// 90.32.” [HW] “Colymbetes// lanio// Fabr.” [HW] (BMNH); 1 ♀ “Madeira” reverse “48// 99” [small, circular label, HW] “Colymbetes// lanio Fabr.// Madeira” [HW] (BMNH); 1 ♀ “Madeira” reverse “48// 99” [small, circular label, HW] (BMNH); 1 ♂ “Sharp Coll.// 1905-313.” “Dytiscus lanio Fab.// (Scutopterus Woll. Meladema D. S.)// Madeira T. V. Wollaston” [HW] (BMNH); 2 ♂♂, 2 ♀♀ “Madeira// in Fauvel” [HW] “Coll. Odier.// B. M. 1921-288” (BMNH); 3 ♂♂, 3 ♀♀ “MADEIRA// Ribeira da St.// Luzia// 24.VIII.1964// E. W. Classey” [HW] “Brit. Mus.// 1968-48.” [8-48 HW] (BMNH); 3 ♂♂, 3 ♀♀ “MADEIRA:// Ribeira de St. Martinho// 13.VIII.1966// E. W. Classey” [HW] “Brit. Mus.// 1966-446” [6-446 HW] (BMNH); 6 ♂♂, 4 ♀♀ “MADEIRA:// Ribeiro de St.// Martinho// 13.VIII.1966// E. W. Classey” [HW] “Brit. Mus.// 1968-48.” [8-48 HW] (BMNH); 1 ♂, 2 ♀♀ “MADEIRA:// Ribeira da Abilheira// 19/VIII/1966// E. W. Classey” [HW] (BMNH); 2 ♂♂ “23/iii/1995 MADEIRA// Ribera on 103 below// Poiso & Monte ca.// 900m D. T. Bilton leg.” (CBP); 2 ♂♂, 1 ♀ “March 2001 MADEIRA// Levada @ Rabacal// D. T. Bilton leg.” (CBP); 1 ♂ “Madeira 1.-4.8.86// 1kn nördl. Poiso// Quellarn d. R. Frio// leg. Balke// Hendrich” “COLL.// HENDRICH// BERLIN” “Meladema// lanio F.” [HW] (CBP); 3 ♂♂, 3 ♀♀ “Madeira 8 Anchadas da Cruz// 932 m 32°48'35"N 17°17'47"W// charca de arroyo// 4.07.15 A. Rudoy leg.” (IBE); 1 ♂ “Madeira, Canhas// lado sur de Paul da Serra// 1398 m 32°44'36"N 17°05'56"W// charca sobre arroyo de pietras// 19.7.2011 A. Rudoy leg.” (IBE); 1 ♂ “lanio Fab.” [HW] (BMNH); 1 ♂ “40// 4 2// 2156” [small, circular label, HW] (BMNH); 1 ♂ “40// 4 2// 2157” [small, circular label, HW] “C. Lanio Fabr.” (BMNH); 2 ♂♂, 2 ♀♀ without labels (BMNH, Wollaston Collection); 1 ♂ “Meladema// lanio// Fauvel 1897// Madère” [HW] “MUSÉUM PARIS// Coll. P. de Peyerimhoff// 1950” (MNHN); 1 ♂ “MUSÉUM PARIS// Coll. P. de Peyerimhoff// 1950” (MNHN). All with “*Meladema lanio*// (Fabricius, 1775)// D T Bilton [or I Ribera] det. 2017”.

**Figure 18.**
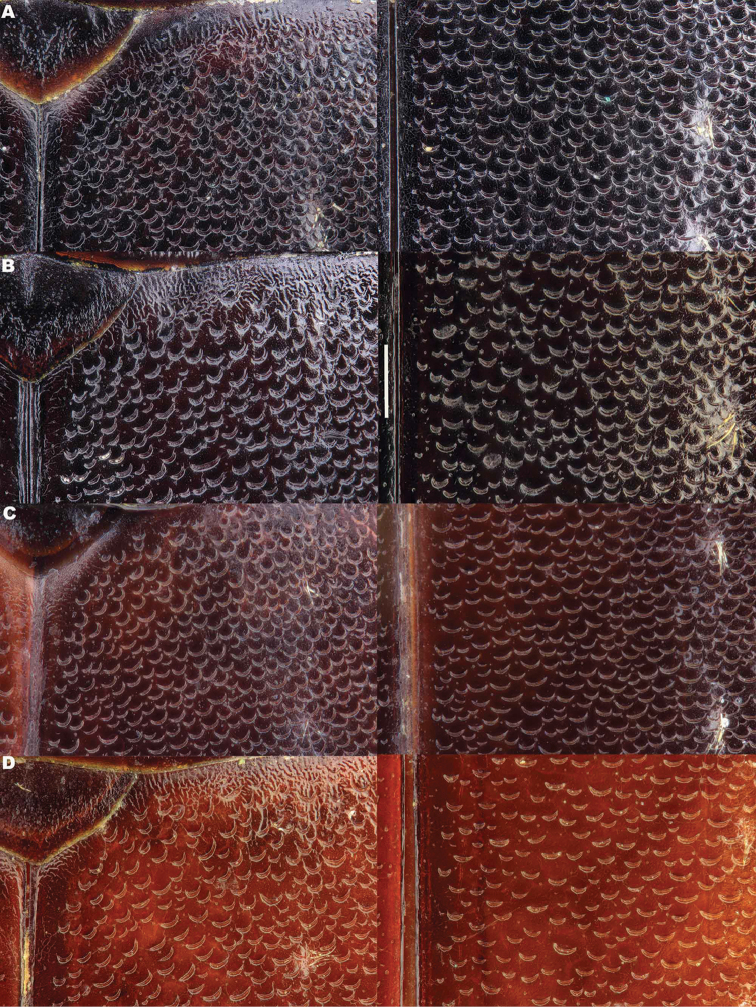
*Meladema* species, Italy, male elytral sculpture; shoulder and middle left and right, respectively (DNA voucher codes, where applicable). **A** *M. lepidoptera* sp. n., Liguria, Levante **B** intermediate specimen, Campania, S. Michele (IBE-AN694) **C** intermediate specimen, Apulia, Vieste **D** *M. coriacea*, Lazio, Grotta di Pastena. Scale bar = 0.5 mm.

**Figure 19.**
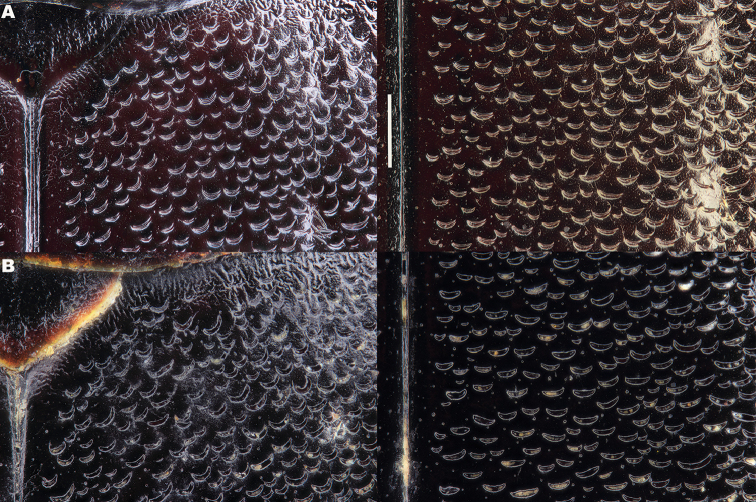
*Meladema coriacea*, Italy, Sicily elytral sculpture; shoulder and middle left and right, respectively (DNA voucher codes, where applicable). **A** male, Bosco Ficuzzo (IBE-AN691) **B** female, Monte Maganoce. Scale bar = 0.5 mm.

#### Description

**(based on all material examined).** *Size*: Lectotype TL = 20.30 mm; EL = 16.30 mm; MW = 10.18 mm. Other material examined TL = 17.40–21.12 mm; EL = 12.67–15.36 mm; MW = 8.70–10.50 mm.

*Colour*. Dorsum (Figure 3D), dark reddish brown to yellow. Labrum yellowish, red anterolaterally in some specimens; clypeus yellowish except central ca. 1/6 red to blackish, connected to dark pigmentation on frons. Frons with transverse pale strip anterolaterally, adjacent to pale parts of clypeus, otherwise dark reddish brown. Medial, paired interocular patches on frons yellow; strongly transverse apicolaterally, almost reaching channel around interior margin of compound eye; confluent in centre in some specimens. Pronotum dark reddish brown on disc; narrowly reddish along anterior margin; lateral margins broadly yellowish. Elytra yellowish brown to greenish brown, with black irrorations; pattern visible without being lifted, but more evident when done so (Figures 3D, 4D). Legs yellowish brown to black; posterior tibiae and tarsi darkest. Antennae and maxillary and labial palpi yellowish to reddish. Venter reddish brown; prementum yellow; posterior genae, mentum and submentum reddish. Pronotal hypomeron and shoulder and outer portion of elytral epipleurs yellowish.

*Head*. Labrum shining, with medium to fine, sparse punctures. Reticulation absent anteriorly, clearly evident in posterior half, here fine and composed of small, isodiametric to transverse meshes. Clypeus weakly shining, with medium to fine, sparse punctures and traces of very fine, shallow, close punctures. Frons weakly shining, entire surface with, open reticulation; weak in front of interocular patches, becoming stronger and more evident posteriorly. Meshes transverse to isodiametric apically and medially, strongly elongate posteriorly and onto vertex. Paired epicranial foveae on anterior frons, one immediately behind the other. Anterior foveae transverse, posterior foveae elongate oval. Foveae reticulate; anterior and posterior foveae linked by reticulated channel. Internal and posterior borders of compound eyes distinct, raised relative to level of adjacent cuticle. Lateral margins bordered by distinct narrow channel; deeper anteriorly than posteriorly and continuing behind posterior margin of eye onto vertex. Channel with dense punctures, bearing long, stiff, yellow recumbent to decumbent setae.

*Pronotum*. Posterior margin weakly sinuate laterally (Figure 3D). Surface somewhat shining, strongly rugose. Reticulation meshes large, open, flat and with varying sizes and orientations. Transverse irregular row of medium punctures bearing long, yellow recumbent to decumbent setae 1/5 behind anterior pronotal margin; interrupted briefly in centre, obscured by reticulation inside lateral margins but continuing inside lateral third of posterior margin. Reticulation weak and obsolete anterior to transverse row, surface here clearly double punctate, with very fine, dense and medium, sparse to very sparse punctures. Scattered medium punctures visible elsewhere, amongst meshes of microreticulation. Centre of disc with elongate, narrow, slit-like fovea, partly obscured by reticulation in some specimens, but then traceable as an elongate reticulation channel. Lateral margins slightly raised, shining, without rugose sculpture and with fine, scattered punctures.

*Elytra*. Shining, without crescentic striolae (except in some females – see below). Surface doubly punctate and reticulate (Figure 5D); with very fine, close and fine to medium, sparse punctures (the latter bearing short, peg-like setae). Reticulation fine, somewhat obsolete, meshes isodiametric; sometimes obscuring very fine punctation. Surface weakly tuberculate towards sides and apex, particularly in posterior third (see Figure 3D). Puncture rows well-marked, continuous almost to elytral apices; punctures shallower posteriorly than anteriorly.

**Figure 20.**
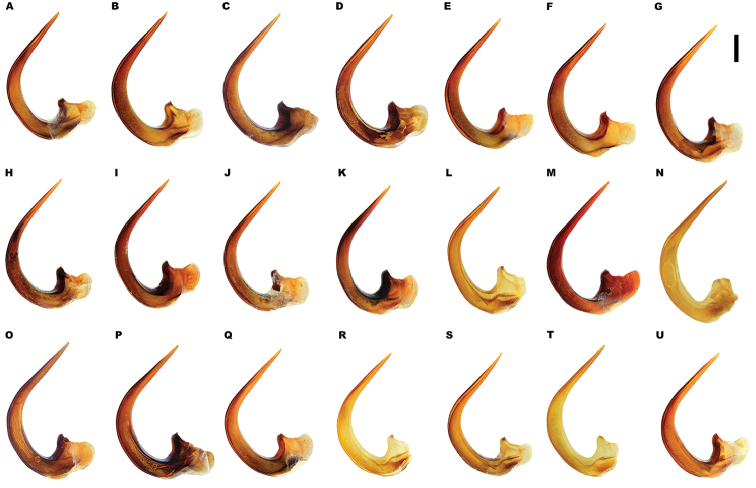
*Meladema* species, variation in male median lobes, lateral view. **A–N** *M. coriacea*; **O–U** *M. lepidoptera* sp. n. (DNA voucher codes, where applicable). **A** Spain, Huesca, Bernués (IBE-DV292) **B** Spain, Girona, Port Bou (IBE-DV-293) **C** Spain, Tarragona, Esblada **D** Spain, Cáceres, nr. Plasencia **E** Spain, Murcia, Fte. Caputa (NHM-IRM13) **F** Spain, Córdoba, Baena (NHM-IRM14C) **G** Tenerife, Chamorga (MNCN-AI1095) **H** Morocco, Oued Massa (NHM-IRM2A) **I** Morocco, Tazekka (NHM-IRM1C) **J** Morocco, Sidi-Ibrahim (NHM-IR7) **K** Algeria, Aïn Damous (MNCN-HI6) **L** Italy, Sicily, Bosco Ficuzzo (IBE-AN691) **M** Greece, Poros, Kampos **N** Greece, Ios **O–P** Corsica, Francardo **Q** holotype, Corsica, Cap Corse (NHM-IRM12E) **R** Italy, Montecristo (IBE-DV289) **S** Sardinia, Villagrande Strìsaili (IBE-RA5) **T** Italy, Elba, Pomonte (IBE-AN692) **U** Italy, Tuscany, S. Luce (IBE-AN693). Scale bar = 1 mm.

**Figure 21.**
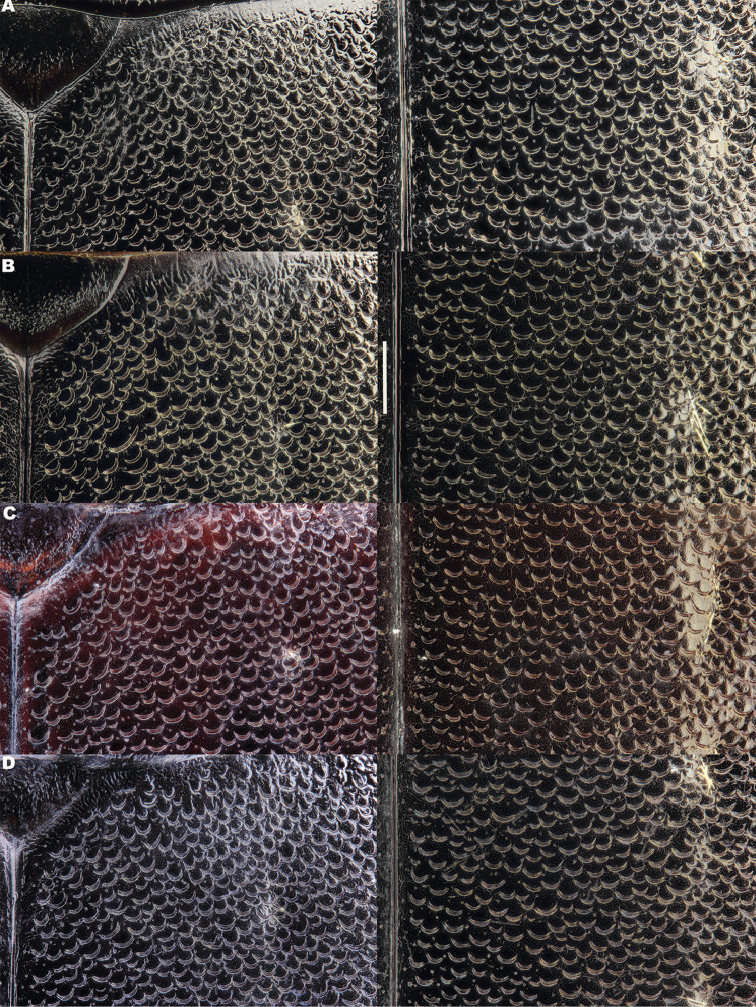
*Meladema lepidoptera* sp. n. elytral sculpture (males); shoulder and middle left and right, respectively (DNA voucher codes). **A** holotype male, Corsica, Cap Corse (NHM-IRM12E) **B** Italy, Montecristo (IBE-DV289) **C** Italy, Elba, Pomonte (IBE-AN692) **D** Italy, Tuscany, S. Luce (IBE-AN693). Scale bar = 0.5 mm.

**Figure 22.**
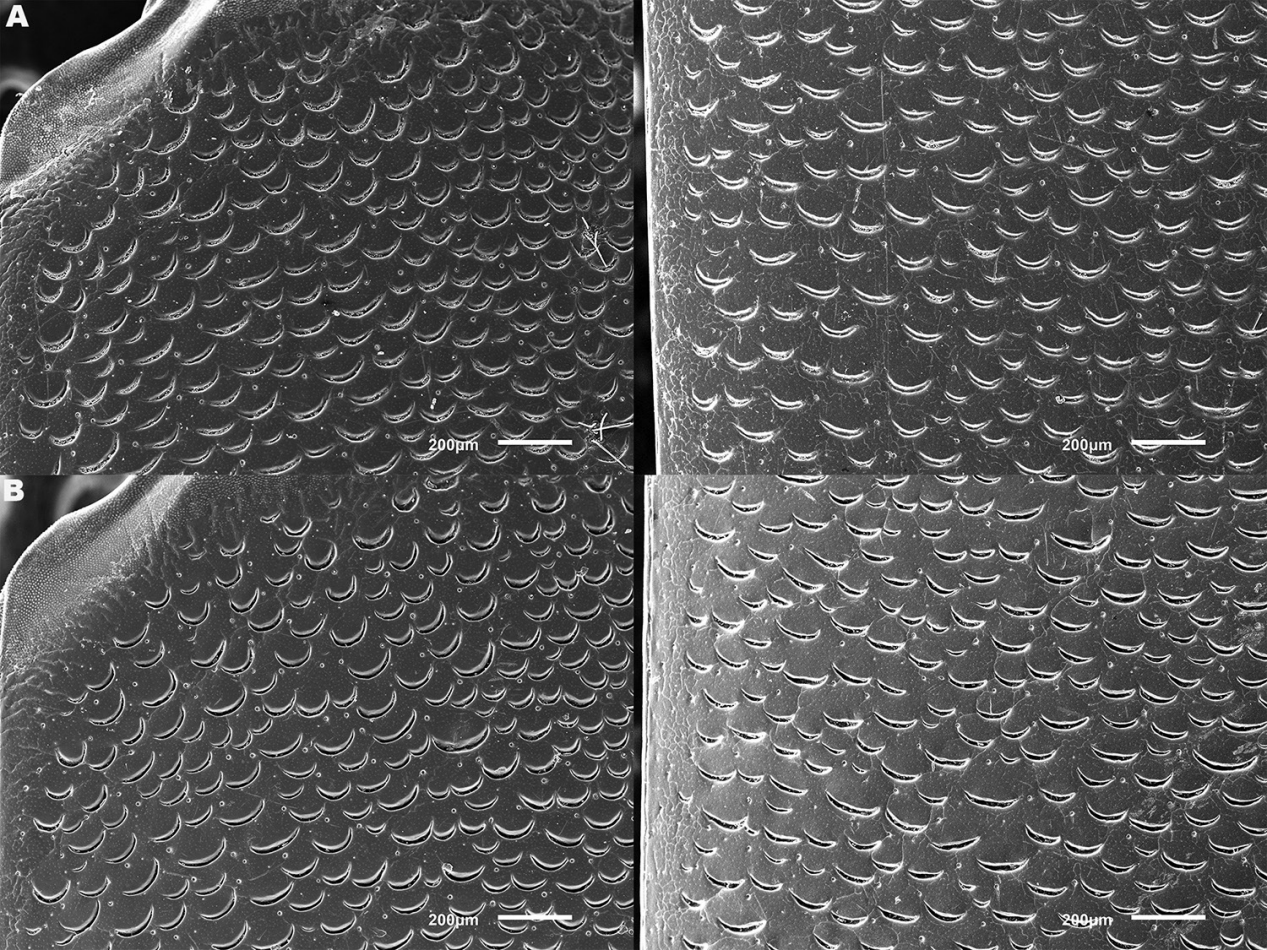
*Meladema lepidoptera* sp. n., Sardinia, elytral sculpture SEMs; shoulder and middle left and right, respectively (DNA voucher codes). **A** male, Villagrande Strìsaili (IBE-RA5) **B** female, Tortoli (IBE-RA18).

*Venter*. Prementum shining, tumid in centre, with fine and medium, sparse punctures. Mentum shining; central projection with shallow median emargination. Lateral lobes with medium, sparse punctures, and scattered, whitish recumbent to decumbent setae. Submentum shining, with transverse wrinkles centrally, and elongate wrinkles laterally. Central 1/4 with medium, sparse punctures bearing long, white-yellowish, erect setae. Gula shining, with sparse, shallow, transverse wrinkles; patch of medium-coarse punctures posterolaterally. Genae shining, with obsolete, open, elongate reticulation. Prosternum shining, with weak, low, irregular transverse ridges laterally. Arched in centre and with fine, moderate to close punctures laterally, bearing long, white-yellowish, recumbent to erect setae; punctures and setae extending in an irregular row onto process, just below arch. Process lanceolate, arched; apex acuminately rounded. Centre of prosternum and process with double punctation of very fine, close to very close and medium, sparse to moderate punctures. Pronotal hypomeron shining, impunctate. Elytral epipleurs shining, with fine wrinkles; irregular puncture row close to internal margin, from centre to close to apex, punctures bearing fine, whitish, erect setae. Metaventrite shining, central portion with reticulation reduced to sparse, transverse scratches and very fine, close and fine to medium, sparse punctures. Metaventral process strongly reticulate, with transverse to elongate, rugose meshes and traces of fine, sparse punctures. Metaventral process relatively broad; apex acuminate and upturned slightly anterolaterally. Metacoxal lines not reaching anterior border of metacoxae, disappearing approx 1/10 from margin. Internal laminae of metacoxae shining, sculpture as on centre of metaventrite. Metacoxal lobes sculptured as internal laminae, strongly rounded, with irregular, elongate field of medium to coarse punctures close to lateral margins, bearing fine, white, recumbent to erect setae. External laminae of metacoxae shining, smooth close to process, and with obsolete reticulation elsewhere; without distinct meshes, wrinkled, elongate around anterior and posterior margins; doubly ounctate, with very fine, close and fine, very sparse punctures. Abdominal ventrites shining. Ventrites 3–5 with cluster of golden, erect setae anteromedially. Ventrite 1 with weak reticulation of elongate scratches throughout. Ventrite 2 with similar reticulation; absent close to centre. Ventrite 3 and 4 with scratches restricted to lateral 1/3. Ventrites 2–5 doubly punctate; very fine, moderate and fine, sparse to very sparse punctures; punctures most evident in areas without reticulation. Ventrites 3–5 with transverse irregular row of long, yellowish, recumbent to decumbent setae laterally. Ventrite 6 (Figure 11D) with very fine, moderate punctures and medium to coarse, sparse to moderate punctures; punctures coarser close to apex. Elongate, semicircular wrinkles and channels apicolaterally and centrally; apicolateral sculpture extending basally around central portion of ventrite. Some punctures in channels bearing elongate, whitish, erect setae.

*Male*. Foretarsi (Figure 12D) with articulo-setal counts as follows (base to apex): row 1, 8–9; row 2, 10; row 3, 10; row 4, 8. Number of setae in rows may differ from right to left tarsus in same beetle. Curved, golden setae bordering articulo-setal field relatively sparse, particularly laterally. Foretarsal claws (Figures 12D, 13D) elongate, curved. Mesotarsi (Figure 12H) with articulo-setal counts as follows (base to apex): row 1, 7–8; row 2, 8; row 3, 8; row 4, 6 (2 clusters of 3, situated laterally). Curved, golden setae bordering articulo-setal field relatively sparse, especially laterally. Mesotarsal claws (Figure 12H) elongate, curved. Abdominal ventrite 6 (Figure 11D) with apex rounded, with well-marked median emargination. Median lobe (Figure 14D) asymmetrical, sinuation strong, approximately 1/3–0.35 from apex; ventral margin of apical portion relatively straight in lateral view. Parameres (Figure 14D) with inner margin almost right-angled at base, with distinct small projection; outer margin undulated slightly; inner straight.

*Female*. As male, except for simple fore and mesotarsi, and differently shaped abdominal ventrite 6 (with bluntly pointed apex - Figure 15D).

*Variation*. Males and females generally have identical sculpture on the elytra. Two females studied, one from Ribeira da St. Luzia (Figure 26B, BMNH) and one from “pouzo” (ISNB) have distinct crescentic striolae, as have been observed in female *M. imbricata* from La Palma (see above). Females sculptured in this manner appear to be relatively rare, the only other occurrences of this form we can find being mentioned by Aubé (1838), who had seen a female with this sculpture (see also Sharp 1882) and Falkenström (1938). Such females may be mistaken for *M. imbricata*, as was the case with the ISNB animal listed above.

**Figure 23.**
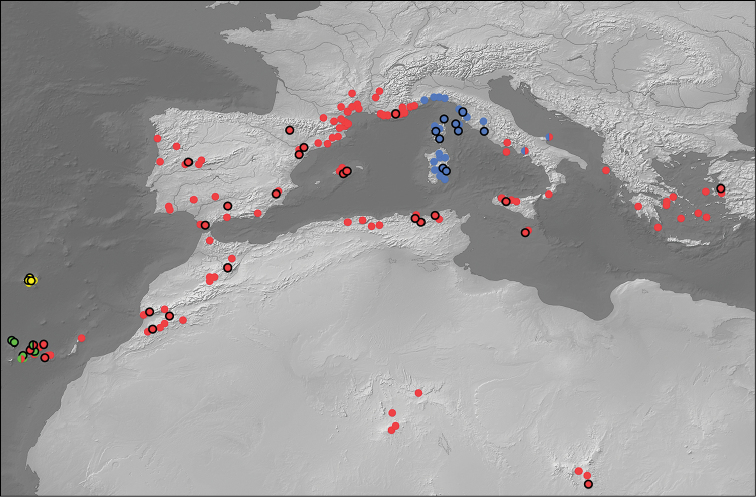
*Meladema* distribution, material examined. Symbols with black border show locations of genotyped specimens. Symbol colours as follows: *M. coriacea* – red; *M. lepidoptera* sp. n. – blue; *M. imbricata* – green; *M. lanio* – yellow. Bicoloured symbols, hybrid or morphologically intermediate individuals.

**Figure 24.**
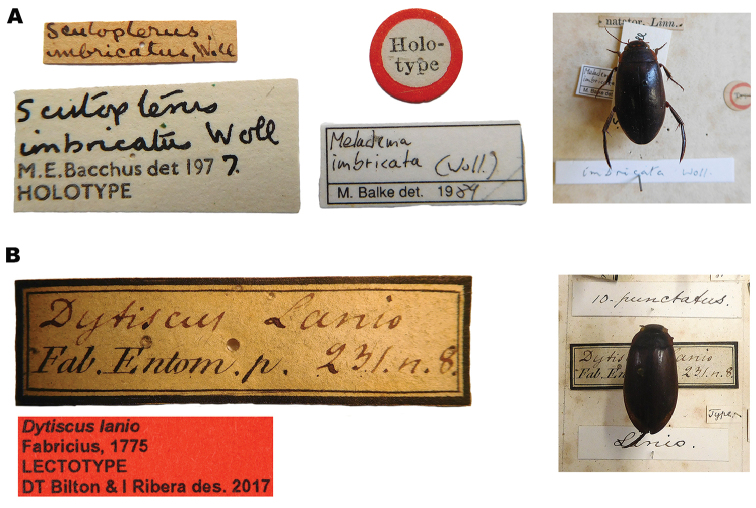
*Meladema* type labels and specimens *in situ*. **A** *M. imbricata*, holotype, BMNH, Wollaston Collection **B** *M. lanio* lectotpe, BMNH, Banks Collection.

**Figure 25.**
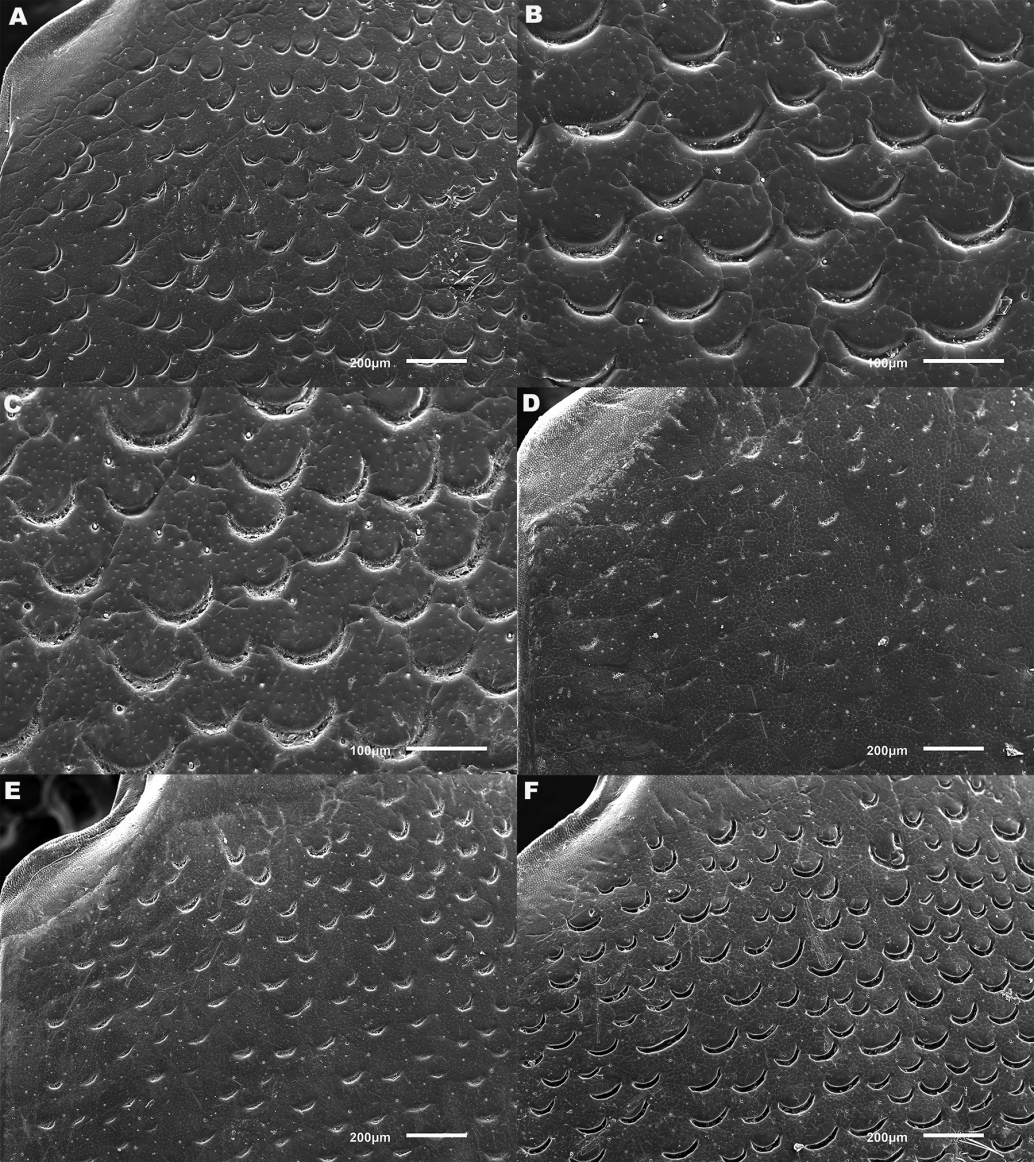
*Meladema* species, Canary Islands, elytral shoulder sculpture SEMs **A–E** males **F** female (DNA voucher codes where applicable). **A–B** *M. coriacea* x *imbricata* hybrid, Tenerife, Bco. del Río 600 m (NHM-IRM16A) **C** *M. coriacea*, Tenerife, Bco. de Masca (NHM-IRM19A) **D** *M. imbricata*, La Gomera, El Cedro (NHM-IRM3A) **E** *M. imbricata* x *coriacea* hybrid, Tenerife, Bco. del Río 1,600 m (NHM-IRM5B) **F** *M. imbricata*, La Palma, Bco. Hoyo Verde.

#### Differential diagnosis.

Closest to *M. imbricata*; for diagnostic characters see under that species.

#### Distribution.

Restricted to the main island of Madeira (Figure 23), where it is relatively widely distributed in permanent streams, particularly in remnant laurel forests in the mountains. Can also be found in man-made levada systems. Much more abundant on Madeira than *M. imbricata* on the Canaries, but still listed as Vulnerable (B1 + 2b) on the IUCN Red List (Foster 1996b), reflecting its very small global range.

### Identification key for *Meladema* species

**Table:** 

1	Dorsum predominantly dark brown to black, unicolourous (Figure 3A, B); reddish in tenerals (e.g. Figure 17A, 18C, D). Sinuation of posterior margin of pronotum relatively strong (Figure 3A, B). Elytra with crescentic striolae relatively large and dense (Figure 5A, B). Median lobe with apical sinuation weak and relatively close to apex (Figure 14A, B)	**2**
–	Dorsum not unicolourous, with elytra distinctly mottled and pronotum with distinct paler margins (Figure 3C, D). Sinuation of posterior margin of pronotum relatively weak (Figure 3C, D). Elytra with crescentic striolae usually small or absent (Figure 5C, D). Median lobe with apical sinuation stronger, situated further from apex (Figure 14C, D)	**3**
2	Crescentic striolae on elytra relatively small and sparse, particularly on shoulder and on disc close to suture; most striolae not contiguous laterally with neighbours (Figures 5A, 7A, C, 8A, C, 9, 10), giving a less scaly appearance overall (Figure 3A). If striolae denser (specimens from central Sahara only), striolae relatively small (Figure 17)	***M. coriacea* Laporte, 1835**
–	Crescentic striolae on elytra relatively larger and denser, particularly on shoulder and on disc close to suture (Figures 5B, 7B, D, 8B, D, 18A, 21, 22), giving a strongly scaly appearance overall (Figure 3B). Many striolae contiguous laterally, especially anteriorly	***lepidoptera* sp. n.**
3	Elytra weakly shining, with (typically small) crescentic striolae in all specimens (Figures 5C, 25D, F). Habitus less elongate (Figure 3C). Elytral colour pattern less evident until lifted (Figures 3C, 4C); paler markings on head less extensive (Figure 3C). Median lobe with sinuation strong, approximately 1/4–1/3 from apex (Figure 14C)	***M. imbricata* (Wollaston, 1871)**
–	Elytra strongly shining, without crescentic striolae in most specimens (Figures 5D, 26). Habitus relatively elongate (Figure 3D). Elytral colour pattern more evident, even without being lifted (Figures 3D, 4D); paler markings on head more extensive (Figure 3D). Median lobe with sinuation strong, approximately 1/3–0.35 from apex (Figure 14D)	***M. lanio* (Fabricius, 1775)**

**Figure 26.**
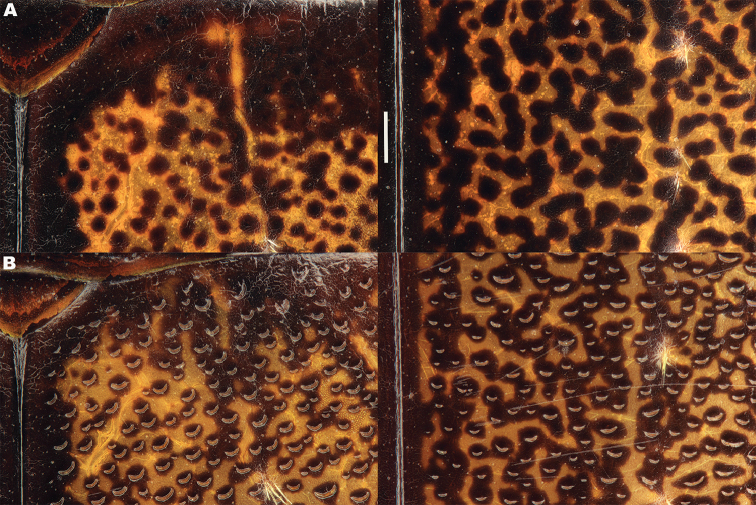
*Meladema lanio*, Madeira, Ribeira da St. Luzia, female elytral sculpture; shoulder and middle left and right, respectively. **A** specimen without and **B** specimen with crescentic striolae. Scale bar = 0.5 mm.

**Figure 27.**
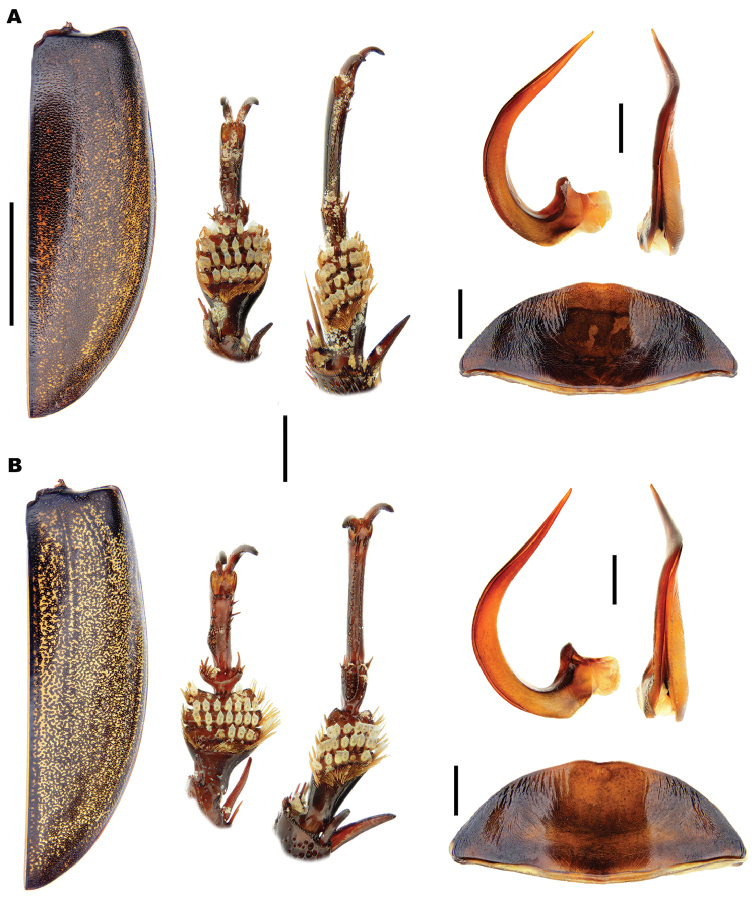
*Meladema* hybrid males, Tenerife, Bco. del Río; isolated elytron, fore and midtarsus, median lobe (lateral and ventral views) and abdominal ventrite 6, respectively (DNA voucher codes). **A** *M. coriacea* x *imbricata*, 600 m (NHM-IRM16A) **B** *M. imbricata* x *coriacea*, 1,600 m (NHM-IRM5B). Scale bars as follows: elytra 5 mm; tarsi, abdominal ventrites, median lobes 1 mm.

## Discussion

It is almost 15 years since it was first recognised that central Mediterranean populations of *Meladema* were genetically divergent from those elsewhere (Ribera et al. 2003). A combination of limited geographical sampling of genotyped individuals, and the apparent absence of diagnostic morphological characters have, however, prevented this lineage from being formally described until now. As with many arthropods, closely related dytiscid species typically are identified most reliably on features of the male genitalia (see e.g. Miller and Bergsten 2016). In these *Meladema*, whilst there is some variation in male genital anatomy, this is of no value in separating *M. coriacea* and *M. lepidoptera* sp. n.; these cryptic species (*sensu* Bickford et al. 2006) instead being diagnosable on subtle, but consistent, differences in elytral sculpture. Our study demonstrates how a combination of mitochondrial and nuclear genetic data is required to make sense of such variation, and highlights the importance of retaining reference material of genotyped individuals for future study. Our work also highlights our limited understanding of freshwater biodiversity in Mediterranean systems, where further undetected but genetically divergent cryptic species with (almost) identical morphologies are likely to occur, particularly in less well studied lineages.

As noted above, *Meladema* specimens from central Saharan mountains (Hoggar, Tassili n’Ajjer, Tibesti) have an elytral sculpture unlike any other material examined. In the absence of more comprehensive genetic data, we treat these beetles here under the widespread *M. coriacea*, but they may represent an additional lineage within the genus. Whilst the Sahara probably originated on closure of the Tethys seaway ca. 7 million years ago (Zhang et al. 2014), the relative extent of desertic conditions has fluctuated considerably since this time, ‘Green Sahara’ periods with extensive vegetation and wetlands being relatively frequent (e.g. Drake et al. 2011, Larrasoaña et al. 2013, Tierney et al. 2017), most recently until around 6,000 BP. Present-day oases in the Sahara support a biota containing a mix of Ethiopian and Palaearctic elements (e.g. Bruneau de Miré and Quézel 1961, Brito et al. 2011, Habel et al. 2013), with isolated populations of a number of aquatic insects, including *Meladema* (Peyerimhoff 1931, Bruneau de Miré and Legros 1963), and some apparent endemics (e.g. Fery and Bouzid 2016). The role of North Africa as a differentiation and speciation centre during the Plio-Pleistocene is increasingly recognised (Husemann et al. 2014) and it is possible that the Green Sahara and its massifs were also important in this process. Genetic studies of *Meladema* and other water beetle populations from these areas would clearly prove illuminating in the future.

### Intermediate specimens and natural hybridization in Meladema

As shown by Ribera et al. (2003), DNA sequence data strongly suggest that *M. coriacea* and *M. imbricata* hybridize in areas where they come into contact on Tenerife. In Barranco del Río, on the south side of the Teide caldera, *coriacea* and *imbricata*-like beetles co-occur, although apparently not in the same stream reach. Specimens identified as *M. coriacea* from 600 m, in a reach with intermittent flow and semi-permanent pools surrounded by xerophytic vegetation, had mtDNA haplotypes characteristic of *M. imbricata* (hereafter referred to as *M. coriacea* x *imbricata* – see Figure 2). Further up the same stream, at 1,600 m, in a reach with permanent flow surrounded by pine forest, two of the four genotyped specimens identified as *M. imbricata* had *M. coriacea* mtDNA (hereafter referred to as *M. imbricata* x *coriacea* – see Figure 2). In contrast to mitochondrial DNA, the nuclear markers (fragments of Histone 3 and Wingless) of hybrid specimens correspond to the species they most resemble on external morphology (see fig. S1 in Sýkora et al. 2017). However, detailed morphological study of these specimens reveals that despite appearing most like one or other species they show some intermediate characteristics, further supporting the hypothesis of their hybrid origin. *M. coriacea* x *imbricata* specimens from 600 m show a number of features suggestive of introgression (see Figures 25A–B, 27), including partial mottling of the elytra (more clearly visible when lifted, as in Figure 27A), a more distinct pale margin to the pronotum, more elongate segment 5 on the fore and mesotarsi, and median lobe with sinuation somewhat further from the apex (Figure 27A). These beetles have articulo-setal counts (base to apex) as follows. Foretarsi: row 1, 7; row 2, 9; row 3, 8; row 4, 6. Mesotarsi: row 1, 7; row 2, 8; row 3, 6; row 4, 5 (2 clusters, 3 on inner side, 2 on outer side, situated laterally). Specimens of *M. imbricata* x *coriacea* from 1,600 m have similar median lobes to the above and somewhat larger crescentic striolae on the elytra (Figure 25E) than most *M. imbricata* seen from elsewhere (the exception being some La Palma females noted above), but are otherwise very close to male *M. imbricata* collected elsewhere in the Canary Islands, albeit with lower articulo-setal counts (base to apex, as follows). Foretarsi: row 1, 7; row 2, 9; row 3, 8; row 4, 7. Mesotarsi: row 1, 8; row 2, 9; row 3, 8; row 4, 5 (2 clusters, 3 on each side, situated laterally). As well as these genotyped individuals, beetles with similar morphology to the *M. coriacea* x *imbricata* hybrids referred to above have also been seen from La Gomera. All specimens examined of apparently hybrid origin are listed below.

Whilst we do not have genetic data to confirm their status, we have seen *Meladema* material from mainland Italy which also suggests that hybridization occurs between *M. coriacea* and *M. lepidoptera* sp. n. in areas where they come into contact. Specimens with elytral sculpture intermediate between the two species (Figure 18B, C) have been seen from Apulia and Campania (see below). Given the fact that *M. coriacea* and *M. imbricata* hybridize on the Canary Islands it seems likely that these beetles also represent individuals of mixed ancestry. The precise geographical limits of *M. coriacea* and *M. lepidoptera* sp. n. in the Italian Peninsula remain unclear, but it is likely that a similar contact zone, with hybridization, may occur (or have occurred) in coastal regions of the Alpi Marittimi, an area where, like much of Mediterranean France, recent coastal development has destroyed many historical *Meladema* localities. Further genetic sampling of Italian *Meladema* is clearly required to confirm this hypothesis.

Hybridization between different *Meladema* taxa is perhaps facilitated by the relatively uniform nature of their genitalia and secondary sexual characteristics (see above). Compared to most dytiscid genera, the male genitalia of *Meladema* species are remarkably similar, particularly the median lobes. *M. coriacea* and *M. imbricata*, for example can be readily distinguished on a suite of external characters, but have median lobes which differ only slightly from each other. In the case of *M. coriacea* and *M. lepidoptera* sp. n., the median lobes are apparently identical in all aspects, as noted above. Despite evidence suggesting some hybridization, we retain these taxa as separate species since they are diagnosible on a suite of both molecular and morphological characters, and appear to remain distinct, suggesting limited gene exchange (Coyne and Orr 2004, DeQueiroz 2007).

Hybrid/intermediate specimens examined are as follows: ***M. coriacea* x *imbricata* hybrids.** Genotyped specimens: 1 ♂ “13/v/2000 SPAIN Tenerife// Bco. del Río 600m// D. T. Bilton leg.” “DNA voucher// NHM-IRM16A” (CBP); 1 ♂ “13/v/2000 SPAIN Tenerife// Bco. del Río 600m// D. T. Bilton leg.” “DNA voucher// NHM-IRM16B” (CBP). Other specimens: 7 ♂♂ “Meladema// hybr. ♂// det. H. Bußler” [Meladema hybr. & ♂ HW] “La Gomera 1.4.94// Bco. de las Hoyas// leg. H Bußler” (CBF); 15 ♀♀ “Meladema// hybr. ♀// det. H. Bußler” [Meladema hybr. & ♀ HW] “La Gomera 1.4.94// Bco. de las Hoyas// leg. H Bußler” (CBF). ***M. imbricata* x *coriacea* hybrids.** Genotyped specimens: 1 ♂ “1998 SPAIN Islas Canarias// Tenerife// Barranco del Río 1,600m// D. T. Bilton leg.” “DNA voucher// NMH-IRM5B” (IBE); 1 ♂ “1998 SPAIN Islas Canarias// Tenerife// Barranco del Río 1,600m// D. T. Bilton leg.” “DNA voucher// NMH-IRM5C” (IBE); 1 ♂ “1998 SPAIN Islas Canarias// Tenerife// Barranco del Río 1,600m// D. T. Bilton leg.” “DNA voucher// NMH-IRM17C” (CBP). **Intermediates between *coriacea* and *lepidoptera* sp. n**: 1 ♂ “Apulien 10.-20.5.65// Vieste.// Budberg” [Vieste HW] (NMW); 1 ♂ “CAMPANIA// S. Michele P.-AV// IX-1996// leg. Pertuzziello I.” “DNA voucher// IBE-AN694” [not possible to amplify any DNA sequences] (CTP).

Veiwed more widely, our study highlights the fact that our fundamental knowledge of biodiversity remains limited, even in the case of comparatively large taxa, in relatively well-studied regions of the world. If we are to understand the origins of such diversity, and how best to protect it in the future, we clearly need accurate taxonomies, which integrative approaches, such as those adopted here, are perhaps best able to supply.

## Supplementary Material

XML Treatment for Meladema

XML Treatment for Meladema coriacea

XML Treatment for Meladema lepidoptera

XML Treatment for Meladema imbricata

XML Treatment for Meladema lanio

## References

[B1] Alarie Y Hughes S (2006) Re-descriptions of larvae of *Hoperius* and *Meladema* and phylogenetic implications for the tribe Colymbetini. Memorie della Società entomologica italiana 85: 307–334. https://doi.org/10.4081/memorieSEI.2006.307

[B2] Aubé C (1836) Iconographie et histoire naturelle des coléoptères d’Europe. Tome Cinquième. Hydrocanthares. Méquignon-Marvis Père et Fils, Paris, 1–64.

[B3] Aubé C (1838) Species général des hydrocanthares et gyriniens; pour faire suite au species général des coléoptères de la collection de M. le comte Dejean. Méquignon Père et Fils, Paris, 804 pp.

[B4] Audisio P Brustel H Carpaneto GM Coletti G Mancini E Trizzino M Antonini G De Biase A (2009) Data on molecular taxonomy and genetic diversification of the European Hermit beetles, a species complex of endangered insects (Coleoptera: Scarabaeidae, Cetoniinae, *Osmoderma*). Journal of Zoological Systematics and Evolutionary Research 47: 88–95. https://doi.org/10.1111/j.1439-0469.2008.00475.x

[B5] Balfour-Browne WAF (1948) *Meladema coriacea* F. (Col., Dytiscidae) in the Canary Islands. Entomologist’s Monthly Magazine 84: 44–45.

[B6] Balke M Hendrich L (1989) Verbreitung. Lebensweise, Taxonomie und Historie der Dytisciden der Ilha da Madeira (Coleoptera, Dytiscidae). Boletim do Museo Municipal do Funchal 41: 55–83.

[B7] Balke M Hendrich L Cuppen GM (1990) Wasserkäfer von den Islas Canarias (Coleoptera: Haliplidae, Dytiscidae, Gyrinidae, Hydrochidae, Hydrophilidae, Hydraenidae, Dryopidae). Entomofauna 11: 349–373.

[B8] Bertrand H (1928) Les larves et nymphes des Dytiscides, Hygrobiides et Haliplides. Encyclopedie entomologique 10: 1–366 [33 pls.]

[B9] Bertrand H (1932a) Captures et élevages de larves de Coléoptères aquatiques (6^e^ note). Annales de la Société entomologique de France 101: 135–140.

[B10] Bertrand H (1932b) Sur deux larves inédites de Dytiscides (Col.). Annales de la Société entomologique de France Livre du Centenaire, 229–236.

[B11] Bickford D Lohman DJ Sodhi NS Ng PKL Meier R Winkler K Ingram KK Das I (2006) Cryptic species as a window on diversity and conservation. Trends in Ecology and Evolution 22: 148–155. https://doi.org/10.1016/j.tree.2006.11.004 17129636 10.1016/j.tree.2006.11.004

[B12] Branden C van den (1885) Catalogue des coléoptères carnassiers aquatiques (Haliplidae, Amphizoidae, Pelobiidae et Dytiscidae). Annales de la Société Entomologique de Belgique 29: 5–116.

[B13] Brito JC Martínez-Freiría F Sierra P Sillero N Tarroso P (2011) Crocodiles in the Sahara Desert: an update of distribution, habitats and population status for conservation planning in Mauritania. PloS ONE 6: e14734. https://doi.org/10.1371/journal.pone.0014734 10.1371/journal.pone.0014734 PMC3045445 21364897

[B14] Bruneau de Miré P Legros C (1963) Les coléoptères hydrocanthares du Tibesti. Bulletin de l’Institut Français d’Afrique Noire (A) 25: 838–894.

[B15] Bruneau de Miré P Quézel P (1961) Remarques taxonomiques et biogéographiques sur la flore des montagnes de la lisière méridionale du Sahara et plus spécialement du Tibesti et du Djebel Marra. Journal d’agriculture tropical et de botanique appliquée 8: 110–133. https://doi.org/10.3406/jatba.1961.6903

[B16] Colgan DJ McLauchlan A Wilson GDF Livingston SP Edgecombe GD Macaranas J Cassis G Gray MR (1998) Histone H3 and U2 snRNA DNA sequences and arthropod molecular evolution. Australian Journal of Zoology 46: 419–437. https://doi.org/10.1071/ZO98048

[B17] Coyne JA Orr HA (2004) Speciation. Sinnauer Associates, Sunderland, Mass, 545 pp.

[B18] Darilmaz MC Kiyak S (2009) Checklist of Gyrinidae, Haliplidae, Noteridae and Dytiscidae of Turkey (Coleoptera: Adephaga). Journal of Natural History 43: 1585–1636. https://doi.org/10.1080/00222930902993682

[B19] Dejean PFMA (1833) Catalogue des coléoptères de la collection de M. le comte Dejean. Livraisons 1 & 2. Méquignon-Marvis Père et Fils, Paris, 176 pp.

[B20] DeQueiroz K (2007) Species concepts and species delimitation. Systematic Biology 55: 879–886. https://doi.org/10.1080/10635150701701083 10.1080/10635150701701083 18027281

[B21] Dettner K (2007) Die adephagen Wasserkäfer der Insel Elba. Entomologie heute 19: 129–140.

[B22] Dincă V Székely L Bálint Z Skolka M Török S Hebert PDN (2017) Improving knowledge of the subgenus Agrodiaetus (Lepidoptera: Lycaenidae: *Polyommatus*) in Eastern Europe: Overview of the Romanian fauna. European Journal of Entomology 114: 179–194. https://doi.org/10.14411/eje.2017.023

[B23] Drake NA Blench RM Armitage SJ Bristow C White KH (2011) Ancient watercourses and biogeography of the Sahara explain the peopling of the desert. Proceedings of the National Academy of Sciences, USA, 108: 458–462. https://doi.org/10.1073/pnas.1012231108 10.1073/pnas.1012231108 PMC3021035 21187416

[B24] Evenhuis NL (2012) François-Luis Comte de Castelnau (1802–1880) and the mysterious disappearance of his original insect collection. Zootaxa 3168: 53–63.

[B25] Fabricius JC (1775) Systema entomologiae, sistens Insectorum classes, ordines, genera, species adiectis synonymis, locis, descriptionibus, observationibus. Flensburgi et Lipsiae: Libraria Korte, xxxii + 832 pp.

[B26] Falkenström GA (1938) Die Arthropodenfauna von Madeira nach den Ergebnissen der Reise von Prof. Dr. O. Lundblad Juli August 1935. IX. Coleoptera: Dytiscidae. Arkiv för Zoologi 30ª Nr. 19: 1–19 [+ 4 pls.]

[B27] Fery H Bouzid S (2016) Notes on *Graptodytes* SEIDLITZ, 1887, re-instatement of *G. laeticulus* (SHARP, 1882) as valid species and description of *Tassilodytes* nov. gen. from Algeria (Coleoptera, Dytiscidae, Hydroporinae, Siettitiina). Linzer biologische Beiträg 48: 451–481.

[B28] Foster G (1996a) *Meladema imbricata* The IUCN Red List of Threatened Species 1996: e.T13014A3406363. http://dx.doi.org/10.2305/IUCN.UK.1996.RLTS.T13014A3406363.en

[B29] Foster G (1996b) *Meladema lanio* The IUCN Red List of Threatened Species 1996: e.T13015A3406416. http://dx.doi.org/10.2305/IUCN.UK.1996.RLTS.T13015A3406416.en

[B30] Franciscolo ME (1979) Coleoptera, Haliplidae, Hygrobiidae, Gyrinidae, Dytiscidae. Fauna d’Italia 14: 1–804.

[B31] Gray G (1831) *Colymbetes lowei*, *Hydaticus marmoratus*. In: Griffith E (Ed.)The animal kingdom arranged in conformity with its organization by the Baron Cuvier with supplementary additions to each order. Volume 14. Whittaker, Treacher and Co., London, 284.

[B32] Gschwendtner L (1936) Monographie der paläarktischen Dytisciden. VII. Colymbetinae (Colymbetini: *Rhantus*, *Nartus*, *Melanodytes*, *Colymbetes*, *Meladema*). Koleopterologische Rundschau 22: 61–102.

[B33] Guéorguiev VB (1987) Coleoptera, hydrocanthares. Fauna na Bulgaria 17: 1–160.

[B34] Guignot F (1932) Les hydrocanthares de France. Les Frères Douladoure, Toulouse, 189–799.

[B35] Guignot F (1961) Revision des hydrocanthares d’Afrique (Coleoptera Dytiscoidea). 3. Annales du Musée Royal du Congo Belge Série 8vo (Sciences Zoologiques) 90: 659–995.

[B36] Habel JC Husemann M Scmitt T Dapporto L Rödder D Vandewoestijne S (2013) A forest butterfly in Sahara desert oases: isolation does not matter. Journal of Heredity 104: 234–247. https://doi.org/10.1093/jhered/ess092 23132908 10.1093/jhered/ess092

[B37] Hebert PDN Cywinska A Bal S Dewaard JR (2003) Biological identifications through DNA barcodes. Proceedings of the Royal Society of London (B) 270: 313–321. https://doi.org/10.1098/rspb.2002.2218 10.1098/rspb.2002.2218 PMC1691236 12614582

[B38] Horn W Kahle I Friese G Gaedike R (1990) Collectiones entomologicae. Ein Kompendium über den Verbleib entomologischer Sammlungen der Welt bis 1960. Akademie der Landwirtschaftswissenschaften der DDR, Berlin, 1; 2: 1–220; 221–573 [38 Taf., 125 Fotos]

[B39] Husemann M Schmitt T Zachos FE Ulrich W Habel JC (2014) Palaearctic biogeography revisited: evidence for the existence of a North African refugium for Western Palaearctic biota. Journal of Biogeography 41: 81–94. https://doi.org/10.1111/jbi.12180

[B40] ICZN (1993) Opinion 1725. *Meladema* Laporte, 1835 (Insecta, Coleoptera): conserved. Bulletin of Zoological Nomenclature 50: 169–170.

[B41] ICZN (1999) International code of zoological nomenclature. 4th ed. International Trust for Zoological Nomenclature, London.

[B42] Kelly LC Rundle SD Bilton DT (2002) Genetic population structure and dispersal in Atlantic Island caddisflies. Freshwater Biology 47: 1642–1650. https://doi.org/10.1046/j.1365-2427.2002.00912.x

[B43] Laporte FLN Caumont de (1835) Études entomologiques. Première partie. Carnassiers. Méquignon-Marvis Père et Fils, Paris, 95–159 [pls. 3–4.]

[B44] Larrasoaña JC Roberts AP Rohling EJ (2013) Dynamics of Green Sahara periods and their role in hominin evolution. PLoS ONE 8: e76514. https://doi.org/10.1371/journal.pone.0076514 10.1371/journal.pone.0076514 PMC3797788 24146882

[B45] Lüderlitz V Langheinrich U Arévalo JR Jüpner R Fernández A (2010) Ecological assessment of streams on La Gomera and Tenerif (Spain) – an approach for an evaluation and restoration tool based on the EU-Water Framework Directive. Waldökologie, Landschaftsforsung und Naturschutz 10: 67–75.

[B46] Lüderlitz V Arévalo JR Fernández-Palacios JM Fernández-Lugo S Eller K Langheinrich U (2016) Freshwater endemic species and the ecological status of streams in the Canary Islands. Journal of Mediterranean Ecology 14: 45–54.

[B47] Machado A (1987) Los ditíscidos de las Islas Canarias (Coleoptera, Dytiscidae). Instituto de Estudios Canarios, La Laguna, 81 pp.

[B48] Michat MC (2005) Larval morphology and phylogenetic relationships of *Bunites distigma* (Brullé) (Coleoptera: Dytiscidae: Colymbetinae: Colymbetini). The Coleopterists Bulletin 59: 433–447. https://doi.org/10.1649/797.1

[B49] Miller KB (2001) On the phylogeny of the family Dytiscidae Linnaeus (Insecta: Coleoptera) with an emphasis on the morphology of the female reproductive tract. Insect Systematics and Evolution 32: 45–92. https://doi.org/10.1163/187631201X00029

[B50] Miller KB Bergsten J (2014) The phylogeny and classification of diving beetles (Coleoptera: Dytiscidae) In: Yee DA (Ed.) Ecology, systematics and natural history of predaceous diving beetles (Coleoptera: Dytiscidae). Springer, New York, 49–172.

[B51] Miller KB Bergsten J (2016) Diving beetles of the World. John Hopkins University Press, Baltimore, 336 pp.

[B52] Miller KB Nilsson AN (2003) Homology and terminology: communicating information about rotated structures in water beetles. Latissimus 17: 1–4.

[B53] Miller MA Pfeiffer W Schwartz T (2010) Creating the CIPRES Science Gateway for inference of large phylogenetic trees. In: Proceedings of the Gateway Computing Environments Workshop (GCE). New Orleans, 1–8. https://doi.org/10.1109/GCE.2010.5676129

[B54] Morinière J Michat MC Jäch MA Bergsten J Hendrich L Balke M. (2015) Anisomeriini diving beetles – An Atlantic-Pacific Island disjunction on Tristan da Cunha and Robinson Crusoe Island, Juan Fernández? Cladistics 31: 66–76. 10.1111/cla.12074 34758583

[B55] Morinière J Van Dam MH Hawlitschek O Bergsten J Michat MC Hendrich L Ribera I Toussaint EFA Balke M (2016) Phylogenetic niche conservatism explains an inverse latitudinal diversity gradient in freshwater arthropods. Scientific Reports 6: 26340. https://doi.org/10.1038/srep26340 10.1038/srep26340 PMC4877923 27215956

[B56] Nilsson AN Hájek J (2017a) Catalogue of Palearctic Dytiscidae (Coleoptera). Internet version 2017-01-01. http://www.waterbeetles.eu

[B57] Nilsson AN Hájek J (2017b) A World Catalogue of the Family Dytiscidae Version 31.I.2017. http://www.waterbeetles.eu

[B58] Nilsson AN Hilsenhoff WL (1991) Review of first-instar larvae of Colymbetini (Coleoptera: Dytiscidae), with a key to genera and phylogenetic analysis. Entomologica scandinavica 22: 35–44. https://doi.org/10.1163/187631291X00291

[B59] Peyerimhoff PM de Fontenelle (1931) Mission scientifique du Hoggar. Coléoptères. Mémoires de la Société d’Histoire Naturelle de l’Afrique du Nord 2: 1–173.

[B60] Poggi R (1976) Materiali per un elenco dei Coleotteri dell’isola di Montecristo (Archipelago toscano) con descrizione di un nuovo Stafilinide (*Leptotyphlus oglasensis* n. sp.). Biogeographia 5: 609–635. https://doi.org/10.21426/B65110051

[B61] Régimbart M (1895) Révision des Dytiscidae et Gyrinidae d’Afrique, Madagascar et îles voisines. Mémoires de la Société Entomologique de Belgique 4: 1–244.

[B62] Ribera I Bilton DT Vogler AP (2003) Mitochondrial DNA phylogeography and population history of *Meladema* diving beetles on the Atlantic Islands and in the Mediterranean basin. Molecular Ecology 12: 153–167. https://doi.org/10.1046/j.1365-294X.2003.01711.x 12492885 10.1046/j.1365-294x.2003.01711.x

[B63] Ribera I Fresneda J Bucur R Izquierdo A Vogler AP Salgado JM Cieslak A (2010) Ancient origin of a Western Mediterranean radiation of subterranean beetles. BMC Evolutionary Biology 10: 29. https://doi.org/10.1186/1471-2148-10-29 10.1186/1471-2148-10-29 PMC2834687 20109178

[B64] Sanfilippo N (1966) I ditiscidi dell’isola di Madera e notize sulla fauna acquatica associata. Bollettino della Società entomologica italiana 96: 46–54.

[B65] Sharp D (1882) On aquatic carnivorous Coleoptera or Dytiscidae. Scientific Transactions of the Royal Dublin Society 2: 179–1003 [pls. 7–18]

[B66] Sharp D (1901) The types of Heer’s Fauna Coleoptorum Helvetica. Entomologist’s Monthly Magazine 37: 143–144.

[B67] Simon C Frati F Beckenbach AT Crespi B Liu H Flook P (1994) Evolution, weighting, and phylogenetic utility of mitochondrial gene sequences and a compilation of conserved polymerase chain reaction primers. Annals of the Entomological Society of America 87: 651–701. https://doi.org/10.1093/aesa/87.6.651

[B68] Stamatakis A Hoover P Rougemont J (2008) A rapid bootstrap algorithm for the RAxML web servers. Systematic Biology 57: 758–771. https://doi.org/10.1080/10635150802429642 18853362 10.1080/10635150802429642

[B69] Sýkora V García-Vázquez D Sánchez-Fernández D Ribera I (2017) Range expansión and ancestral niche resonstruction in the Mediterranean diving beetle genus *Meladema* (Coleoptera, Dytiscidae). Zoologica Scripta 46: 445–458. https://doi.org/10.1111/zsc.12229

[B70] Tierney JE Pausata FSR deMenocal PB (2017) Rainfall regimes of the Green Sahara. Science Advances 3: e1601503. https://doi.org/10.1126/sciadv.1601503 10.1126/sciadv.1601503 PMC5242556 28116352

[B71] Touaylia S Garrido J Bejaoui M Boumaiza M (2011) Altitudinal distribution of aquatic beetles (Coleoptera) in northern Tunisia: relationship between species richness and altitude. The Coleopterists Bulletin 65: 53–62. https://doi.org/10.1649/0010-065X-65.1.53

[B72] Wollaston TV (1854) Insecta maderensia; being an account of the insects of the islands of the Madeiran group. Taylor & Francis, London, 634 pp. [13 pls.]

[B73] Wollaston TV (1865) Coleoptera Atlantidum, being an enumeration of the coleopterous insects of the Madeiras, Salvages, and Canaries. Taylor & Francis, London, 526 pp. [Appendix 140 pp.]

[B74] Wollaston TV (1871) On additions to the Atlantic Coleoptera. Transactions of the Entomological Society of London 1871: 203–314.

[B75] Zauli A Carpaneto GM Chiari S Mancini E Nyabuga FN De Zan LR Romiti F Sabbani S Audisio PA Hedenström E Bologna MA Svensson GP (2016) Assessing the taxonomic status of *Osmoderma cristinae* (Coleoptera: Scarabaeidae), endemic to Sicily, by genetic, morphological and pheromonal analyses. Journal of Zoological Systematics and Evolutionary Research 54: 206–214. https://doi.org/10.1111/jzs.12127

[B76] Zhang Z Ramstein G Schuster M Li C Contoux C Yan Q (2014) Aridification of the Sahara desert caused by Tethys Sea shrinkage during the Late Miocene. Nature 513: 401–404. https://doi.org/10.1038/nature13705 25230661 10.1038/nature13705

[B77] Zimsen E (1964) The type material of I. C. Fabricius. Munksgaard, Copenhagen, 656 pp.

